# Supplementary material for: Exploiting metabolic vulnerability in glioblastoma using a brain-penetrant drug with a safe profile
Source: EMBO Mol Med. 2025 Feb 3;17(3):469–503. doi: 10.1038/s44321-025-00195-6 (PMC11903783; doi:10.1038/s44321-025-00195-6)
Supplement: Supplementary file 1 — Appendix [file 44321_2025_195_MOESM1_ESM.pdf]

Table of content:

**Appendix Figure**	**Title**	**Page number**
Appendix Figure S1	Mubritinib downregulates cell-cycle related pathways in BTSCs.	**2**
Appendix Figure S2	Mubritinib does not impact normal non-oncogenic cells and has a safe profile *in vivo*.	**3**
Appendix Table S1	Characterization of patient-derived BTSCs.	**4**
Appendix Table S2	Mass spectrometer settings for the quantification of mubritinib by UPLC-MS/MS.	**6**

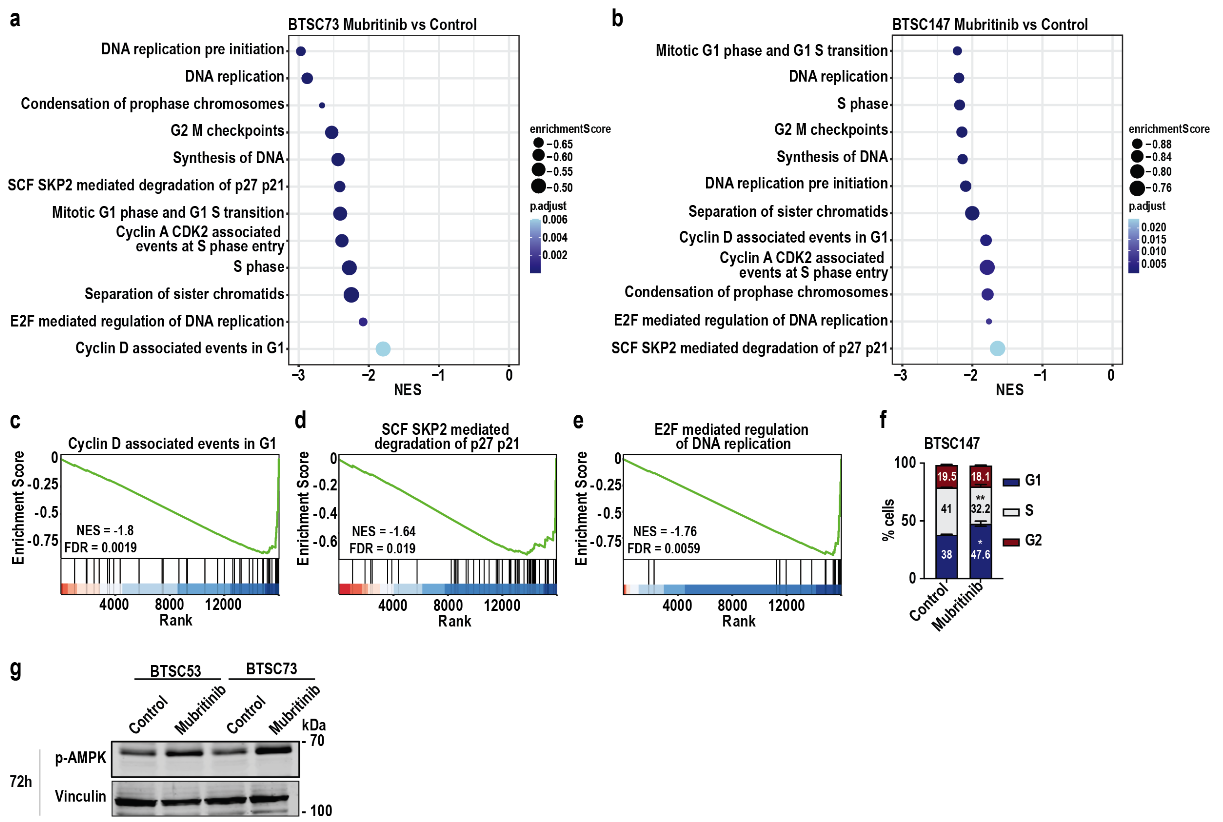

Appendix Fig S1. Mubritinib downregulates cell-cycle related pathways in BTSCs.

(**a-b**) Gene set enrichment analysis of deregulated genes in BTSC73 (a) and BTSC147 (b) treated with 500 nM of mubritinib for 24 h demonstrates enrichment for gene sets corresponding to cell-cycle related pathways.

(**c-e**) Gene set enrichment analysis of deregulated genes in BTSC147 treated with 500 nM mubritinib for 24 h demonstrates enrichment of gene sets corresponding to cyclin D1 associated events in G1 (c), SCF SKP2 mediated degradation of p27/p21 (d) and E2F mediated regulation of DNA replication (e).

(**f**) Cell cycle distribution was assessed by flow cytometry after PI staining in BTSC147 following 24 h of treatment with 500 nM of mubritinib. Data are presented as the means ± SEM, n = 3 independent biological experiments. Unpaired two-tailed t test. *p_{G1} = 0.0128, **p_S = 0.0074.

(**g**) BTSC53 and BTSC73 were treated for 72 h with 500 nM of mubritinib or vehicle control and subjected to immunoblotting using antibody against p-AMPK. Tubulin was used as loading control.

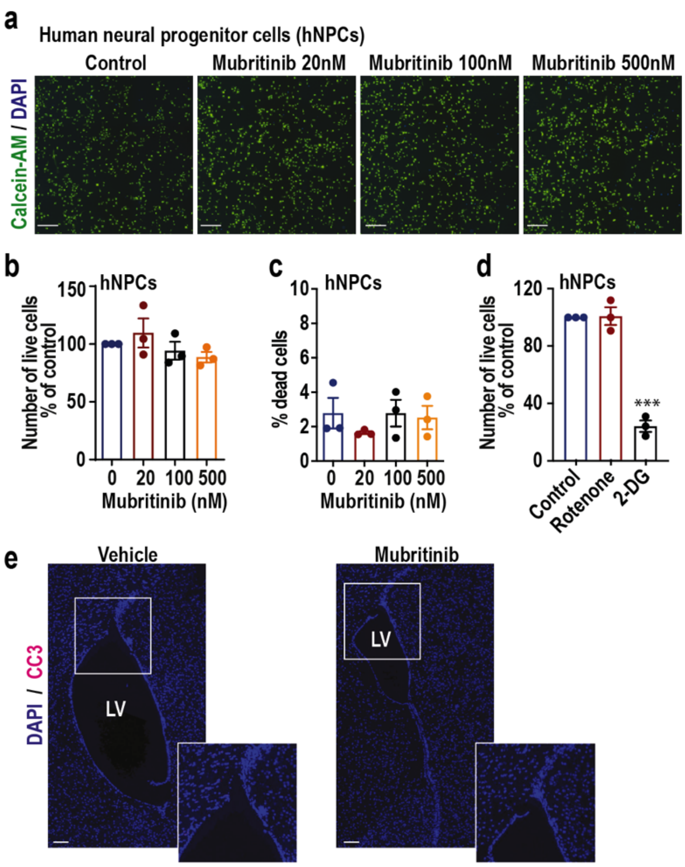

Appendix fig S2. Mubritinib does not impact normal non-oncogenic cells and has a safe profile *in vivo*.

(**a-c**) Human neural progenitor cells (hNPCs) were cultured in 2D monolayer on matrigel-coatted plate and treated with increasing concentrations of mubritinib (0 to 500 nM) for 4 days and subjected to calcein-AM/DAPI double staining. Representative images are shown (a). Scale bars = 100 µm. The number of live cells (calcein-AM positive) (b) and dead cells (DAPI positive) (c) were quantified by Fiji software.

(**d**) hNPCs were treated with 100 nM rotenone or 1mM 2-Deoxy-D-glucose (2-DG) and subjected to live cell counting by PI staining followed by flow cytometry after 7 days of treatment. Data are presented as the mean ± SEM. One-way ANOVA followed by Dunnett's test vs control. ***$p_{2\text{-DG}}$ = 2.6e-5.

(**e**) Representative immunofluorescence images of cleaved caspase 3 (CC3) in the SVZ of mouse brains subjected to the indicated treatments are shown. Scale bars = 100 µm.

BTSCs	Diagnosis	Age, y	Sex	IDH1	EGFR status	TP53	PTEN	NF1	CDKN2A	MGMT	References
**12**	GB-rec	59	M	wt	wt	mut	mut	NA	NA	U	(Cusulin *et al*, 2015; Kelly *et al*, 2009)
**25**	GB	57	M	wt	wt	mut	mut	NA	NA	U/M	(Cusulin *et al.*, 2015; Kelly *et al.*, 2009)
**50**	GB	61	M	wt	amplified	wt	mut	wt	homo del	M	(Cusulin *et al.*, 2015; Luchman *et al*, 2014)
**53**	GB	59	M	wt	amplified / mut G598V	mut	wt	wt	homo del	M	(Cusulin *et al.*, 2015; Kelly *et al.*, 2009; Stechishin *et al*, 2013)
**73**	GB	52	M	wt	amplified / VIII	mut	mut	NA	homo del	M	(Cusulin *et al.*, 2015; Kelly *et al.*, 2009; Stechishin *et al.*, 2013)
**75**	GB	74	M	wt	wt	wt	wt	wt	wt	U	(Cusulin *et al.*, 2015; Kelly *et al.*, 2009; Shen *et al*, 2019; Stechishin *et al.*, 2013)
**100**	GB	63	M	wt	wt	wt	wt	NA	NA	U	(Cusulin *et al.*, 2015; Shen *et al.*, 2019)
**119**	GB-rec	69	F	wt	amplified / mut Y620C	mut	mut	mut	homo del	U/M	(Nguyen *et al*, 2014; Shen *et al.*, 2019)
**147**	GB-rec	56	M	wt	VIII	mut	mut	wt	homo del	U	(Cusulin *et al.*, 2015; Stechishin *et al.*, 2013)
**198**	GB	52	F	wt	amplified	mut	mut	wt	NA	U/M	(Grinshtein *et al*, 2016; Shen *et al.*, 2019)
**P3**	GB	NA	NA	wt	wt	wt	mut	wt	homo del	U	(Eskilsson *et al*, 2016; Joseph *et al*, 2022; Keunen *et al*, 2011)

Appendix Table S1: Characterization of patient-derived BTSCs.

Mutant (mut) or wild-type (WT) status of genes frequently mutated in GB for the BTSC lines used in this study isolated from primary glioblastoma tumour (GB) or recurrent glioblastoma (GB-rec). vIII indicates EGFR variant III, homo del indicates a homozygous deletion, NA – not available; U indicates unmethylated; M indicates methylated.

References

Cusulin C, Chesnelong C, Bose P, Bilenky M, Kopciuk K, Chan JA, Cairncross JG, Jones SJ, Marra MA, Luchman HA *et al* (2015) Precursor States of Brain Tumor Initiating Cell Lines Are

Predictive of Survival in Xenografts and Associated with Glioblastoma Subtypes. *Stem Cell Reports* 5: 1-9

Eskilsson E, Rosland GV, Talasila KM, Knappskog S, Keunen O, Sottoriva A, Foerster S, Solecki G, Taxt T, Jirik R *et al* (2016) EGFRvIII mutations can emerge as late and heterogenous events in glioblastoma development and promote angiogenesis through Src activation. *Neuro Oncol* 18: 1644-1655

Grinshtein N, Rioseco CC, Marcellus R, Uehling D, Aman A, Lun X, Muto O, Podmore L, Lever J, Shen Y *et al* (2016) Small molecule epigenetic screen identifies novel EZH2 and HDAC inhibitors that target glioblastoma brain tumor-initiating cells. *Oncotarget* 7: 59360-59376

Joseph JV, Magaut CR, Storevik S, Geraldo LH, Mathivet T, Latif MA, Rudewicz J, Guyon J, Gambaretti M, Haukas F *et al* (2022) TGF-beta promotes microtube formation in glioblastoma through thrombospondin 1. *Neuro Oncol* 24: 541-553

Kelly JJ, Stechishin O, Chojnacki A, Lun X, Sun B, Senger DL, Forsyth P, Auer RN, Dunn JF, Cairncross JG *et al* (2009) Proliferation of human glioblastoma stem cells occurs independently of exogenous mitogens. *Stem Cells* 27: 1722-1733

Keunen O, Johansson M, Oudin A, Sanzey M, Rahim SA, Fack F, Thorsen F, Taxt T, Bartos M, Jirik R *et al* (2011) Anti-VEGF treatment reduces blood supply and increases tumor cell invasion in glioblastoma. *Proc Natl Acad Sci U S A* 108: 3749-3754

Luchman HA, Stechishin OD, Nguyen SA, Lun XQ, Cairncross JG, Weiss S (2014) Dual mTORC1/2 blockade inhibits glioblastoma brain tumor initiating cells in vitro and in vivo and synergizes with temozolomide to increase orthotopic xenograft survival. *Clin Cancer Res* 20: 5756-5767

Nguyen SA, Stechishin OD, Luchman HA, Lun XQ, Senger DL, Robbins SM, Cairncross JG, Weiss S (2014) Novel MSH6 mutations in treatment-naive glioblastoma and anaplastic oligodendroglioma contribute to temozolomide resistance independently of MGMT promoter methylation. *Clin Cancer Res* 20: 4894-4903

Shen Y, Grisdale CJ, Islam SA, Bose P, Lever J, Zhao EY, Grinshtein N, Ma Y, Mungall AJ, Moore RA *et al* (2019) Comprehensive genomic profiling of glioblastoma tumors, BTICs, and xenografts reveals stability and adaptation to growth environments. *Proc Natl Acad Sci U S A* 116: 19098-19108

Stechishin OD, Luchman HA, Ruan Y, Blough MD, Nguyen SA, Kelly JJ, Cairncross JG, Weiss S (2013) On-target JAK2/STAT3 inhibition slows disease progression in orthotopic xenografts of human glioblastoma brain tumor stem cells. *Neuro Oncol* 15: 198-207

Analyte	Parent ion (m/z)	Transition ion (m/z)	Confirmation ion (m/z)	Cone voltage (V)	Collision energy (eV)
Mubritinib	469.2	252.3	224.2	30	25 / 20
[^{13}C, $^{2}H_3$]-Sorafenib	469.3	274.2	256.2	50	30 / 25

Appendix Table S2. Mass spectrometer settings for the quantification of mubritinib by UPLC-MS/MS.
