# Supplementary figures and images for: Exploiting metabolic vulnerability in glioblastoma using a brain-penetrant drug with a safe profile (part 1 of 2)
Source: EMBO Mol Med. 2025 Feb 3;17(3):469–503. doi: 10.1038/s44321-025-00195-6 (PMC11903783; doi:10.1038/s44321-025-00195-6)

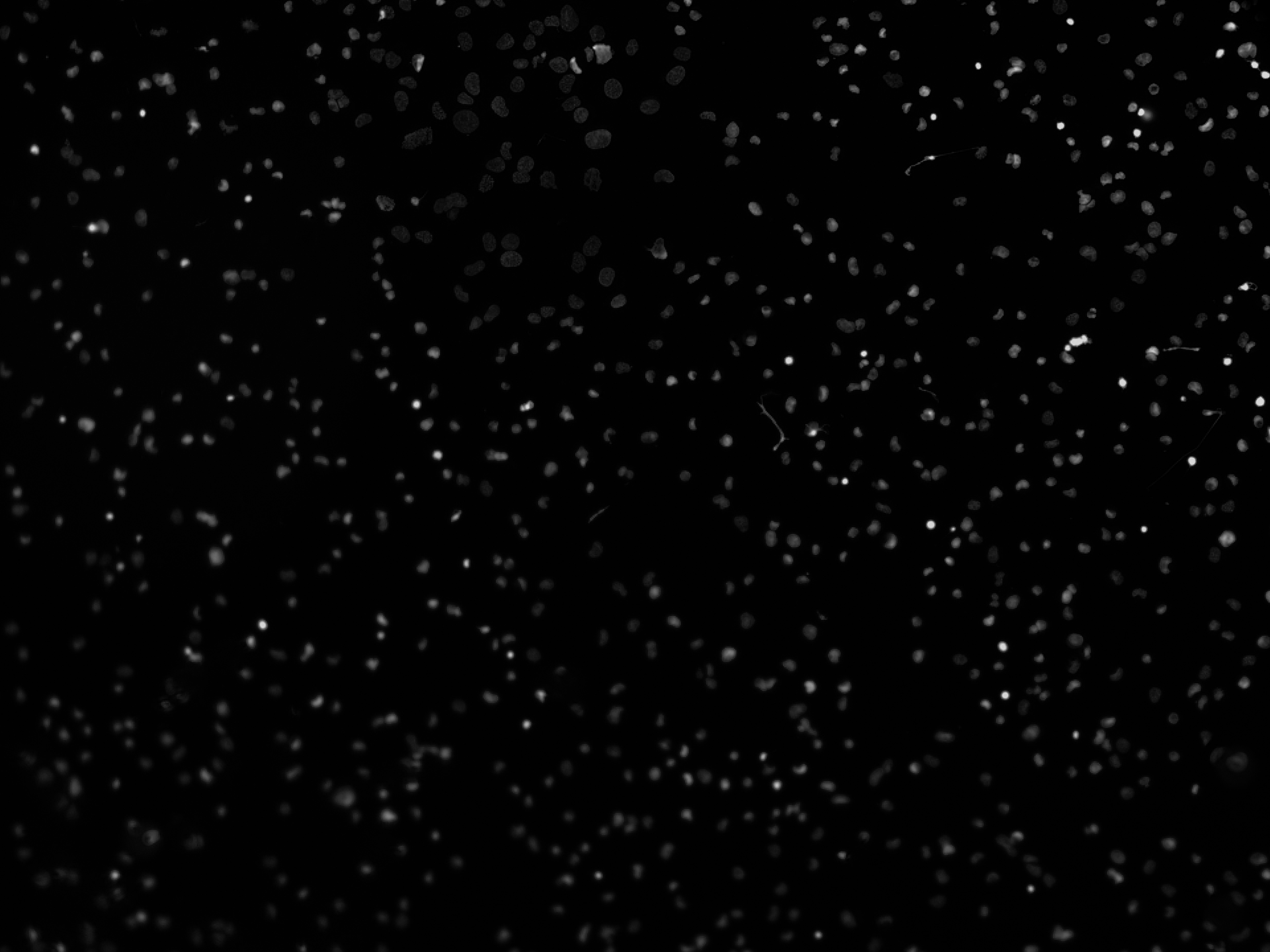

Supplement: Supplementary file 7 — Source data Fig. 1 [file 44321_2025_195_MOESM7_ESM.zip › Figure 1/1G/BTSC73/BTSC73 Mubritinib 500 nM DAPI.tif]

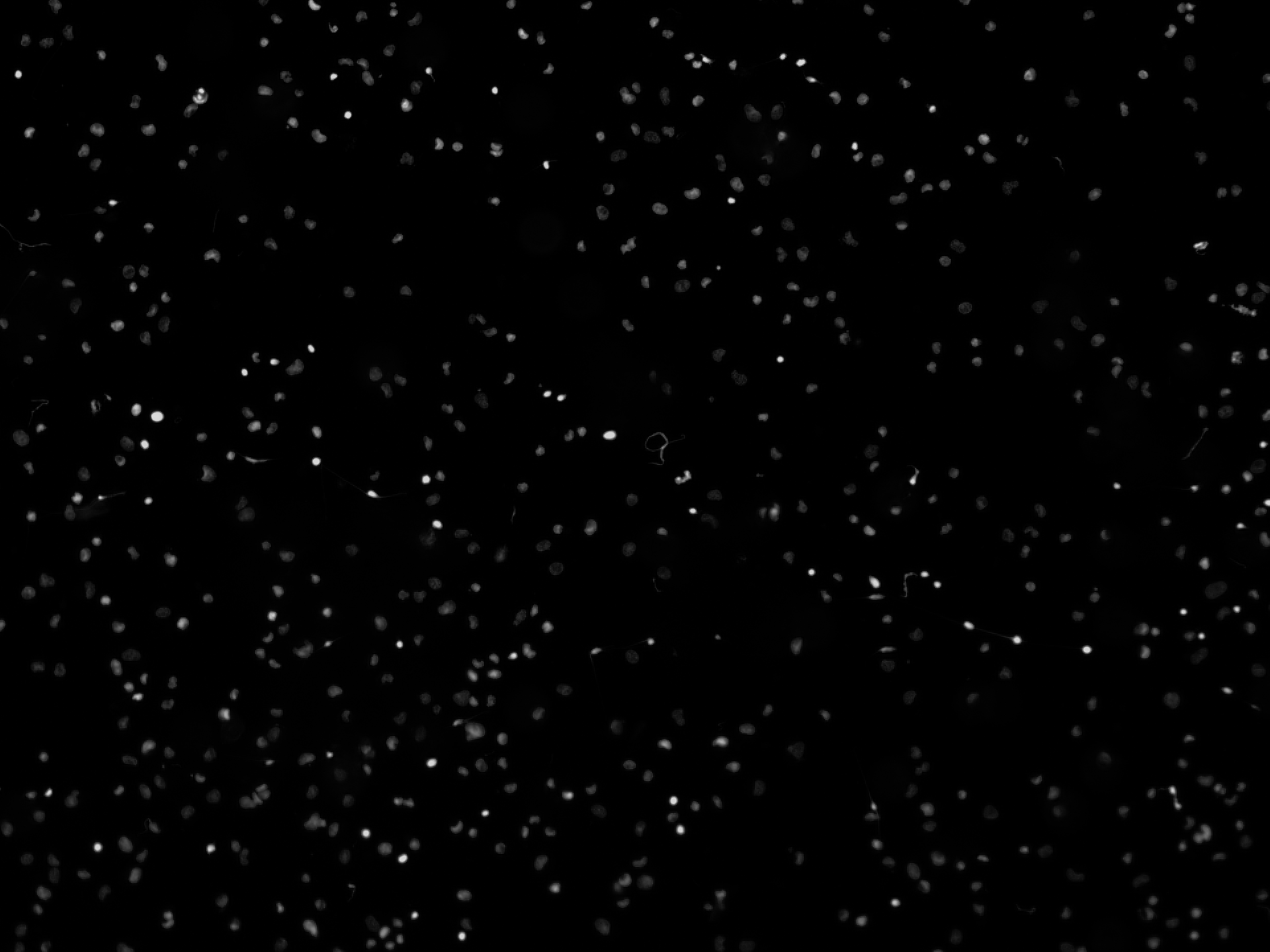

Supplement: Supplementary file 7 — Source data Fig. 1 [file 44321_2025_195_MOESM7_ESM.zip › Figure 1/1G/BTSC73/BTSC73 Mubritinib 100 nM DAPI.tif]

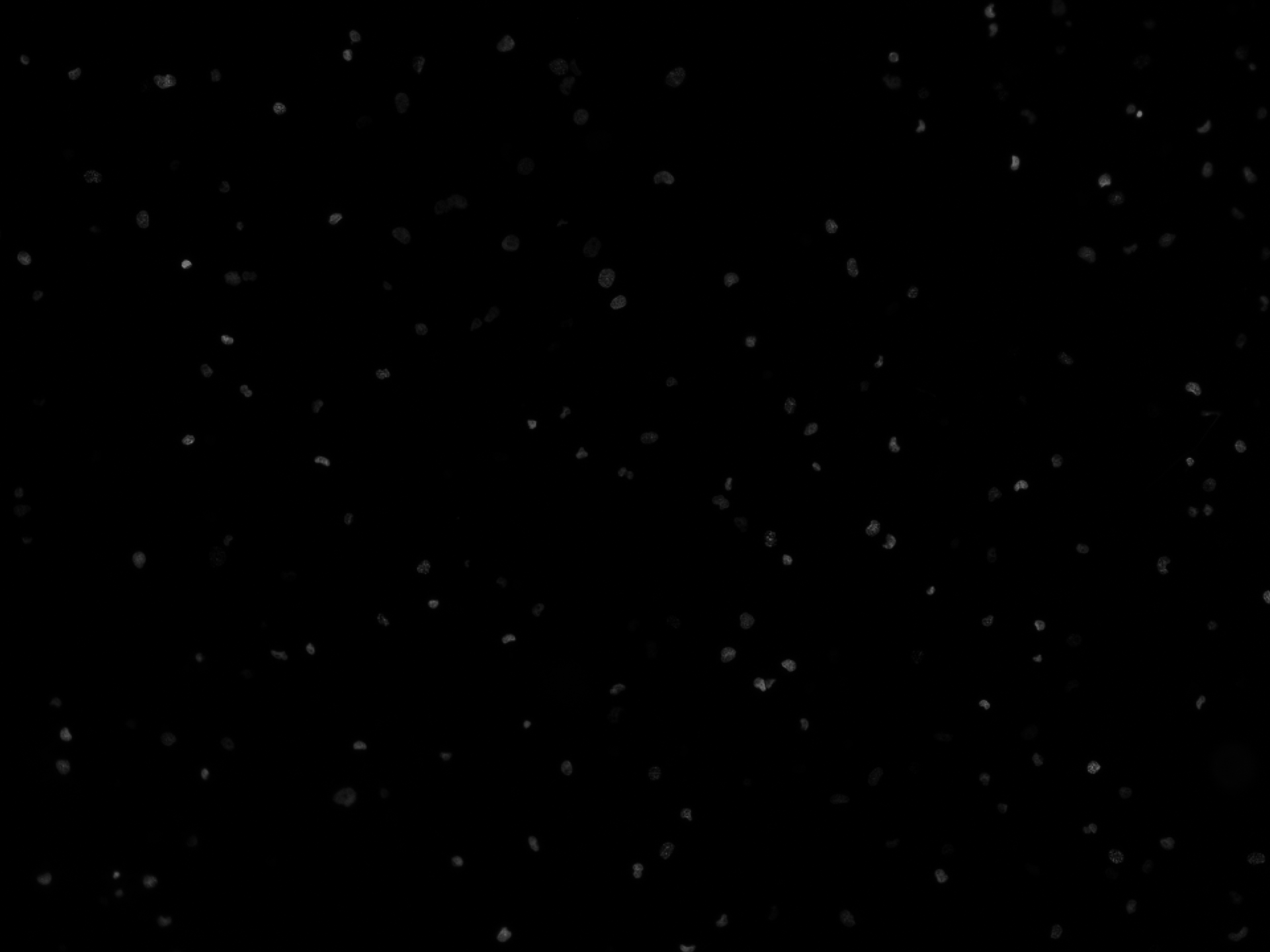

Supplement: Supplementary file 7 — Source data Fig. 1 [file 44321_2025_195_MOESM7_ESM.zip › Figure 1/1G/BTSC73/BTSC73 Mubritinib 500 nM EdU.tif]

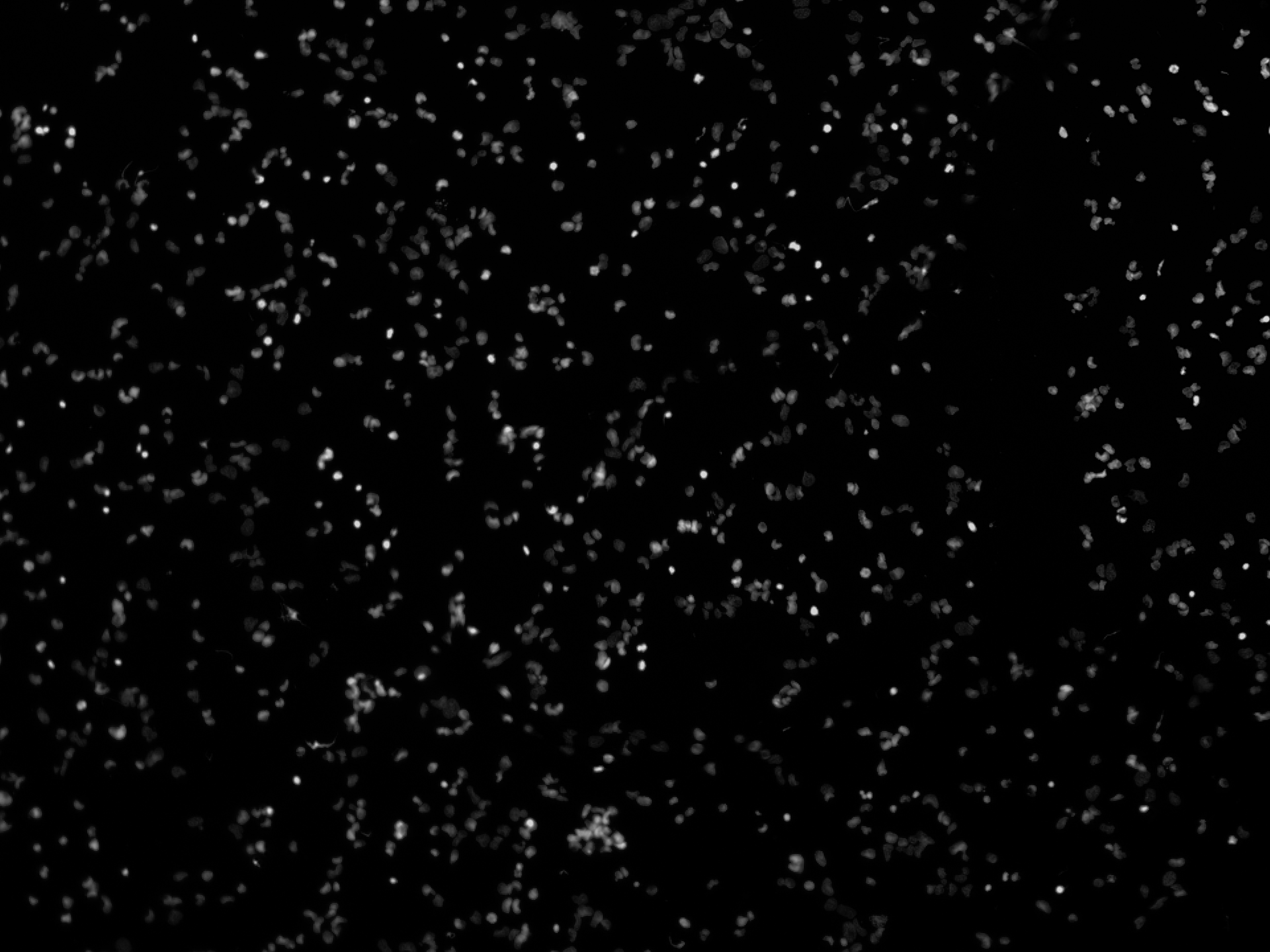

Supplement: Supplementary file 7 — Source data Fig. 1 [file 44321_2025_195_MOESM7_ESM.zip › Figure 1/1G/BTSC73/BTSC73 Control DAPI.tif]

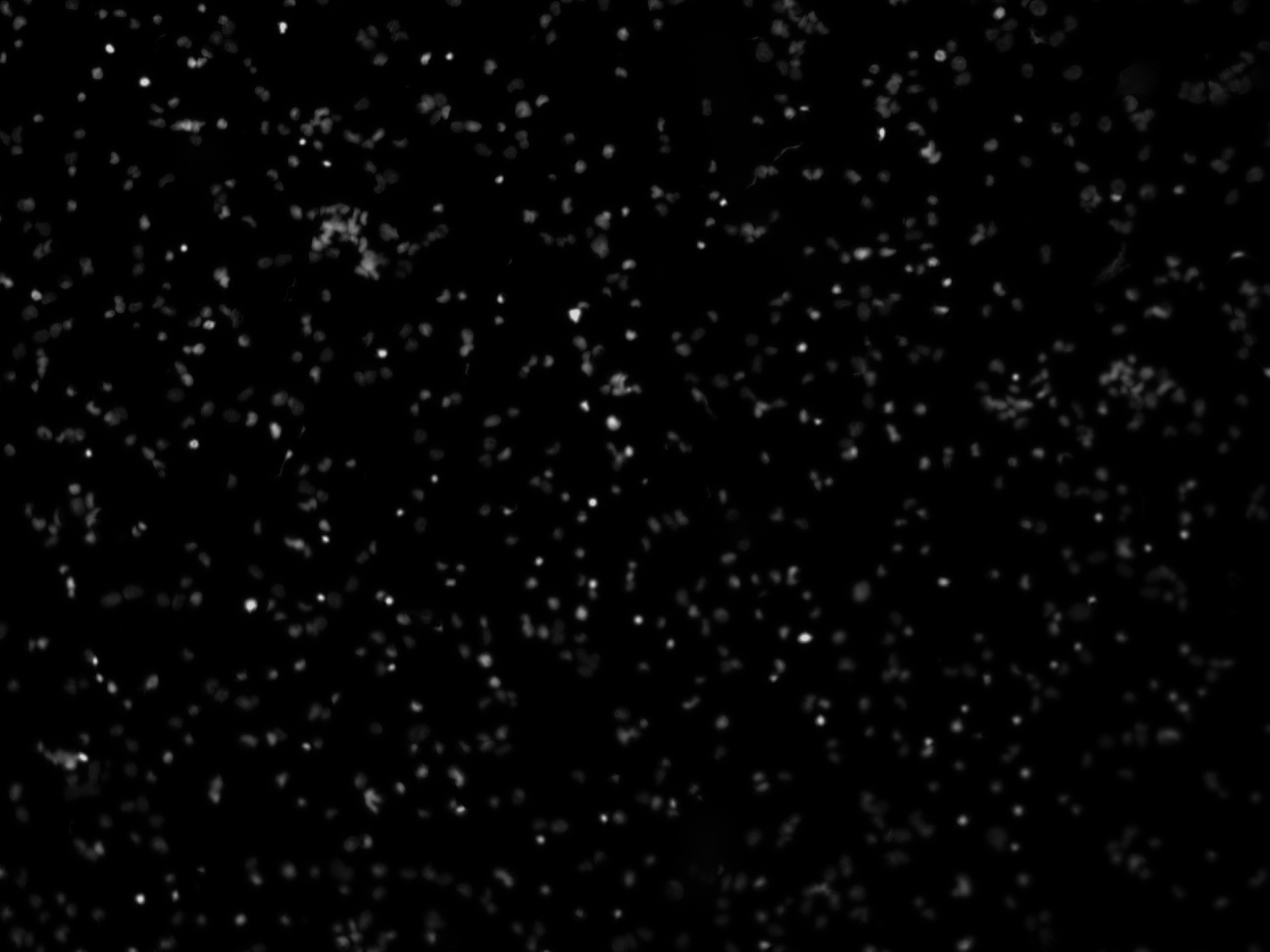

Supplement: Supplementary file 7 — Source data Fig. 1 [file 44321_2025_195_MOESM7_ESM.zip › Figure 1/1G/BTSC73/BTSC73 Mubritinib 20 nM DAPI.tif]

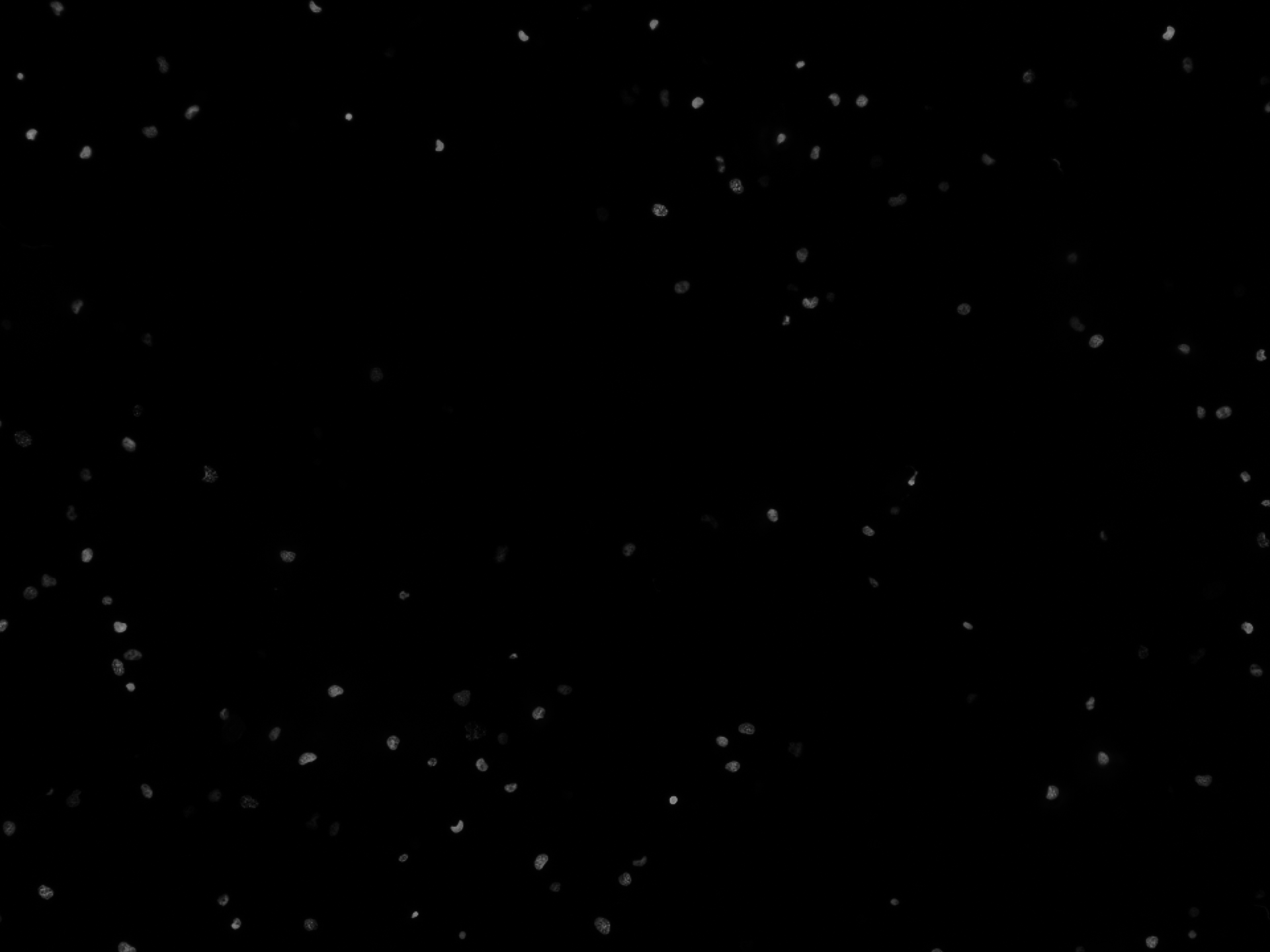

Supplement: Supplementary file 7 — Source data Fig. 1 [file 44321_2025_195_MOESM7_ESM.zip › Figure 1/1G/BTSC73/BTSC73 Mubritinib 100 nM EdU.tif]

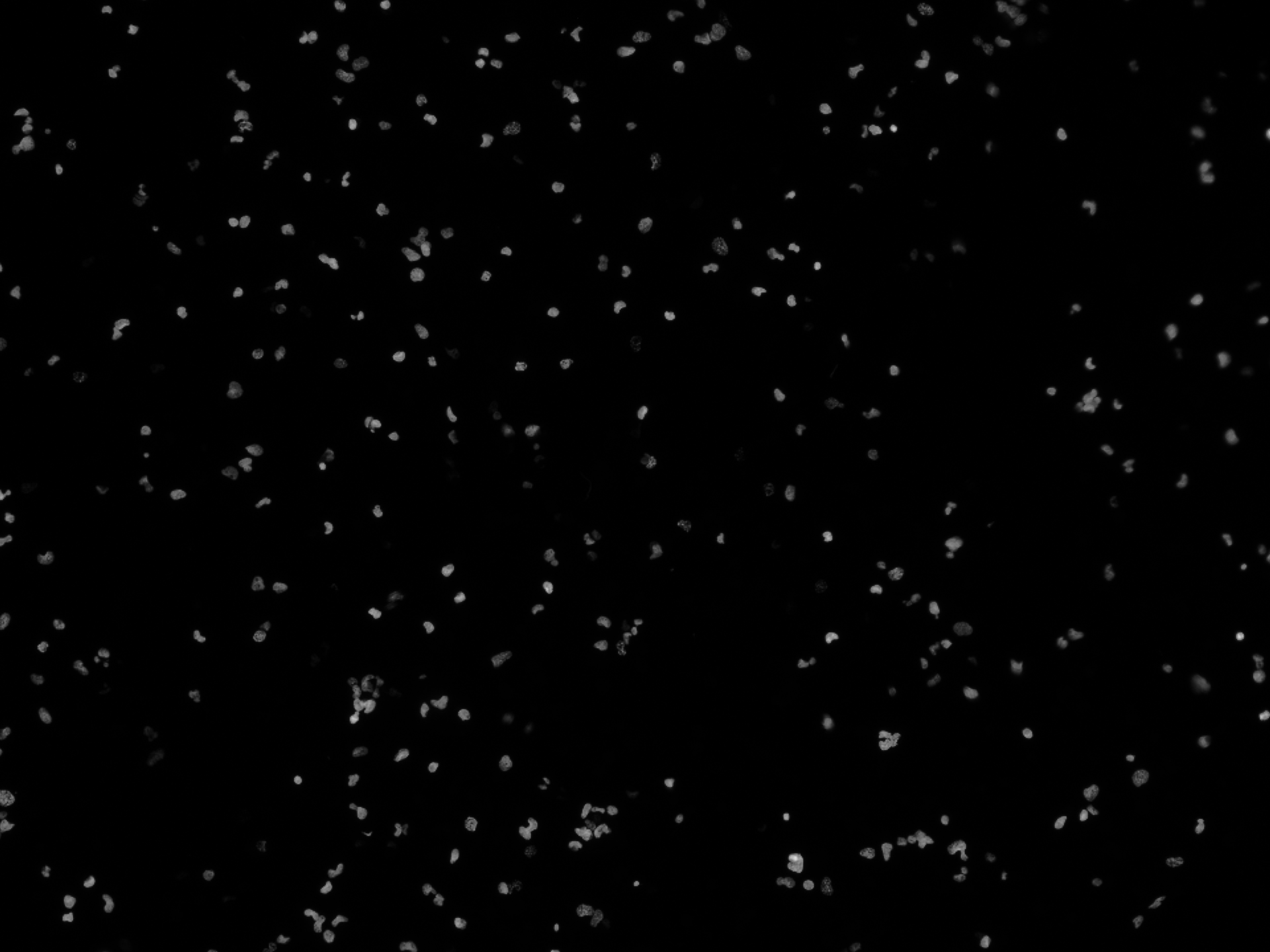

Supplement: Supplementary file 7 — Source data Fig. 1 [file 44321_2025_195_MOESM7_ESM.zip › Figure 1/1G/BTSC73/BTSC73 Control EdU.tif]

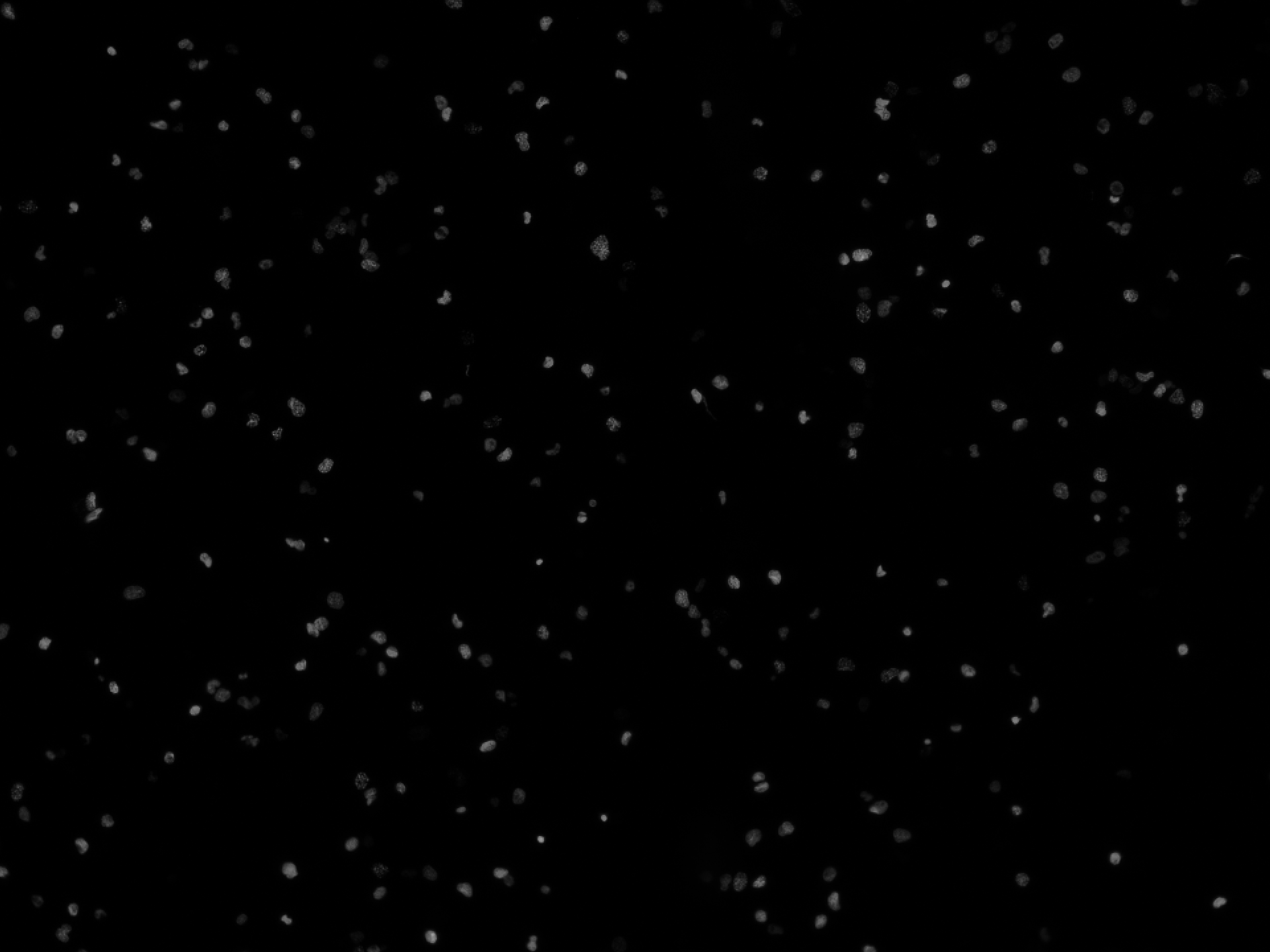

Supplement: Supplementary file 7 — Source data Fig. 1 [file 44321_2025_195_MOESM7_ESM.zip › Figure 1/1G/BTSC73/BTSC73 Mubritinib 20 nM EdU.tif]

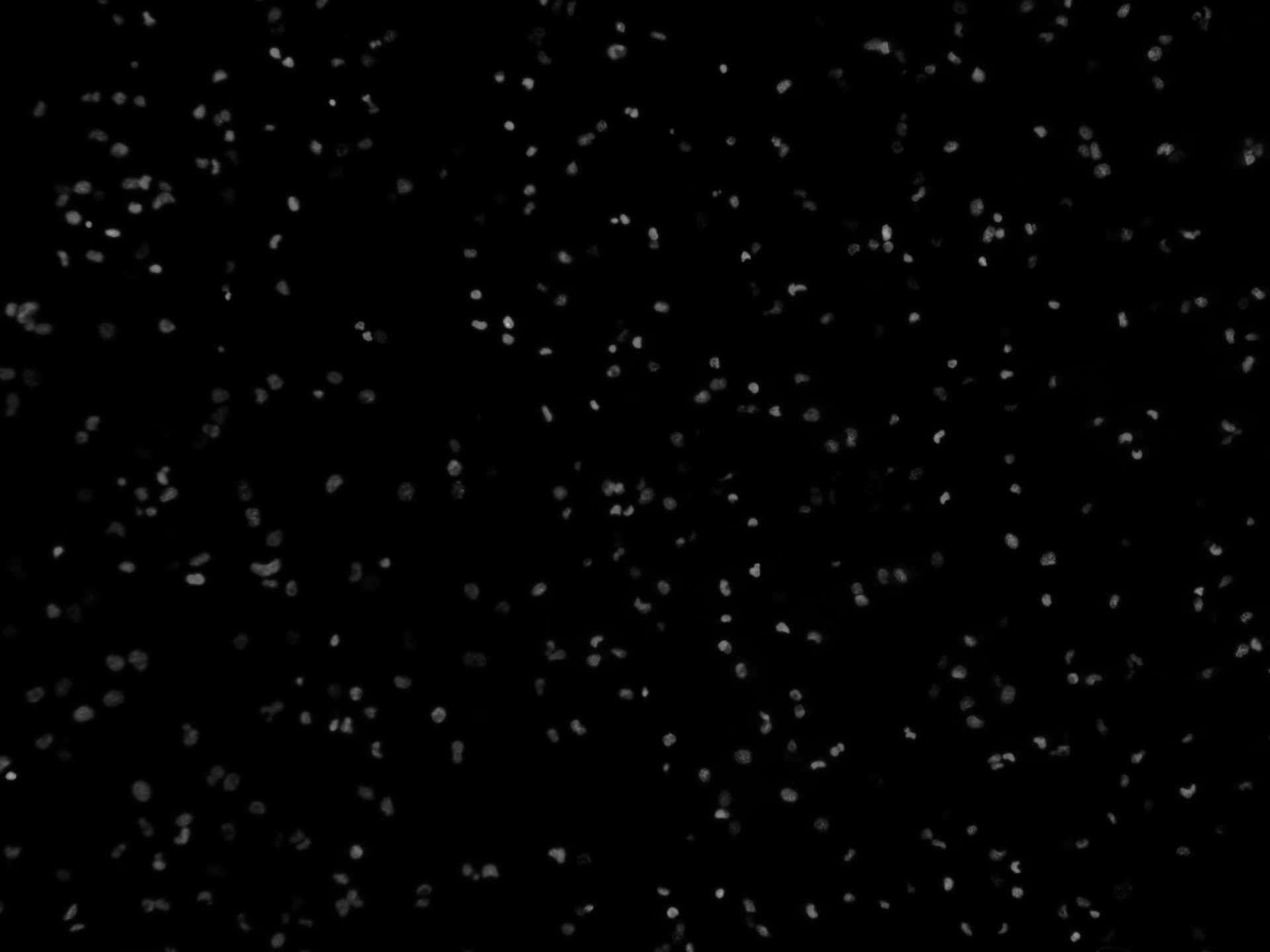

Supplement: Supplementary file 7 — Source data Fig. 1 [file 44321_2025_195_MOESM7_ESM.zip › Figure 1/1G/BTSC147/BTSC147 Mubritinib 20 nM EdU.tif]

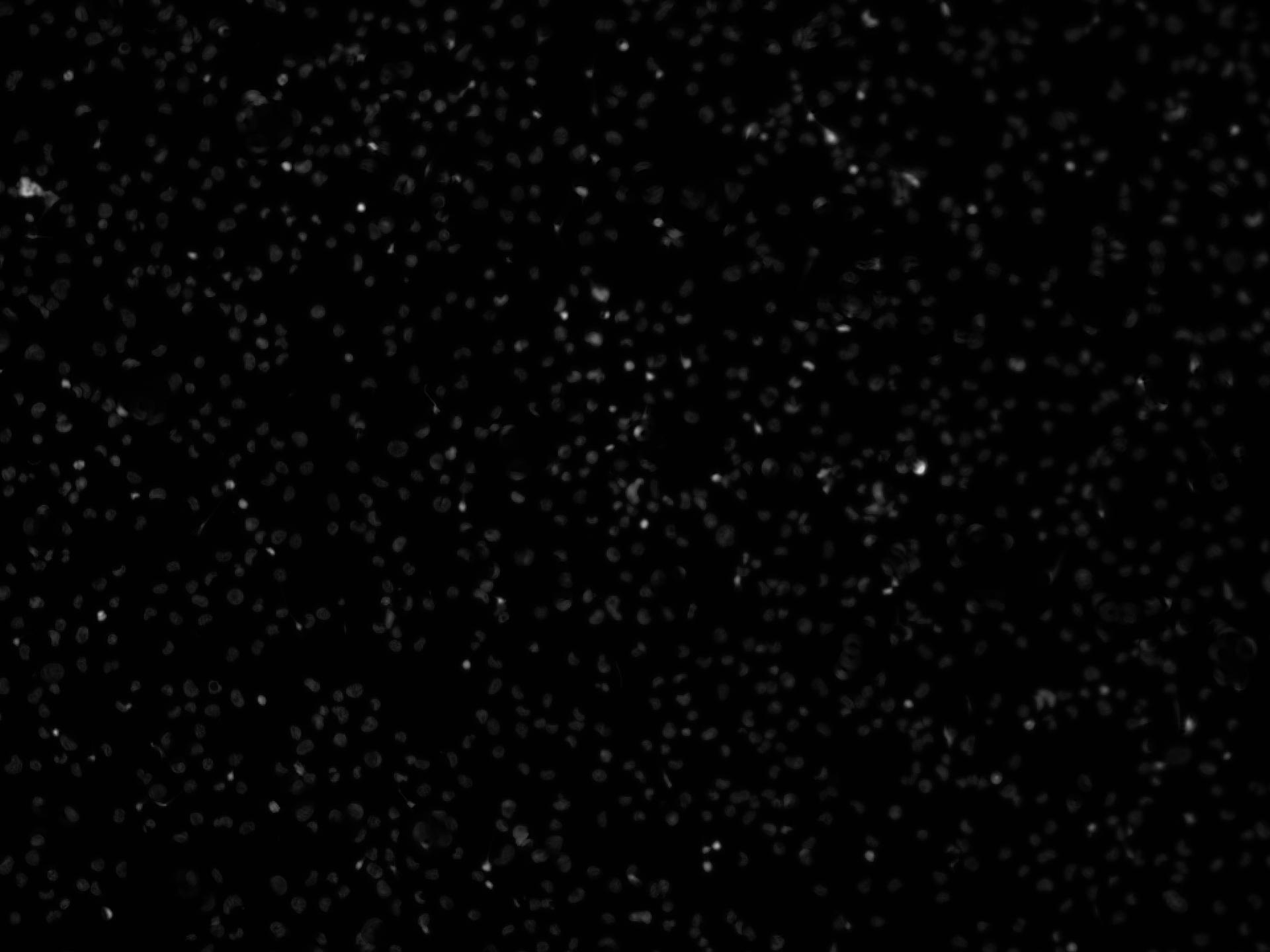

Supplement: Supplementary file 7 — Source data Fig. 1 [file 44321_2025_195_MOESM7_ESM.zip › Figure 1/1G/BTSC147/BTSC147 Mubritinib 100 nM DAPI.tif]

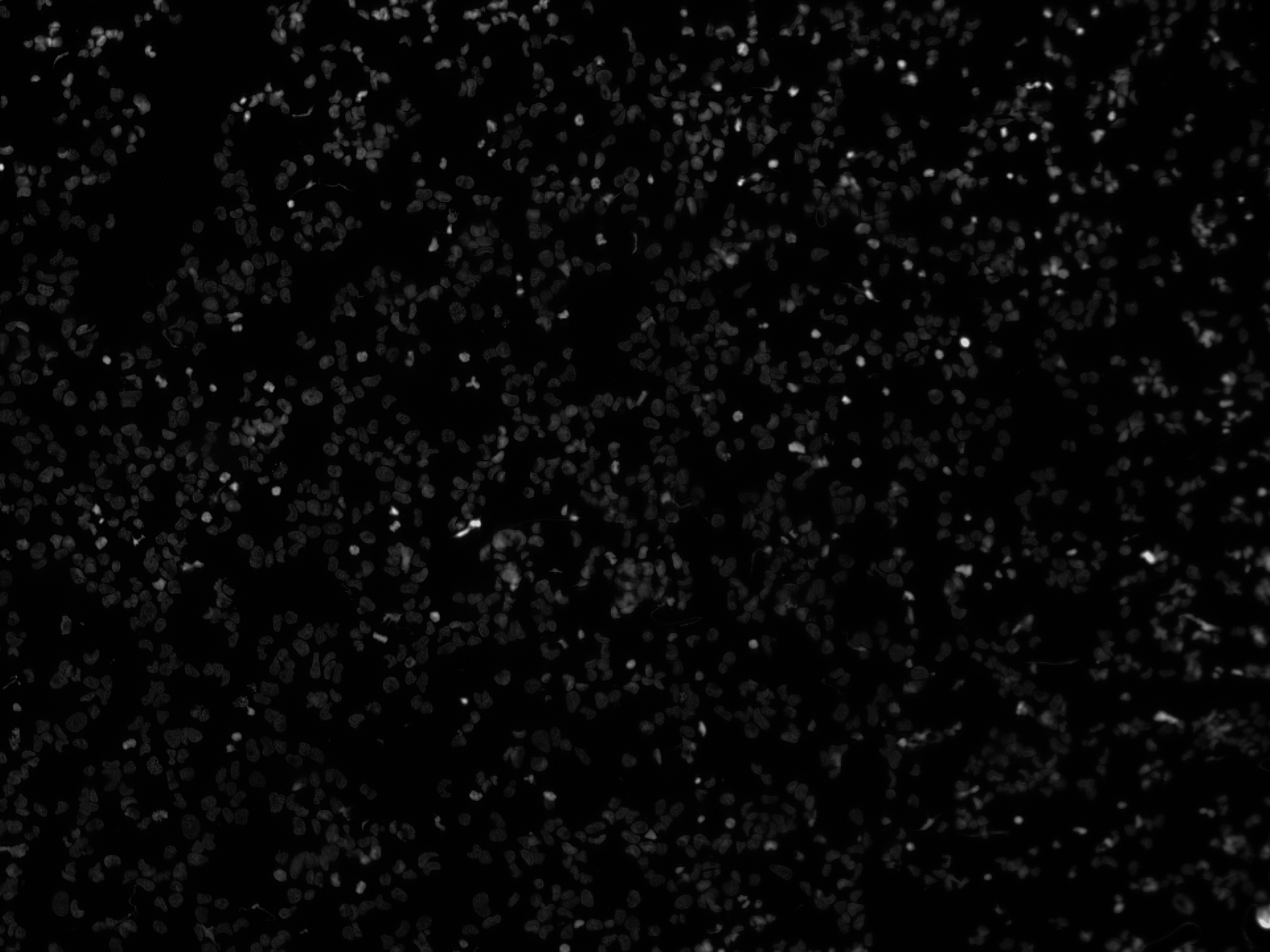

Supplement: Supplementary file 7 — Source data Fig. 1 [file 44321_2025_195_MOESM7_ESM.zip › Figure 1/1G/BTSC147/BTSC147 Control DAPI.tif]

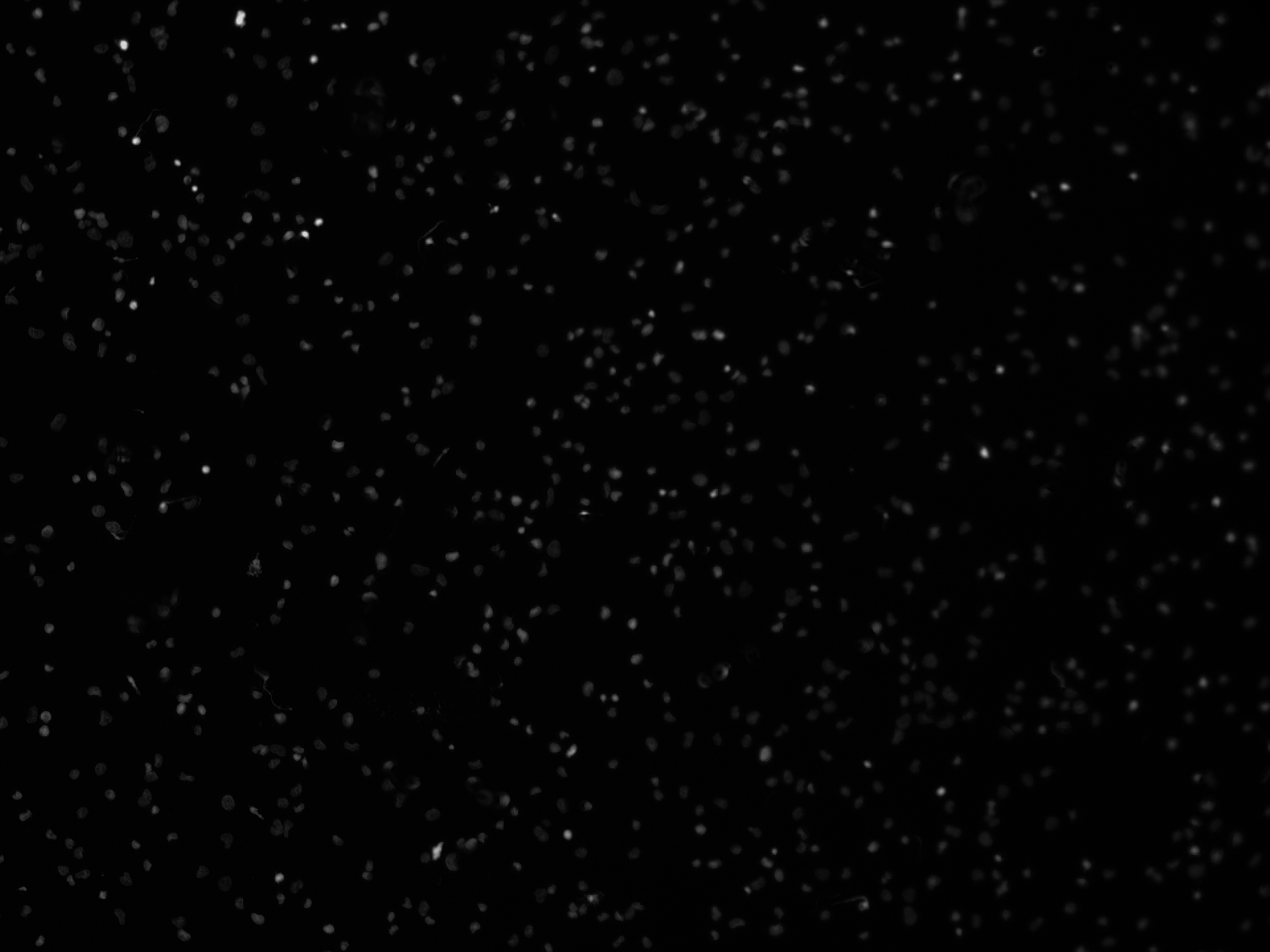

Supplement: Supplementary file 7 — Source data Fig. 1 [file 44321_2025_195_MOESM7_ESM.zip › Figure 1/1G/BTSC147/BTSC147 Mubritinib 500 nM DAPI.tif]

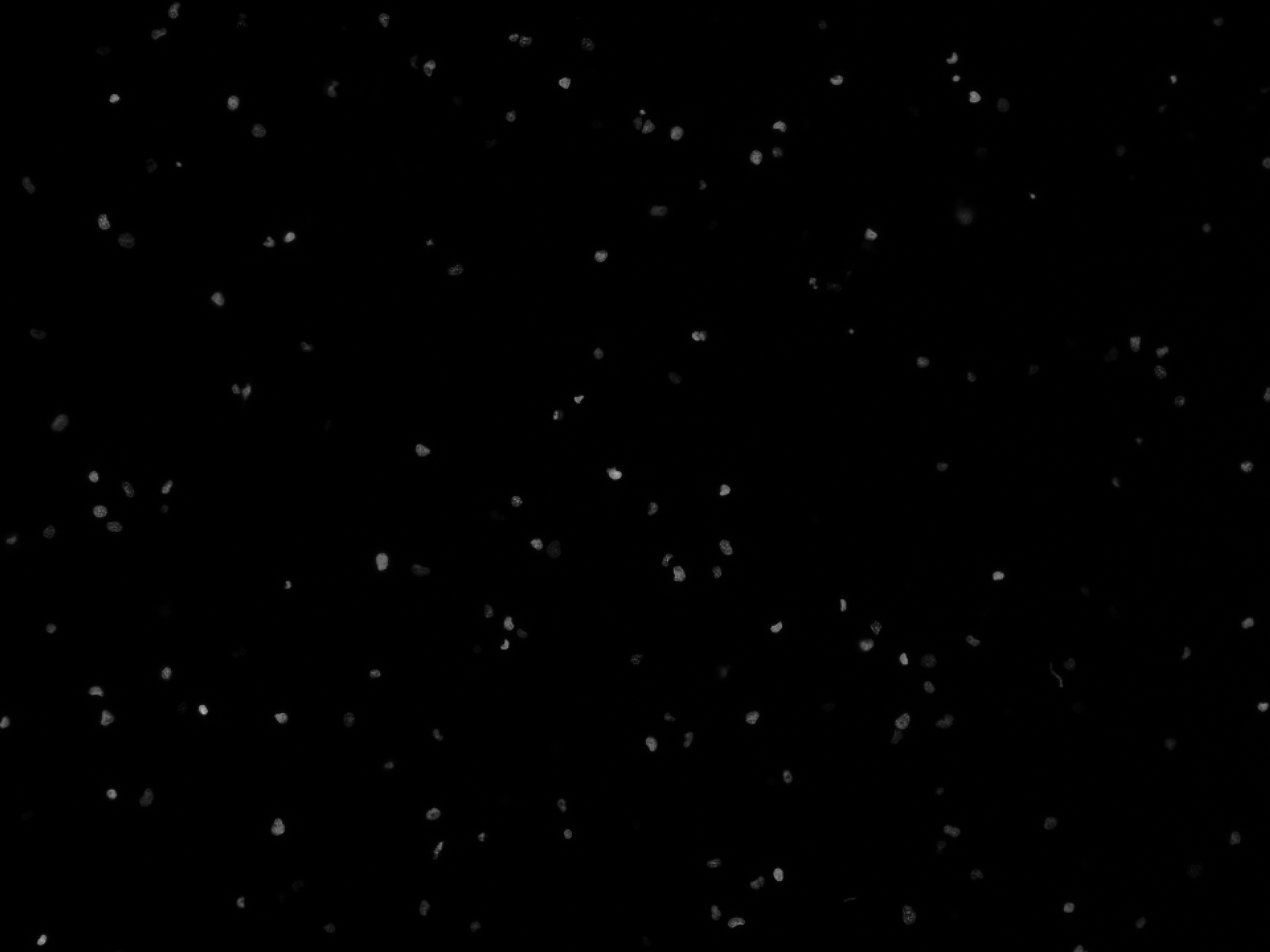

Supplement: Supplementary file 7 — Source data Fig. 1 [file 44321_2025_195_MOESM7_ESM.zip › Figure 1/1G/BTSC147/BTSC147 Mubritinib 500 nM EdU.tif]

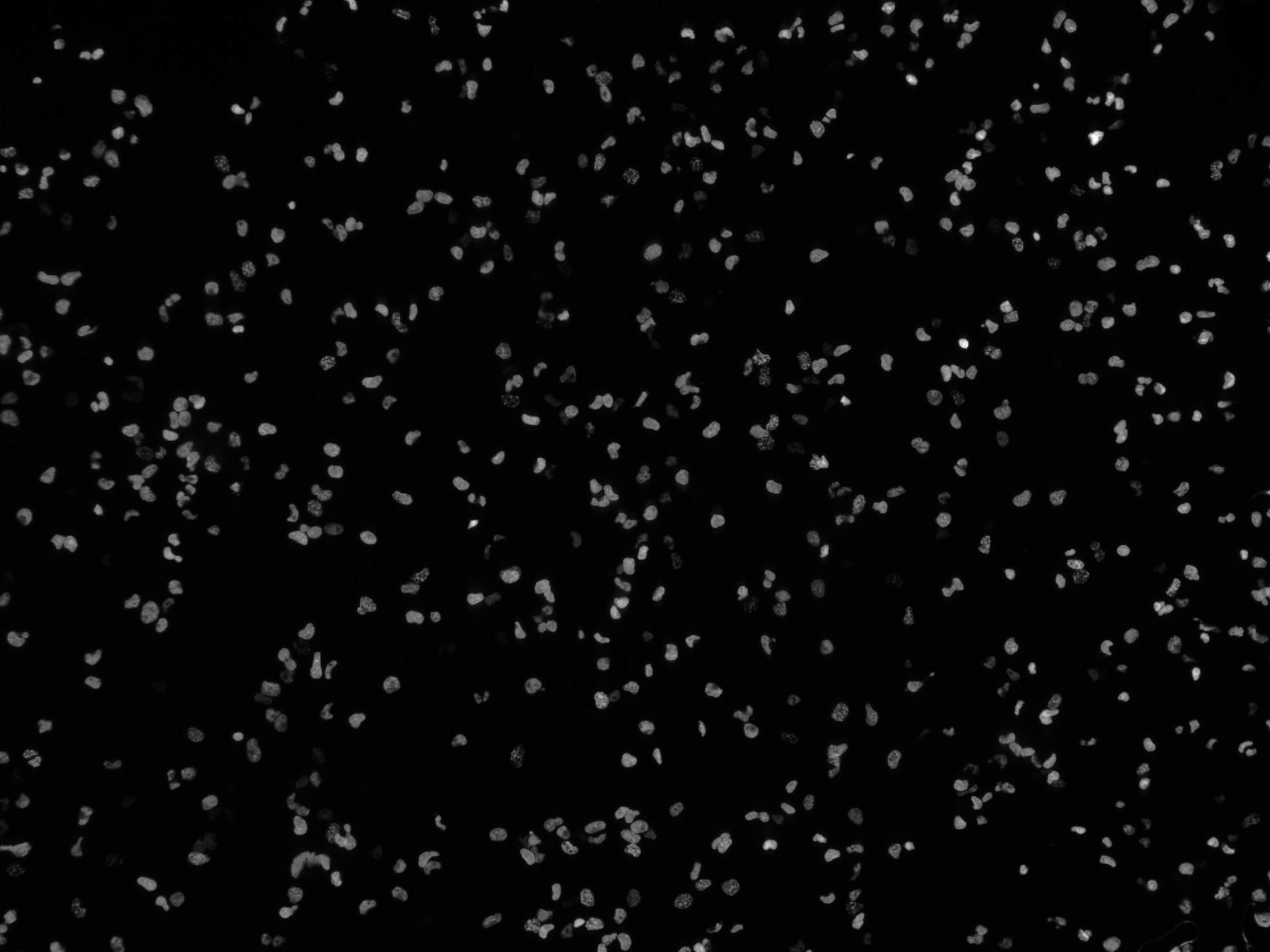

Supplement: Supplementary file 7 — Source data Fig. 1 [file 44321_2025_195_MOESM7_ESM.zip › Figure 1/1G/BTSC147/BTSC147 Control EdU.tif]

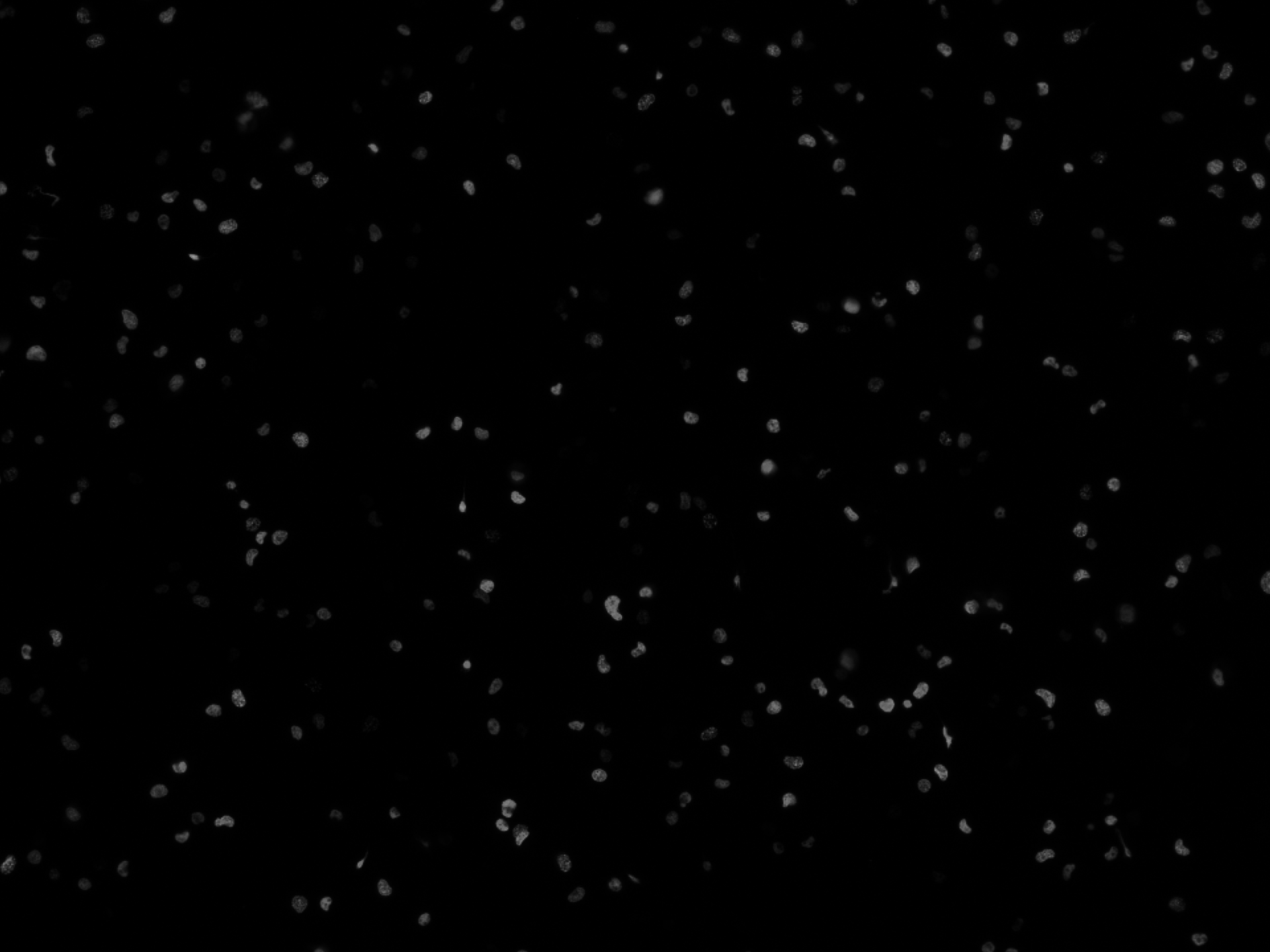

Supplement: Supplementary file 7 — Source data Fig. 1 [file 44321_2025_195_MOESM7_ESM.zip › Figure 1/1G/BTSC147/BTSC147 Mubritinib 100 nM EdU.tif]

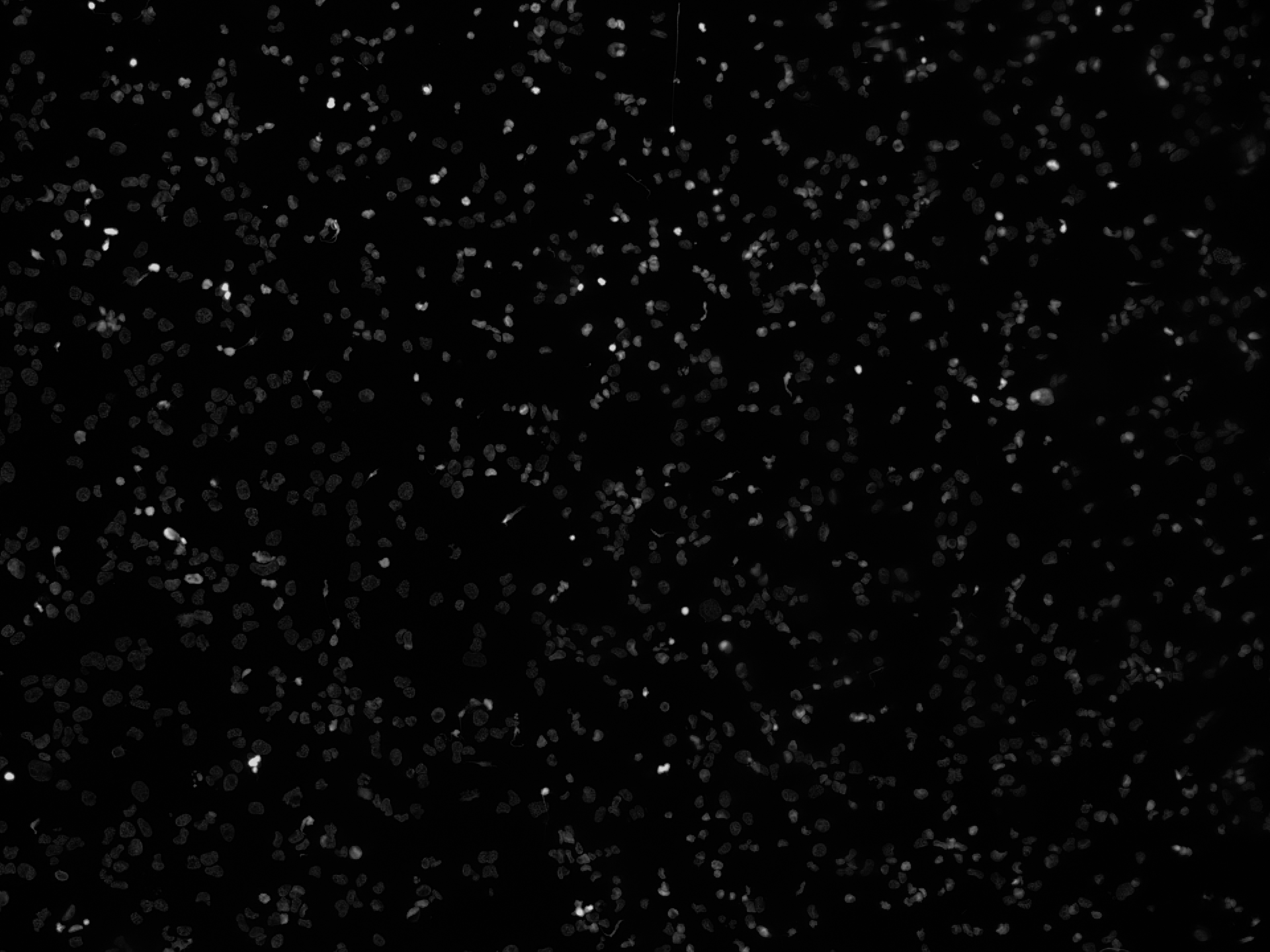

Supplement: Supplementary file 7 — Source data Fig. 1 [file 44321_2025_195_MOESM7_ESM.zip › Figure 1/1G/BTSC147/BTSC147 Mubritinib 20 nM DAPI.tif]

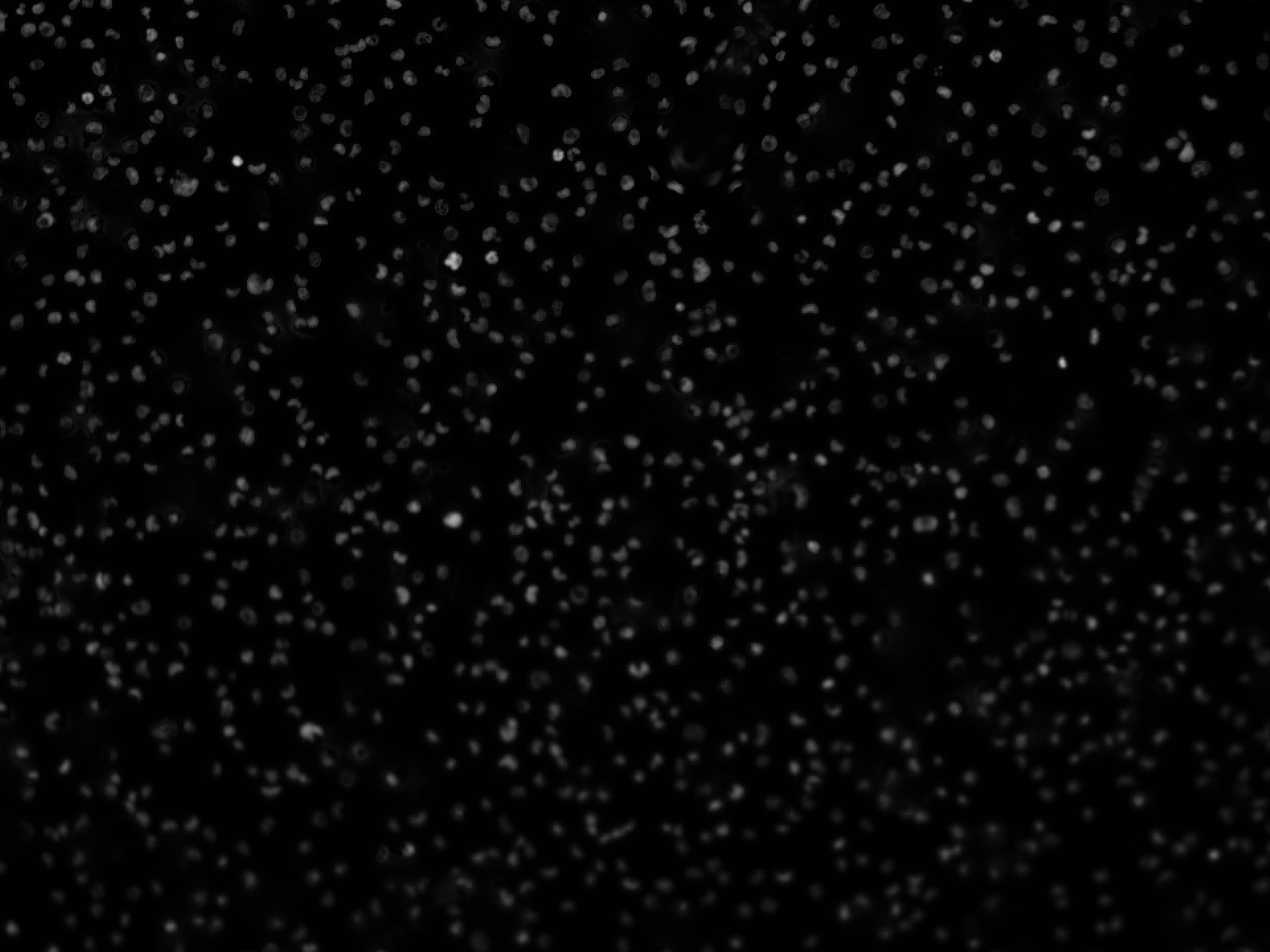

Supplement: Supplementary file 7 — Source data Fig. 1 [file 44321_2025_195_MOESM7_ESM.zip › Figure 1/1G/BTSC53/BTSC53 Mubritinib 20 nM DAPI.tif]

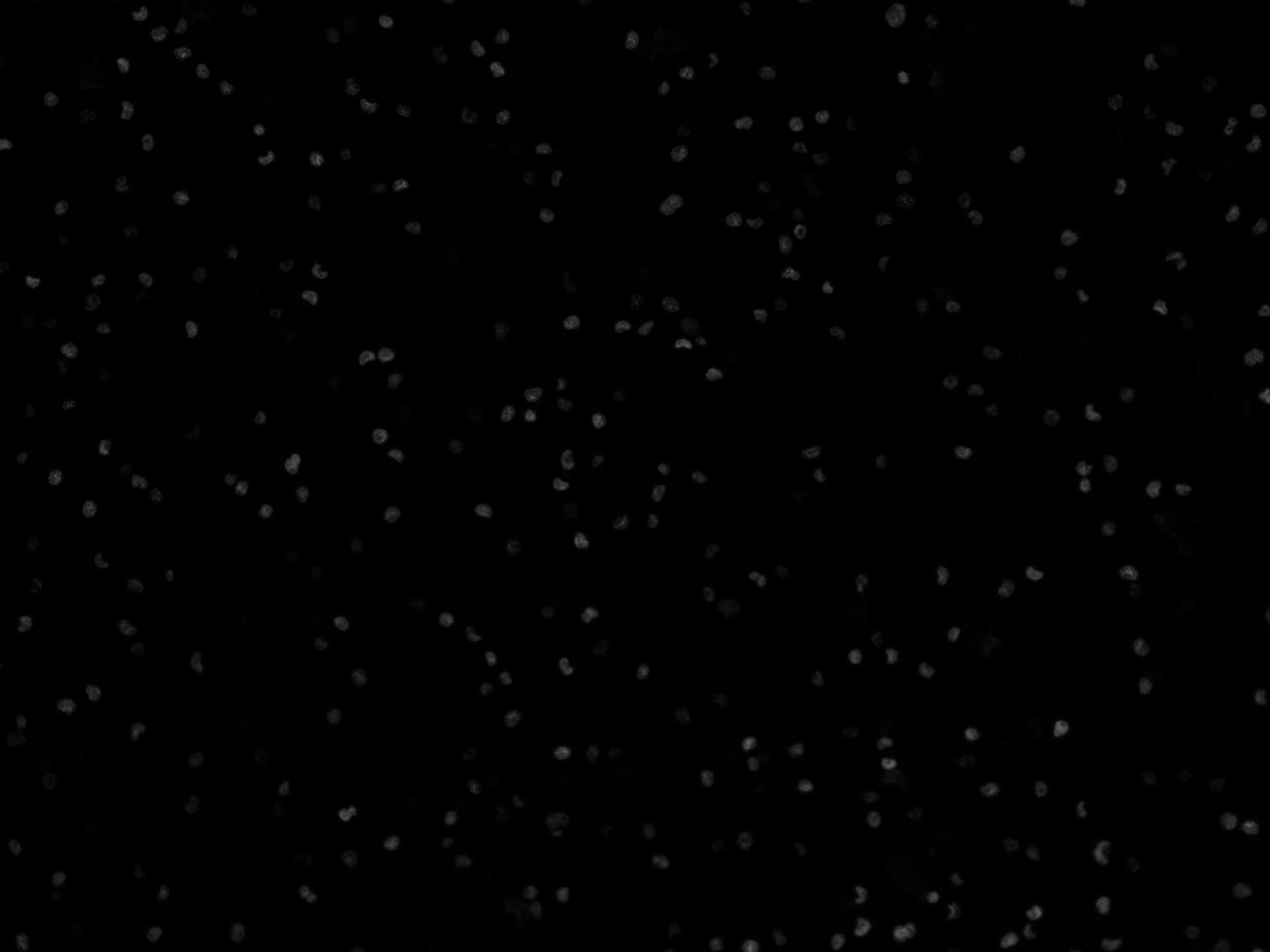

Supplement: Supplementary file 7 — Source data Fig. 1 [file 44321_2025_195_MOESM7_ESM.zip › Figure 1/1G/BTSC53/BTSC53 Mubritinib 100 nM EdU.tif]

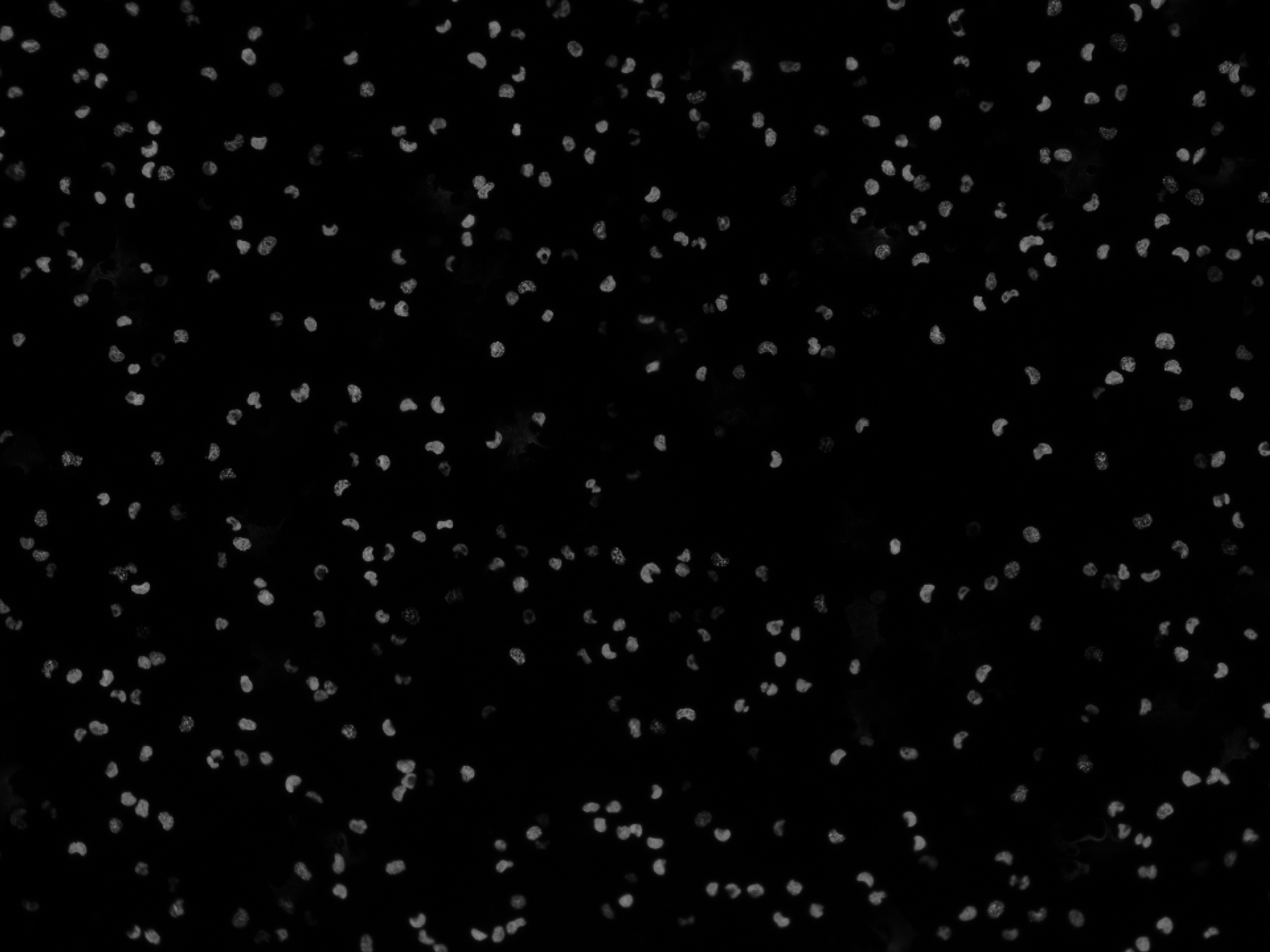

Supplement: Supplementary file 7 — Source data Fig. 1 [file 44321_2025_195_MOESM7_ESM.zip › Figure 1/1G/BTSC53/BTSC53 Control EdU.tif]

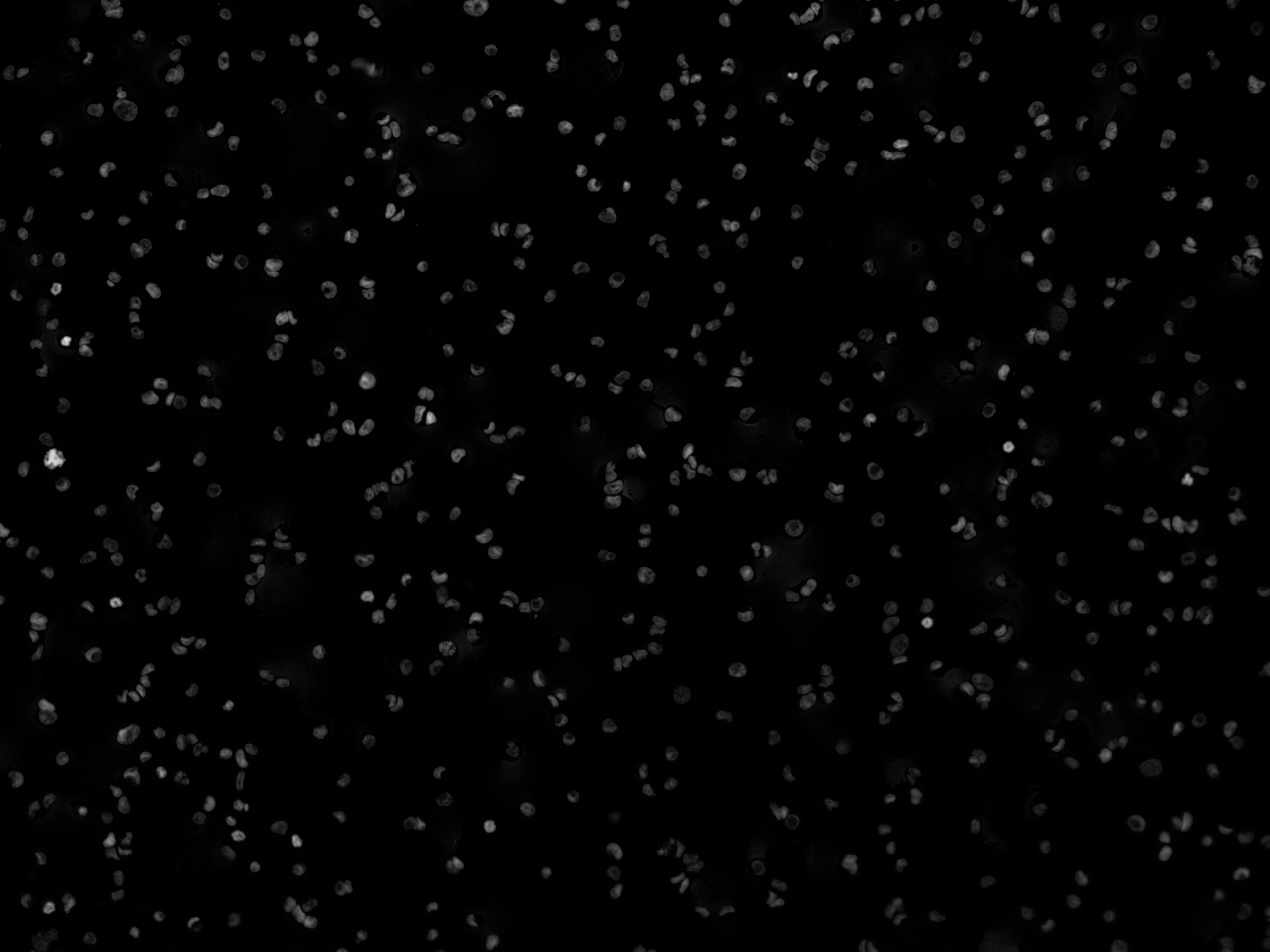

Supplement: Supplementary file 7 — Source data Fig. 1 [file 44321_2025_195_MOESM7_ESM.zip › Figure 1/1G/BTSC53/BTSC53 Mubritinib 500 nM DAPI.tif]

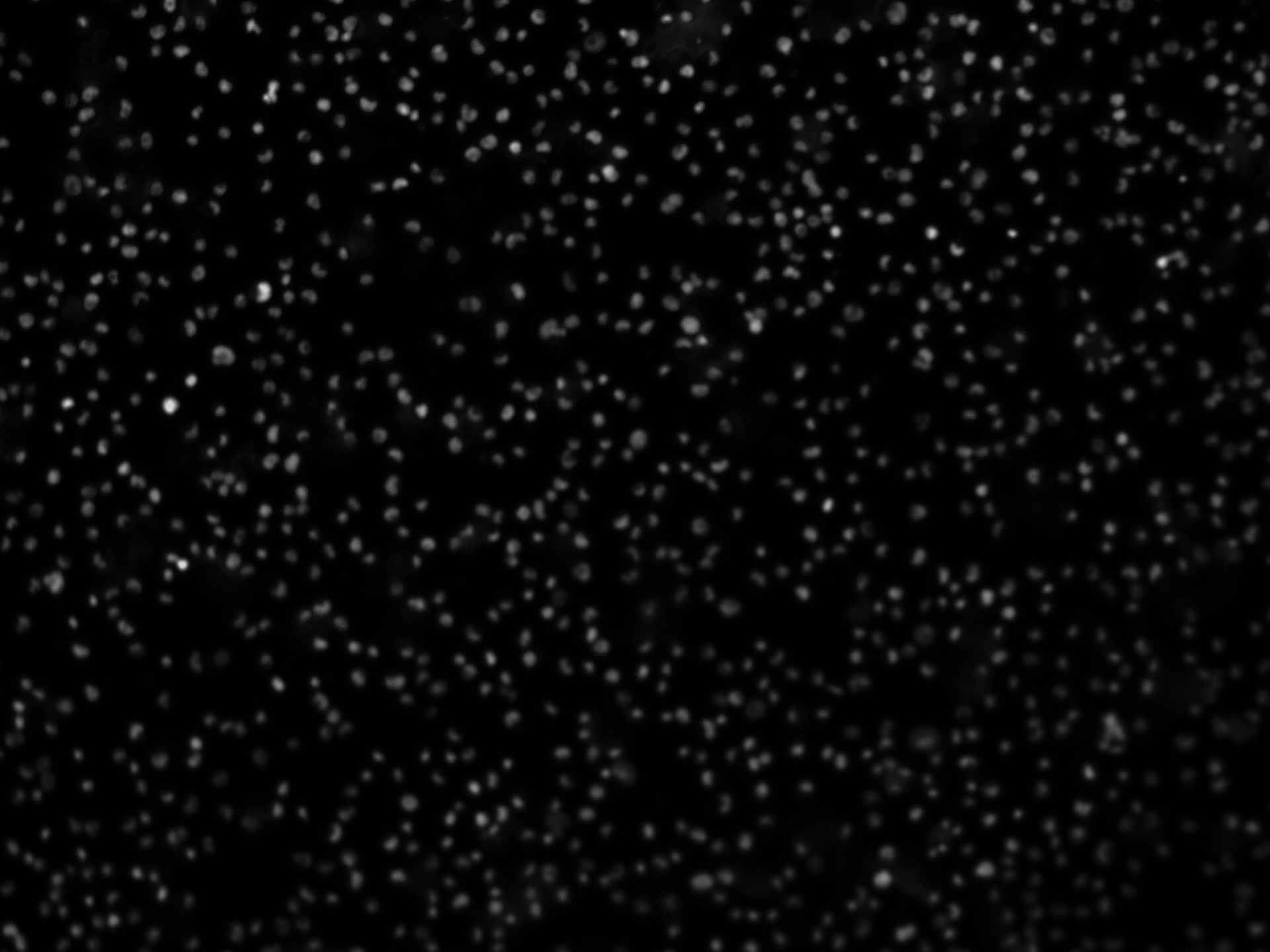

Supplement: Supplementary file 7 — Source data Fig. 1 [file 44321_2025_195_MOESM7_ESM.zip › Figure 1/1G/BTSC53/BTSC53 Mubritinib 100 nM DAPI.tif]

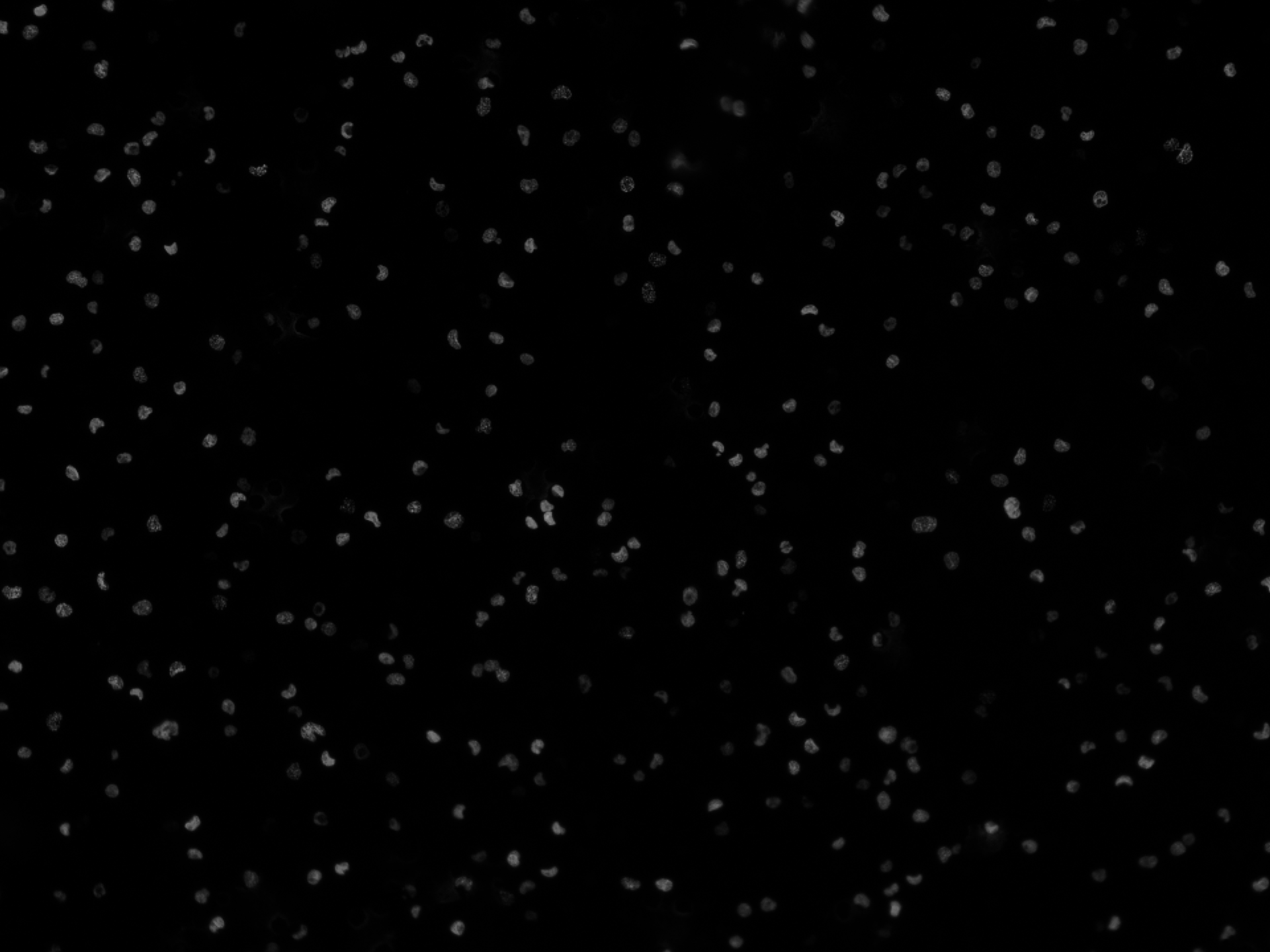

Supplement: Supplementary file 7 — Source data Fig. 1 [file 44321_2025_195_MOESM7_ESM.zip › Figure 1/1G/BTSC53/BTSC53 Mubritinib 20 nM EdU.tif]

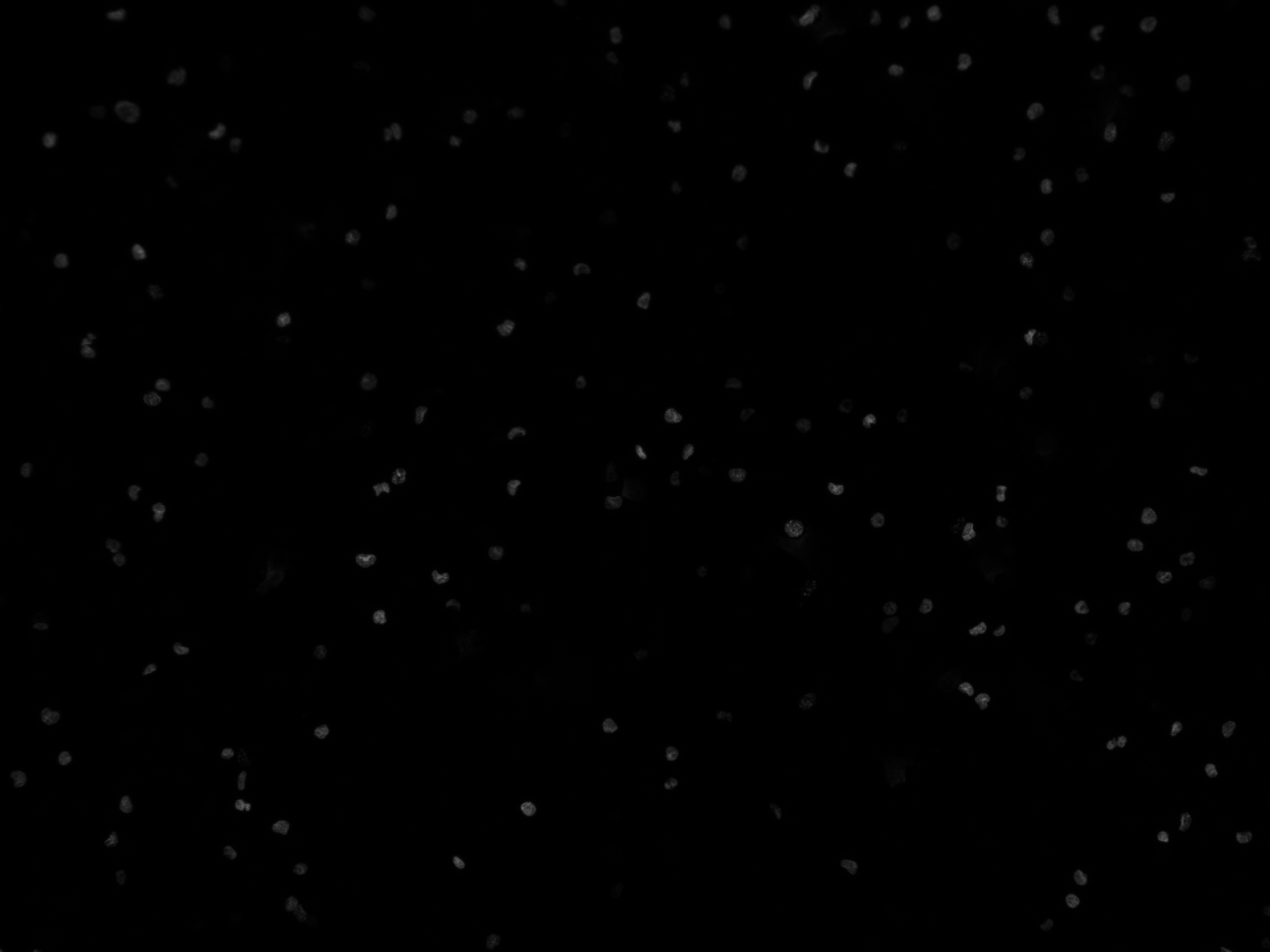

Supplement: Supplementary file 7 — Source data Fig. 1 [file 44321_2025_195_MOESM7_ESM.zip › Figure 1/1G/BTSC53/BTSC53 Mubritinib 500 nM EdU.tif]

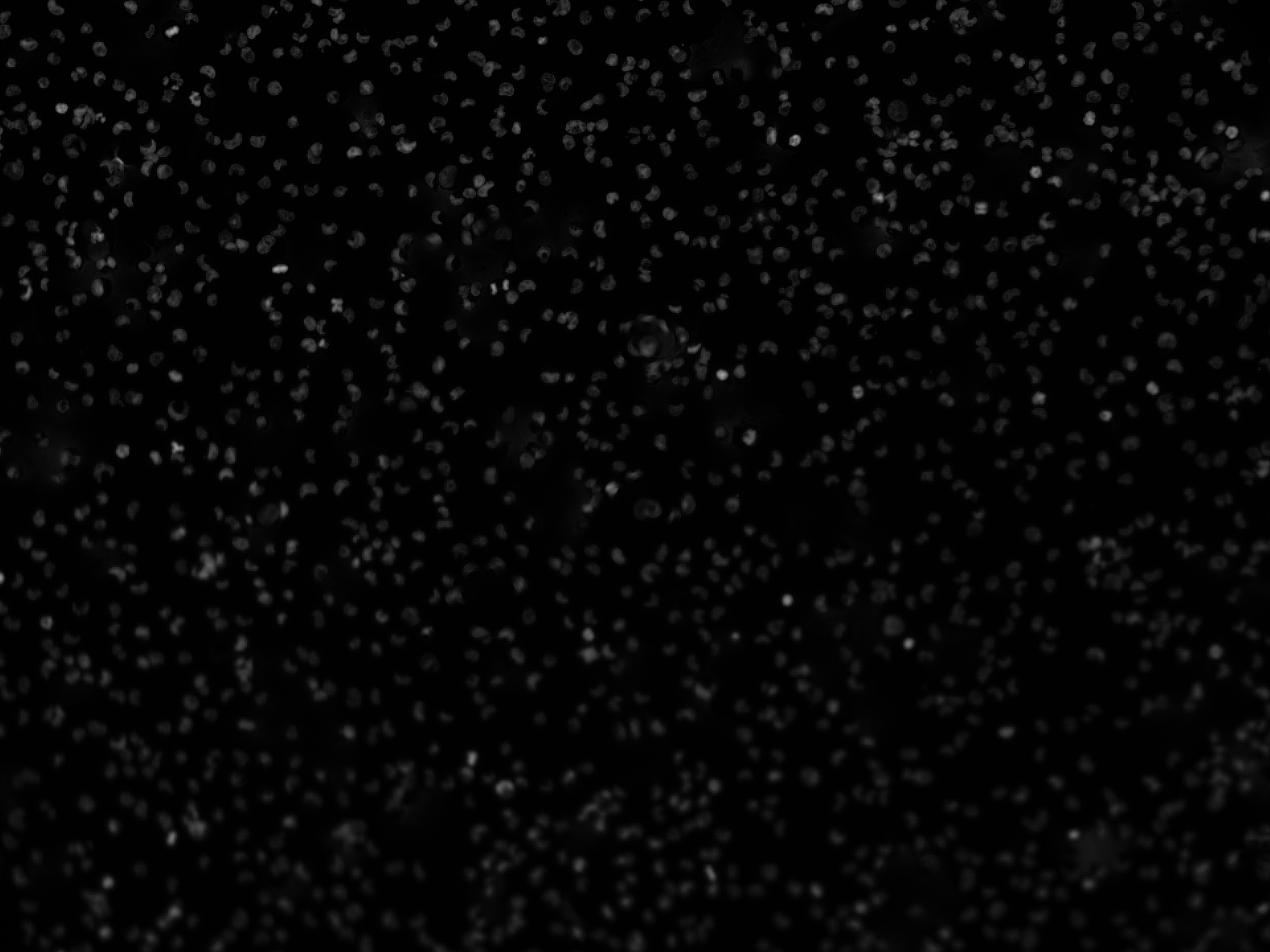

Supplement: Supplementary file 7 — Source data Fig. 1 [file 44321_2025_195_MOESM7_ESM.zip › Figure 1/1G/BTSC53/BTSC53 Control DAPI.tif]

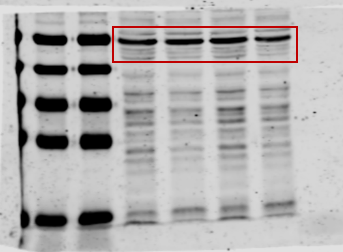

Supplement: Supplementary file 8 — Source data Fig. 2 [file 44321_2025_195_MOESM8_ESM.zip › Figure 2/2G/Figure 2G Tubulin 72h.png]

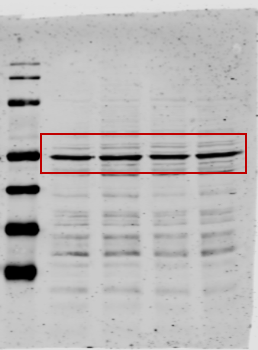

Supplement: Supplementary file 8 — Source data Fig. 2 [file 44321_2025_195_MOESM8_ESM.zip › Figure 2/2G/Figure 2G Tubulin 48h.png]

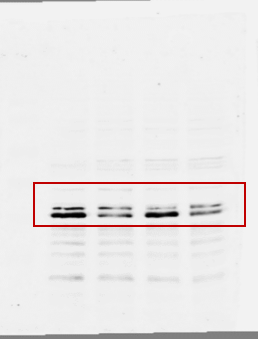

Supplement: Supplementary file 8 — Source data Fig. 2 [file 44321_2025_195_MOESM8_ESM.zip › Figure 2/2G/Figure 2G CyclinD1 48h.png]

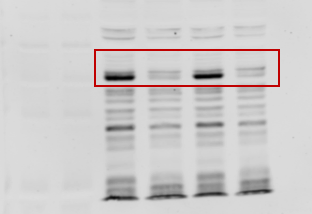

Supplement: Supplementary file 8 — Source data Fig. 2 [file 44321_2025_195_MOESM8_ESM.zip › Figure 2/2G/Figure 2G CyclinD1 72h.png]

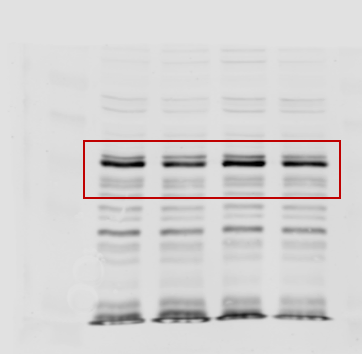

Supplement: Supplementary file 8 — Source data Fig. 2 [file 44321_2025_195_MOESM8_ESM.zip › Figure 2/2G/Figure 2G CyclinD1 24h.png]

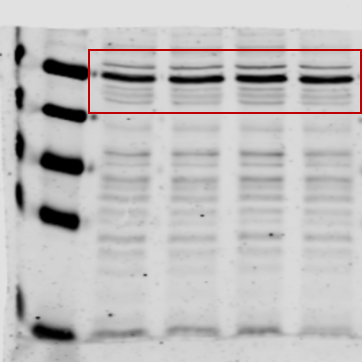

Supplement: Supplementary file 8 — Source data Fig. 2 [file 44321_2025_195_MOESM8_ESM.zip › Figure 2/2G/Figure 2G Tubulin 24h.png]

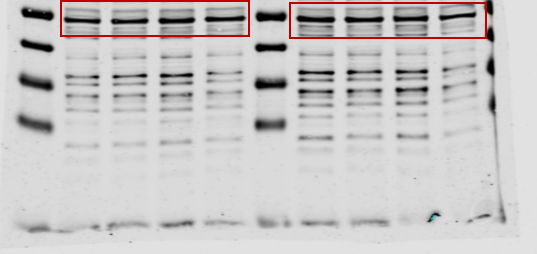

Supplement: Supplementary file 8 — Source data Fig. 2 [file 44321_2025_195_MOESM8_ESM.zip › Figure 2/2I/Figure 2I Tubulin 24h and 48h.png]

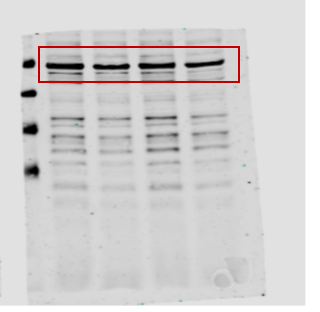

Supplement: Supplementary file 8 — Source data Fig. 2 [file 44321_2025_195_MOESM8_ESM.zip › Figure 2/2I/Figure 2I Tubulin 72h.png]

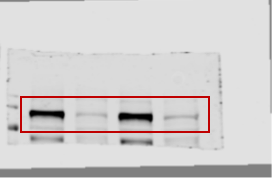

Supplement: Supplementary file 8 — Source data Fig. 2 [file 44321_2025_195_MOESM8_ESM.zip › Figure 2/2I/Figure 2I pRb 72h.png]

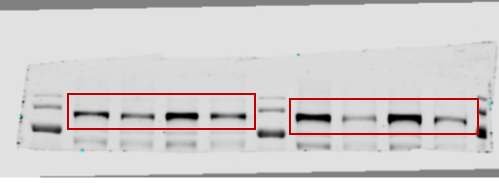

Supplement: Supplementary file 8 — Source data Fig. 2 [file 44321_2025_195_MOESM8_ESM.zip › Figure 2/2I/Figure 2I pRb 24h and 48h.png]

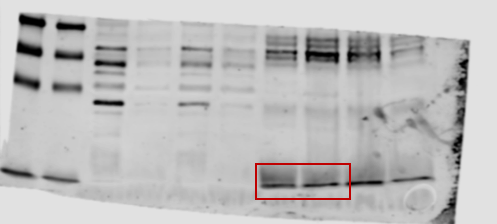

Supplement: Supplementary file 8 — Source data Fig. 2 [file 44321_2025_195_MOESM8_ESM.zip › Figure 2/2H/Figure 2H H2AX BTSC53.png]

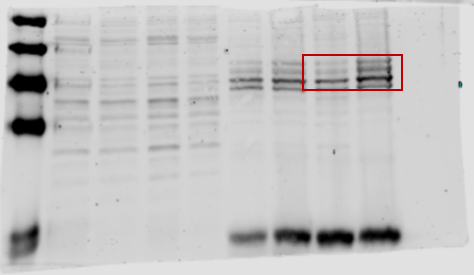

Supplement: Supplementary file 8 — Source data Fig. 2 [file 44321_2025_195_MOESM8_ESM.zip › Figure 2/2H/Figure 2H p27kip1 BTSC73.png]

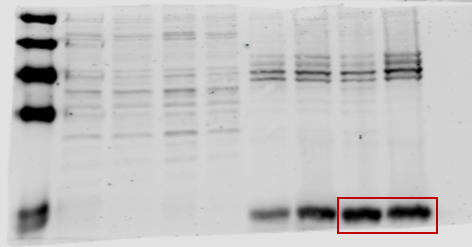

Supplement: Supplementary file 8 — Source data Fig. 2 [file 44321_2025_195_MOESM8_ESM.zip › Figure 2/2H/Figure 2H H2AX BTSC73.png]

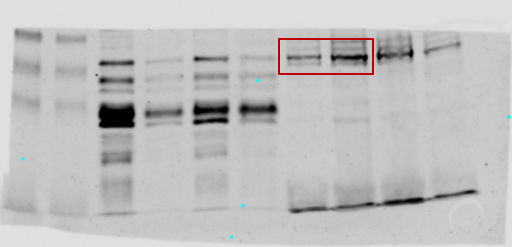

Supplement: Supplementary file 8 — Source data Fig. 2 [file 44321_2025_195_MOESM8_ESM.zip › Figure 2/2H/Figure 2H p27kip1 BTSC53.png]

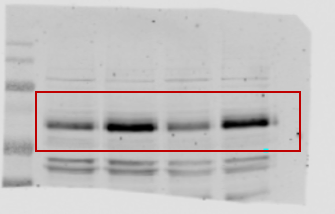

Supplement: Supplementary file 8 — Source data Fig. 2 [file 44321_2025_195_MOESM8_ESM.zip › Figure 2/2F/Figure 2F pAMPK.png]

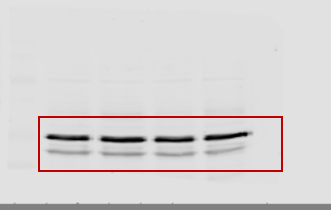

Supplement: Supplementary file 8 — Source data Fig. 2 [file 44321_2025_195_MOESM8_ESM.zip › Figure 2/2F/Figure 2F Tubulin.png]

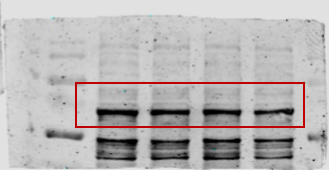

Supplement: Supplementary file 8 — Source data Fig. 2 [file 44321_2025_195_MOESM8_ESM.zip › Figure 2/2F/Figure 2F total AMPK.png]

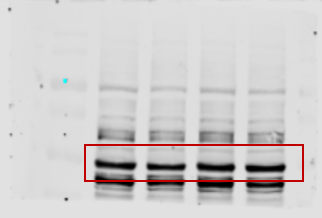

Supplement: Supplementary file 8 — Source data Fig. 2 [file 44321_2025_195_MOESM8_ESM.zip › Figure 2/2F/Figure 2F Tubulin 2.png]

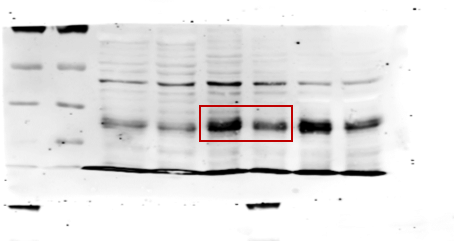

Supplement: Supplementary file 9 — Source data Fig. 3 [file 44321_2025_195_MOESM9_ESM.zip › Figure 3/3K/Figure 3K SOX2.png]

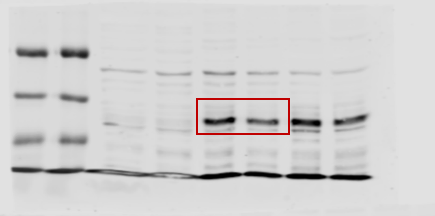

Supplement: Supplementary file 9 — Source data Fig. 3 [file 44321_2025_195_MOESM9_ESM.zip › Figure 3/3K/Figure 3K Olig2.png]

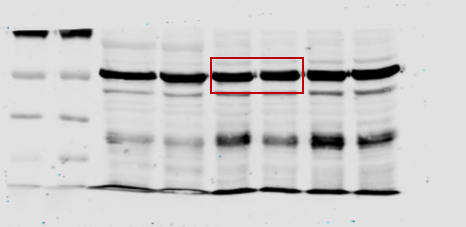

Supplement: Supplementary file 9 — Source data Fig. 3 [file 44321_2025_195_MOESM9_ESM.zip › Figure 3/3K/Figure 3K Tubulin.png]

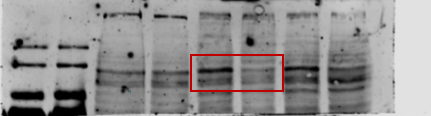

Supplement: Supplementary file 9 — Source data Fig. 3 [file 44321_2025_195_MOESM9_ESM.zip › Figure 3/3K/Figure 3K Cleaved Notch1.png]

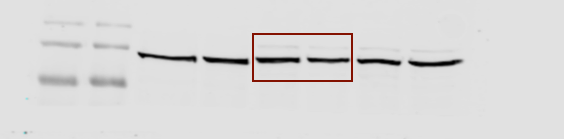

Supplement: Supplementary file 9 — Source data Fig. 3 [file 44321_2025_195_MOESM9_ESM.zip › Figure 3/3K/Figure 3K Vinculin.png]

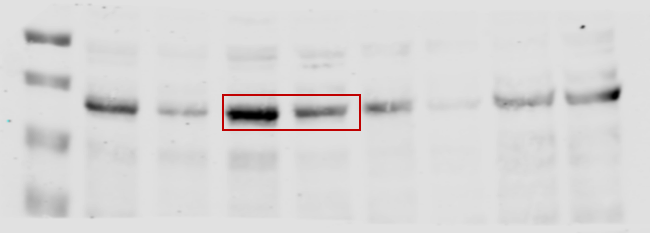

Supplement: Supplementary file 9 — Source data Fig. 3 [file 44321_2025_195_MOESM9_ESM.zip › Figure 3/3L/Figure 3L Olig2.png]

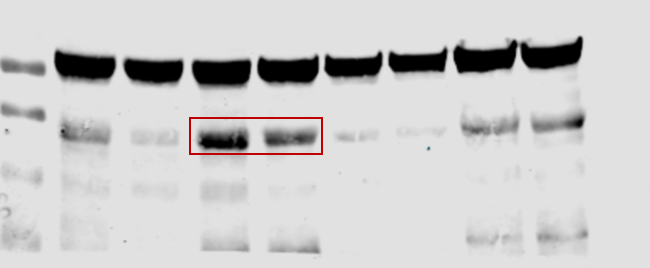

Supplement: Supplementary file 9 — Source data Fig. 3 [file 44321_2025_195_MOESM9_ESM.zip › Figure 3/3L/Figure 3L SOX2.png]

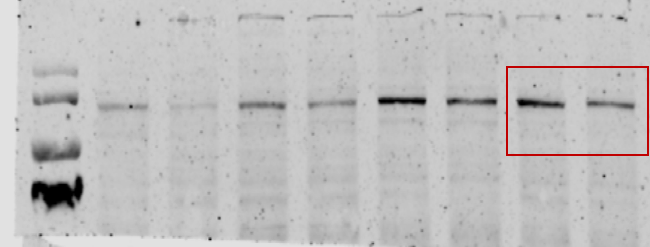

Supplement: Supplementary file 9 — Source data Fig. 3 [file 44321_2025_195_MOESM9_ESM.zip › Figure 3/3L/Figure 3L Cleaved Notch1.png]

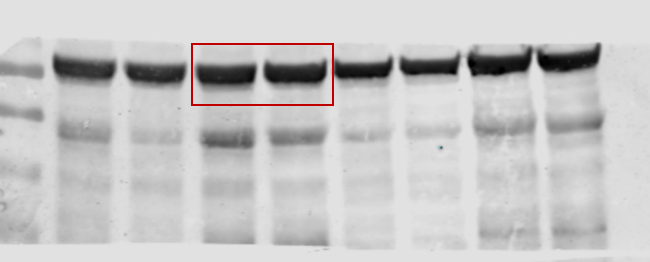

Supplement: Supplementary file 9 — Source data Fig. 3 [file 44321_2025_195_MOESM9_ESM.zip › Figure 3/3L/Figure 3L Tubulin (SOX2:Olig2).png]

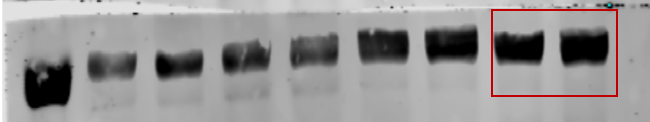

Supplement: Supplementary file 9 — Source data Fig. 3 [file 44321_2025_195_MOESM9_ESM.zip › Figure 3/3L/Figure 3L Tubulin (Cleaved Notch1).png]

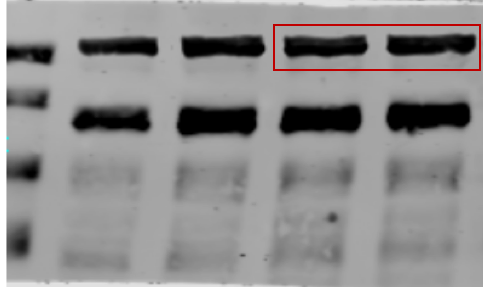

Supplement: Supplementary file 9 — Source data Fig. 3 [file 44321_2025_195_MOESM9_ESM.zip › Figure 3/3P/Figure 3P Tubulin.png]

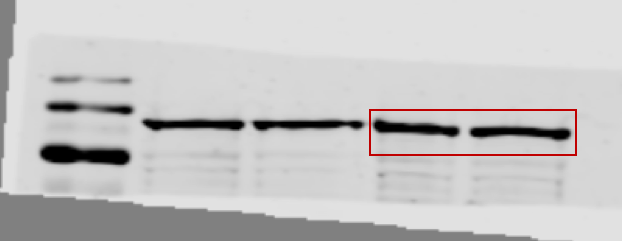

Supplement: Supplementary file 9 — Source data Fig. 3 [file 44321_2025_195_MOESM9_ESM.zip › Figure 3/3P/Figure 3P Vinculin.png]

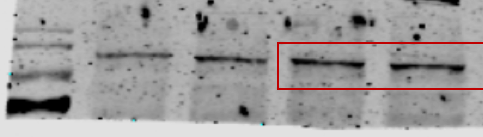

Supplement: Supplementary file 9 — Source data Fig. 3 [file 44321_2025_195_MOESM9_ESM.zip › Figure 3/3P/Figure 3P Cleaved Notch1.png]

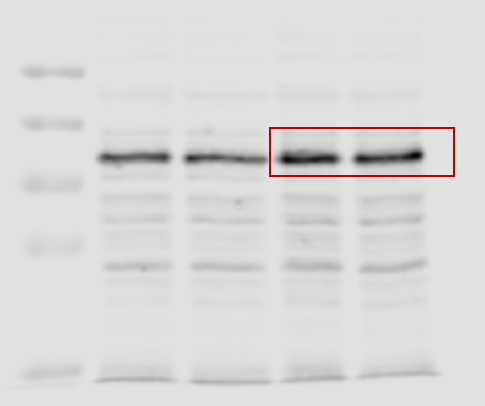

Supplement: Supplementary file 9 — Source data Fig. 3 [file 44321_2025_195_MOESM9_ESM.zip › Figure 3/3P/Figure 3P Olig2.png]

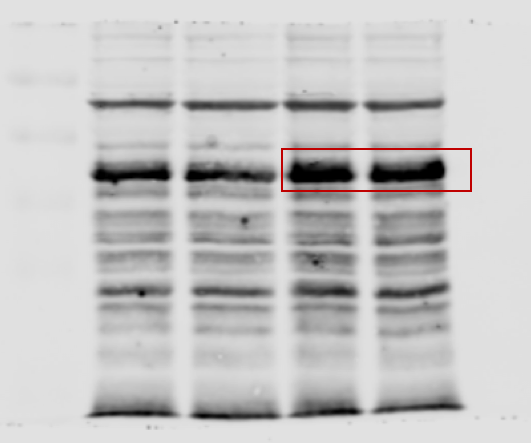

Supplement: Supplementary file 9 — Source data Fig. 3 [file 44321_2025_195_MOESM9_ESM.zip › Figure 3/3P/Figure 3P SOX2.png]

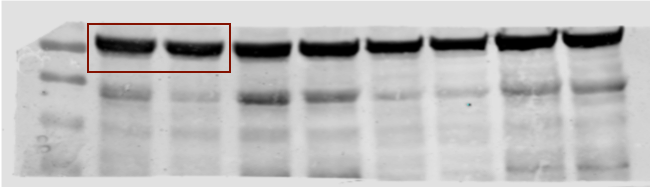

Supplement: Supplementary file 9 — Source data Fig. 3 [file 44321_2025_195_MOESM9_ESM.zip › Figure 3/3J/Figure 3J Tubulin.png]

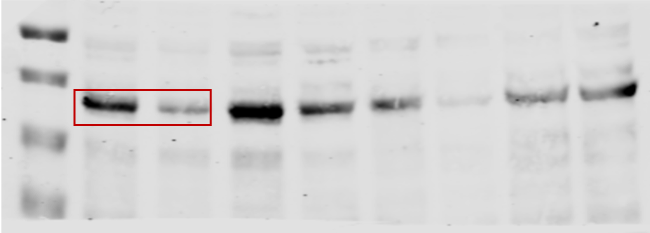

Supplement: Supplementary file 9 — Source data Fig. 3 [file 44321_2025_195_MOESM9_ESM.zip › Figure 3/3J/Figure 3J Olig2.png]

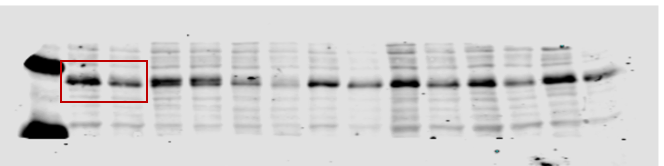

Supplement: Supplementary file 9 — Source data Fig. 3 [file 44321_2025_195_MOESM9_ESM.zip › Figure 3/3J/Figure 3J Cleaved Notch1.png]

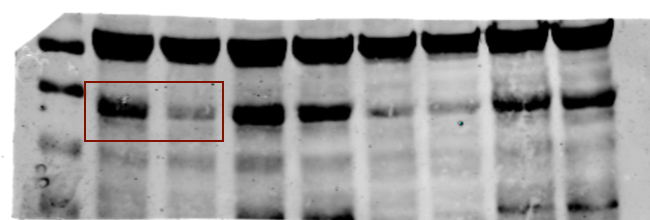

Supplement: Supplementary file 9 — Source data Fig. 3 [file 44321_2025_195_MOESM9_ESM.zip › Figure 3/3J/Figure 3J SOX2.png]

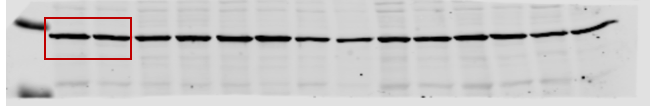

Supplement: Supplementary file 9 — Source data Fig. 3 [file 44321_2025_195_MOESM9_ESM.zip › Figure 3/3J/Figure 3J Vinculin.png]

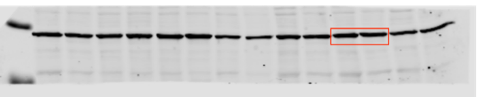

Supplement: Supplementary file 9 — Source data Fig. 3 [file 44321_2025_195_MOESM9_ESM.zip › Figure 3/3H/Figure 3H Vinculin.png]

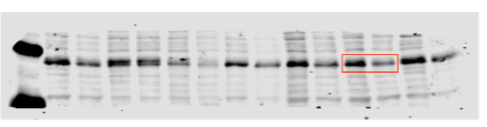

Supplement: Supplementary file 9 — Source data Fig. 3 [file 44321_2025_195_MOESM9_ESM.zip › Figure 3/3H/Figure 3H Cleaved Notch1.png]

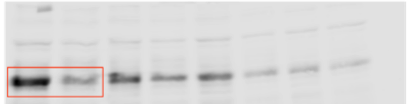

Supplement: Supplementary file 9 — Source data Fig. 3 [file 44321_2025_195_MOESM9_ESM.zip › Figure 3/3H/Figure 3H Olig2.png]

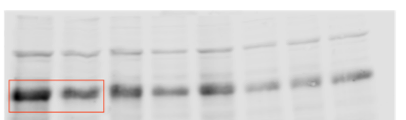

Supplement: Supplementary file 9 — Source data Fig. 3 [file 44321_2025_195_MOESM9_ESM.zip › Figure 3/3H/Figure 3H SOX2.png]

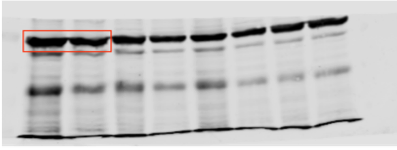

Supplement: Supplementary file 9 — Source data Fig. 3 [file 44321_2025_195_MOESM9_ESM.zip › Figure 3/3H/Figure 3H Tubulin.png]

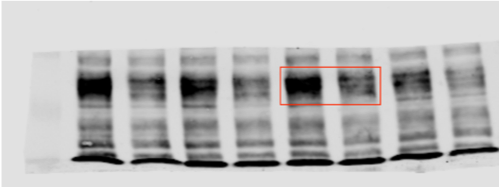

Supplement: Supplementary file 9 — Source data Fig. 3 [file 44321_2025_195_MOESM9_ESM.zip › Figure 3/3G/Figure 3G SOX2.png]

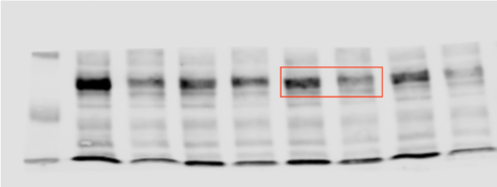

Supplement: Supplementary file 9 — Source data Fig. 3 [file 44321_2025_195_MOESM9_ESM.zip › Figure 3/3G/Figure 3G Olig2.png]

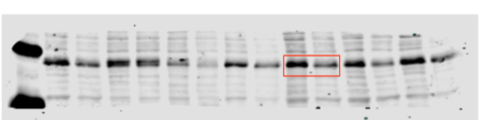

Supplement: Supplementary file 9 — Source data Fig. 3 [file 44321_2025_195_MOESM9_ESM.zip › Figure 3/3G/Figure 3G Cleaved Notch1.png]

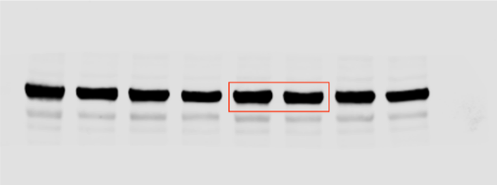

Supplement: Supplementary file 9 — Source data Fig. 3 [file 44321_2025_195_MOESM9_ESM.zip › Figure 3/3G/Figure 3G Tubulin.png]

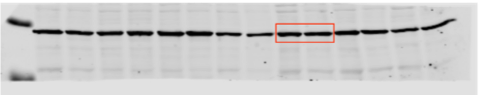

Supplement: Supplementary file 9 — Source data Fig. 3 [file 44321_2025_195_MOESM9_ESM.zip › Figure 3/3G/Figure 3G Vinculin.png]

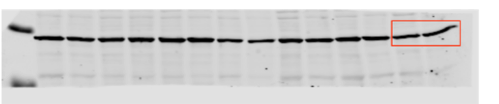

Supplement: Supplementary file 9 — Source data Fig. 3 [file 44321_2025_195_MOESM9_ESM.zip › Figure 3/3I/Figure 3I Vinculin.png]

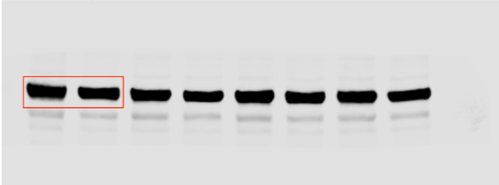

Supplement: Supplementary file 9 — Source data Fig. 3 [file 44321_2025_195_MOESM9_ESM.zip › Figure 3/3I/Figure 3I Tubulin.png]

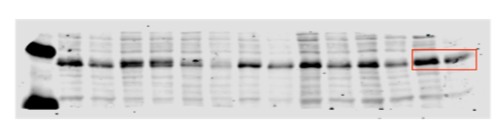

Supplement: Supplementary file 9 — Source data Fig. 3 [file 44321_2025_195_MOESM9_ESM.zip › Figure 3/3I/Figure 3I Cleaved Notch1.png]

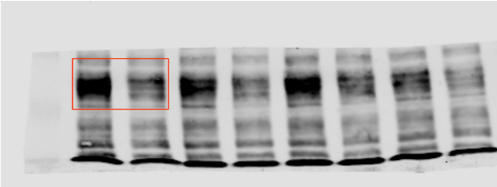

Supplement: Supplementary file 9 — Source data Fig. 3 [file 44321_2025_195_MOESM9_ESM.zip › Figure 3/3I/Figure 3I SOX2.png]

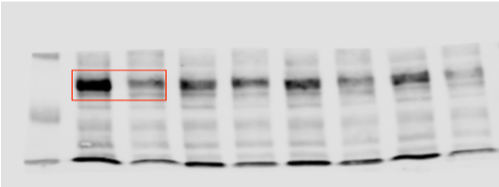

Supplement: Supplementary file 9 — Source data Fig. 3 [file 44321_2025_195_MOESM9_ESM.zip › Figure 3/3I/Figure 3I Olig2.png]

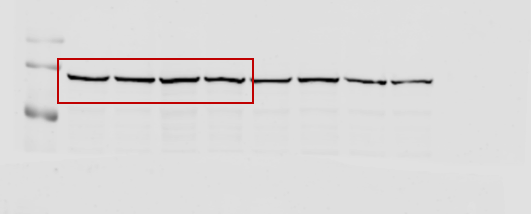

Supplement: Supplementary file 9 — Source data Fig. 3 [file 44321_2025_195_MOESM9_ESM.zip › Figure 3/3N/Figure 3N Vinculin.png]

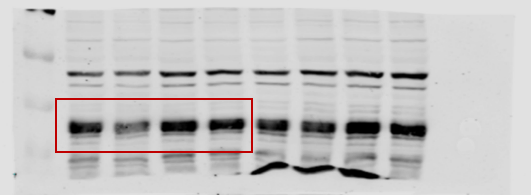

Supplement: Supplementary file 9 — Source data Fig. 3 [file 44321_2025_195_MOESM9_ESM.zip › Figure 3/3N/Figure 3N SOX2.png]

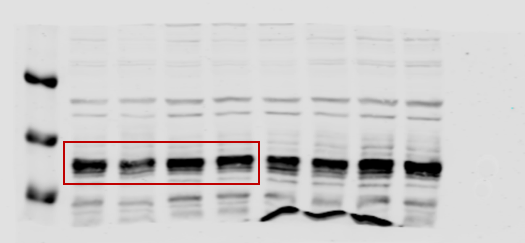

Supplement: Supplementary file 9 — Source data Fig. 3 [file 44321_2025_195_MOESM9_ESM.zip › Figure 3/3N/Figure 3N Olig2.png]

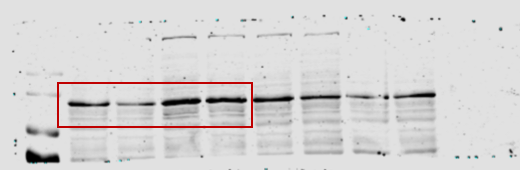

Supplement: Supplementary file 9 — Source data Fig. 3 [file 44321_2025_195_MOESM9_ESM.zip › Figure 3/3N/Figure 3N Cleaved Notch1.png]

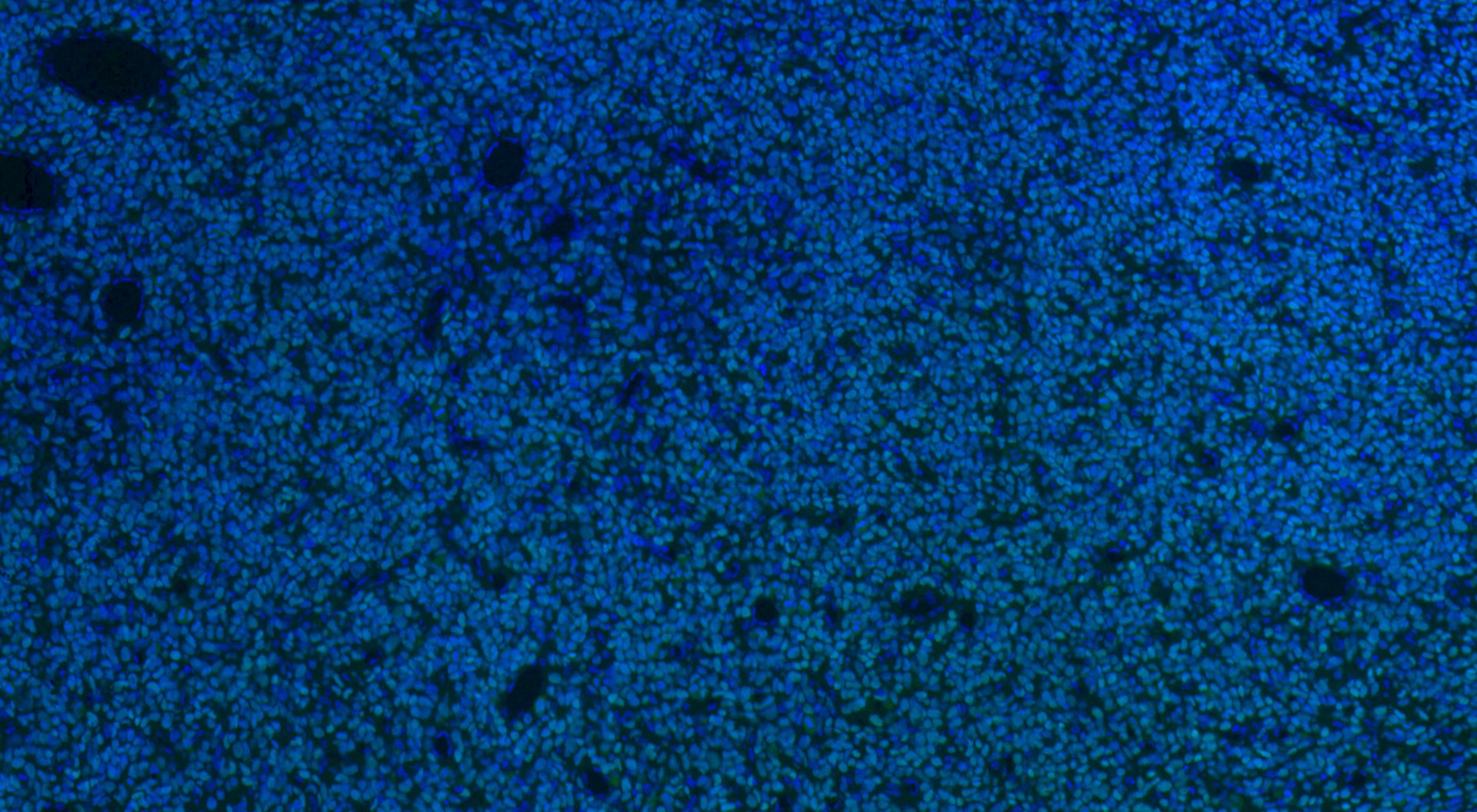

Supplement: Supplementary file 10 — Source data Fig. 4 [file 44321_2025_195_MOESM10_ESM.zip › Figure 4/4K/Figure 4K Olig2 DAPI Vehicle control.jpg]

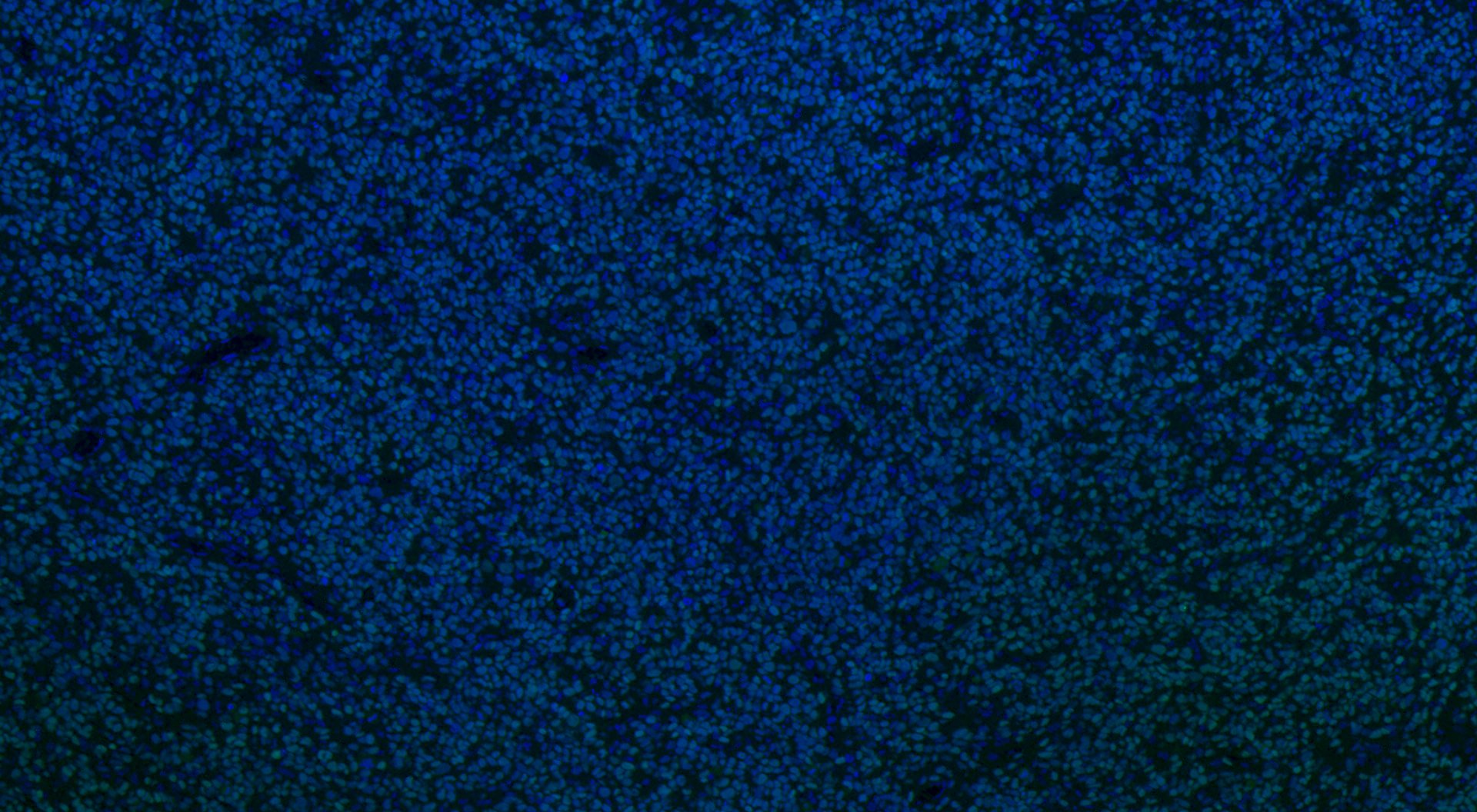

Supplement: Supplementary file 10 — Source data Fig. 4 [file 44321_2025_195_MOESM10_ESM.zip › Figure 4/4K/Figure 4K Olig2 DAPI Mubritinib.jpg]

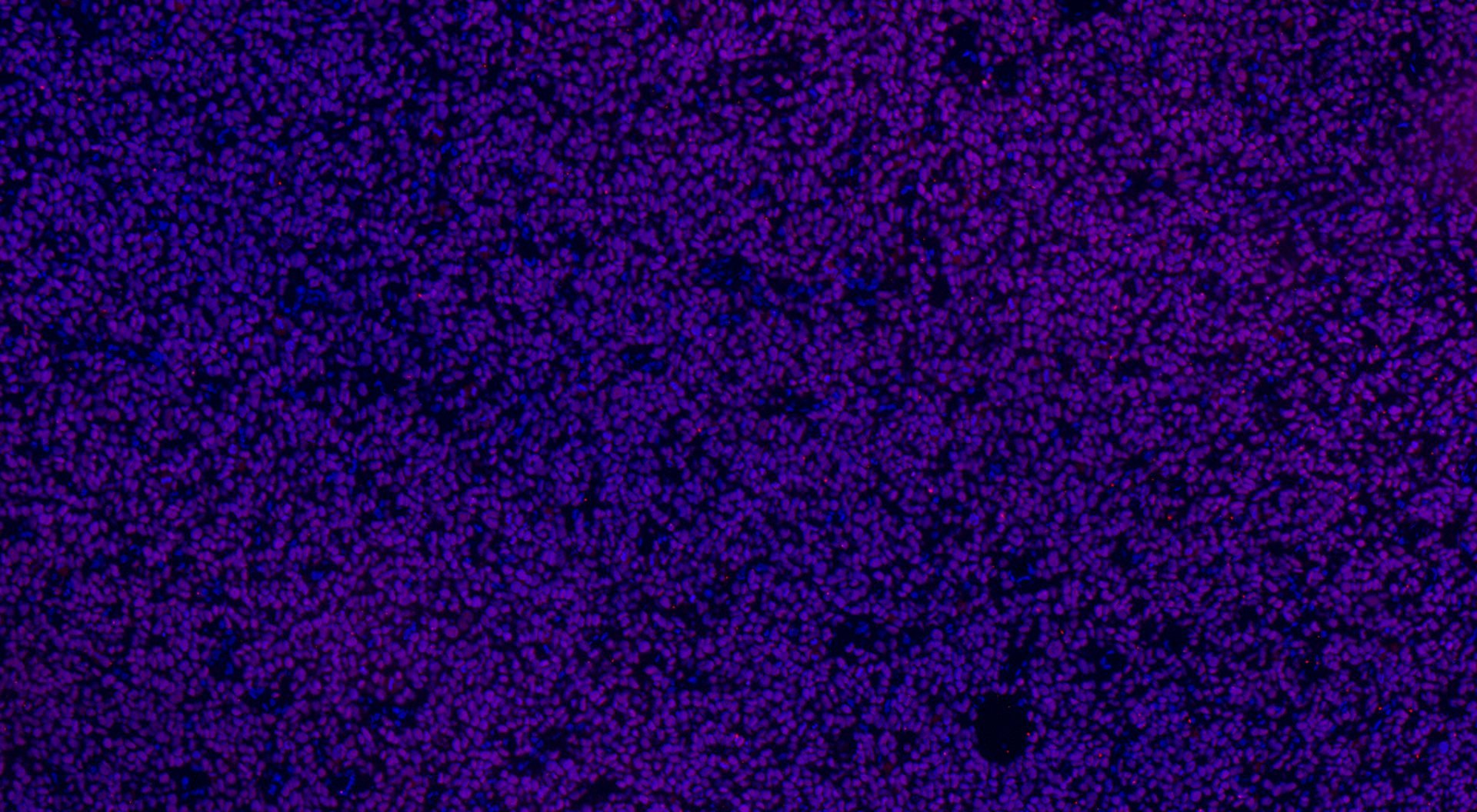

Supplement: Supplementary file 10 — Source data Fig. 4 [file 44321_2025_195_MOESM10_ESM.zip › Figure 4/4L/Figure 4L SOX2 DAPI Mubritinib.jpg]

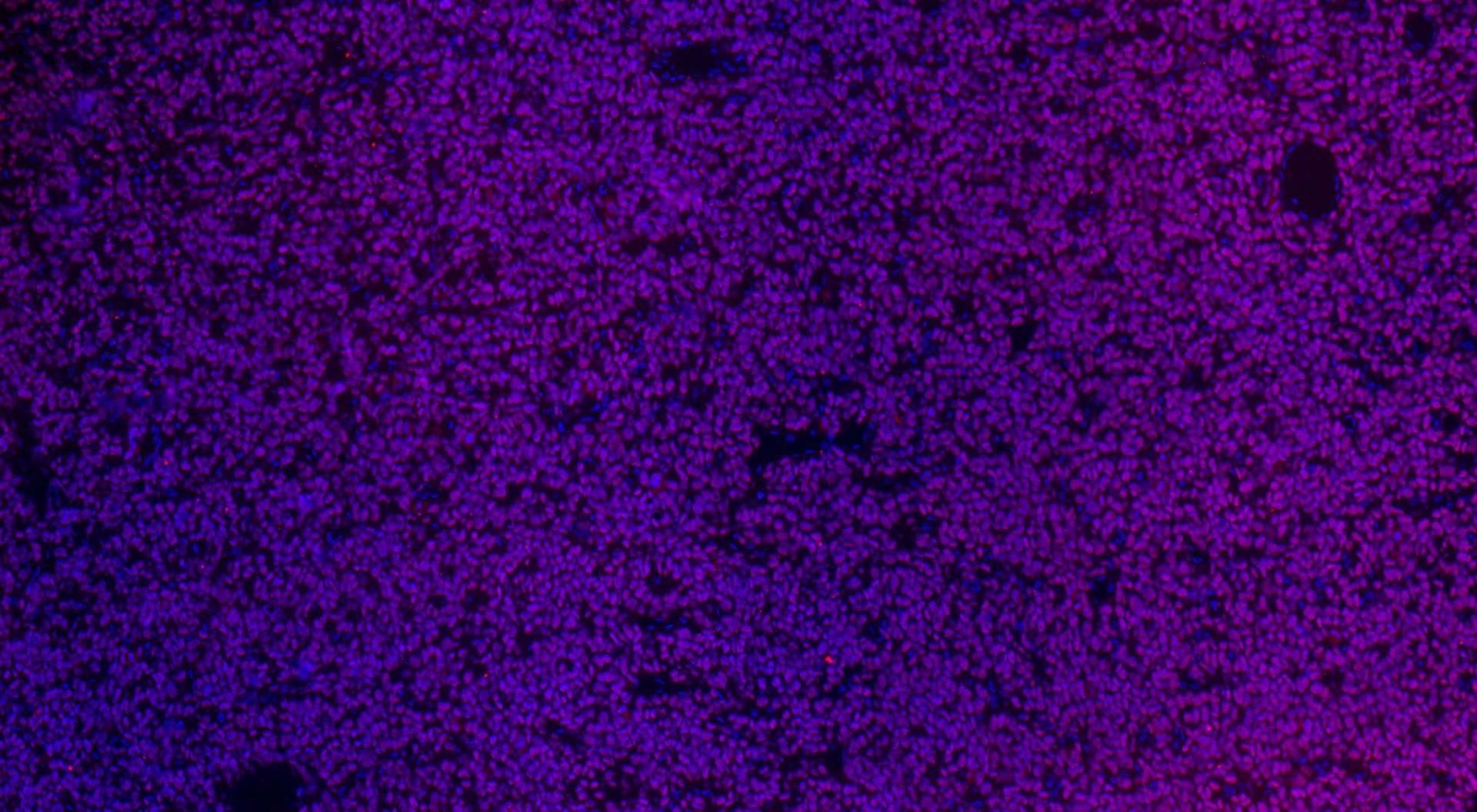

Supplement: Supplementary file 10 — Source data Fig. 4 [file 44321_2025_195_MOESM10_ESM.zip › Figure 4/4L/Figure 4L SOX2 DAPI vehicle control.jpg]

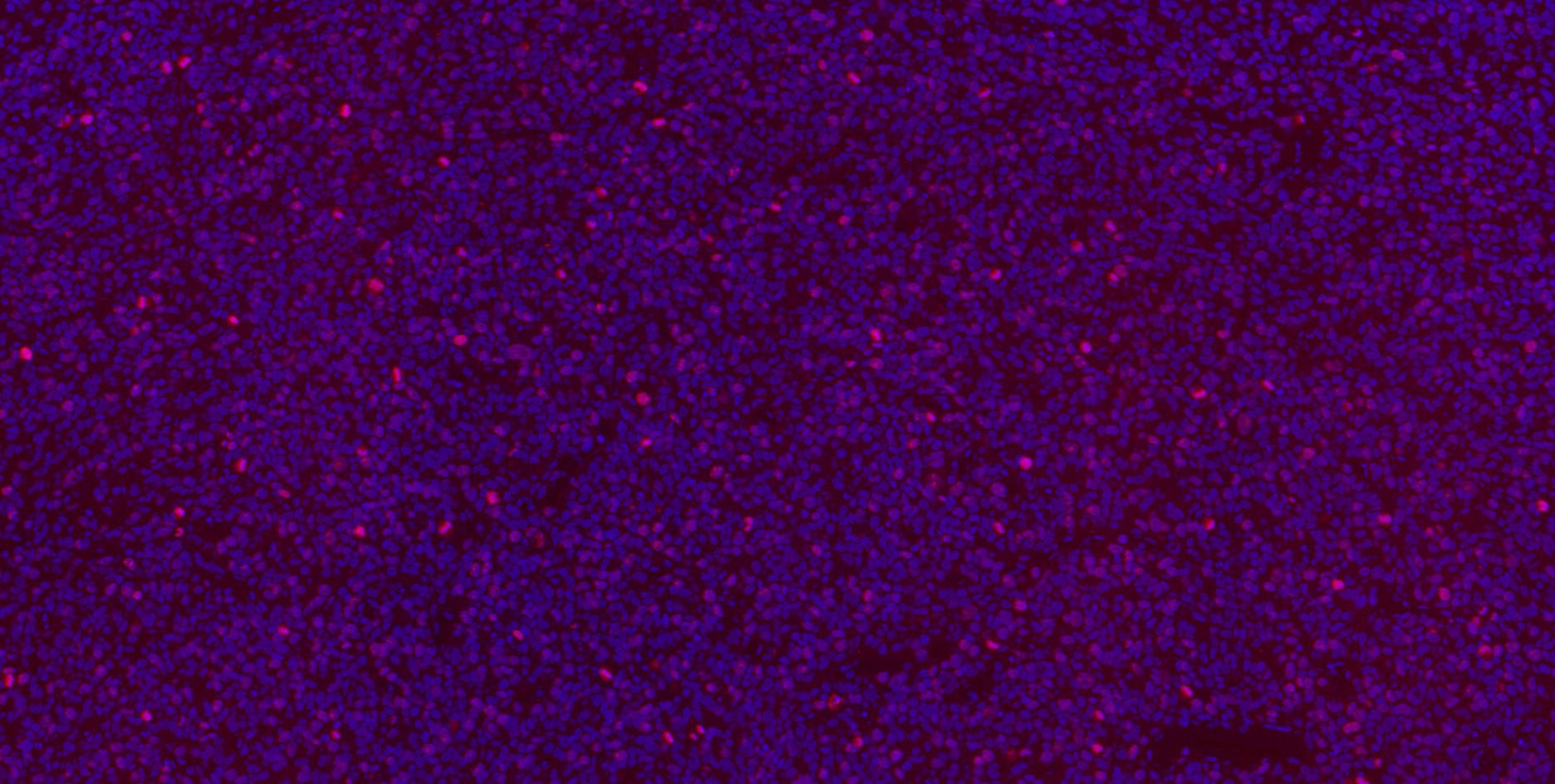

Supplement: Supplementary file 10 — Source data Fig. 4 [file 44321_2025_195_MOESM10_ESM.zip › Figure 4/4M/Figure 4M Ki67 DAPI Mubritinib.jpg]

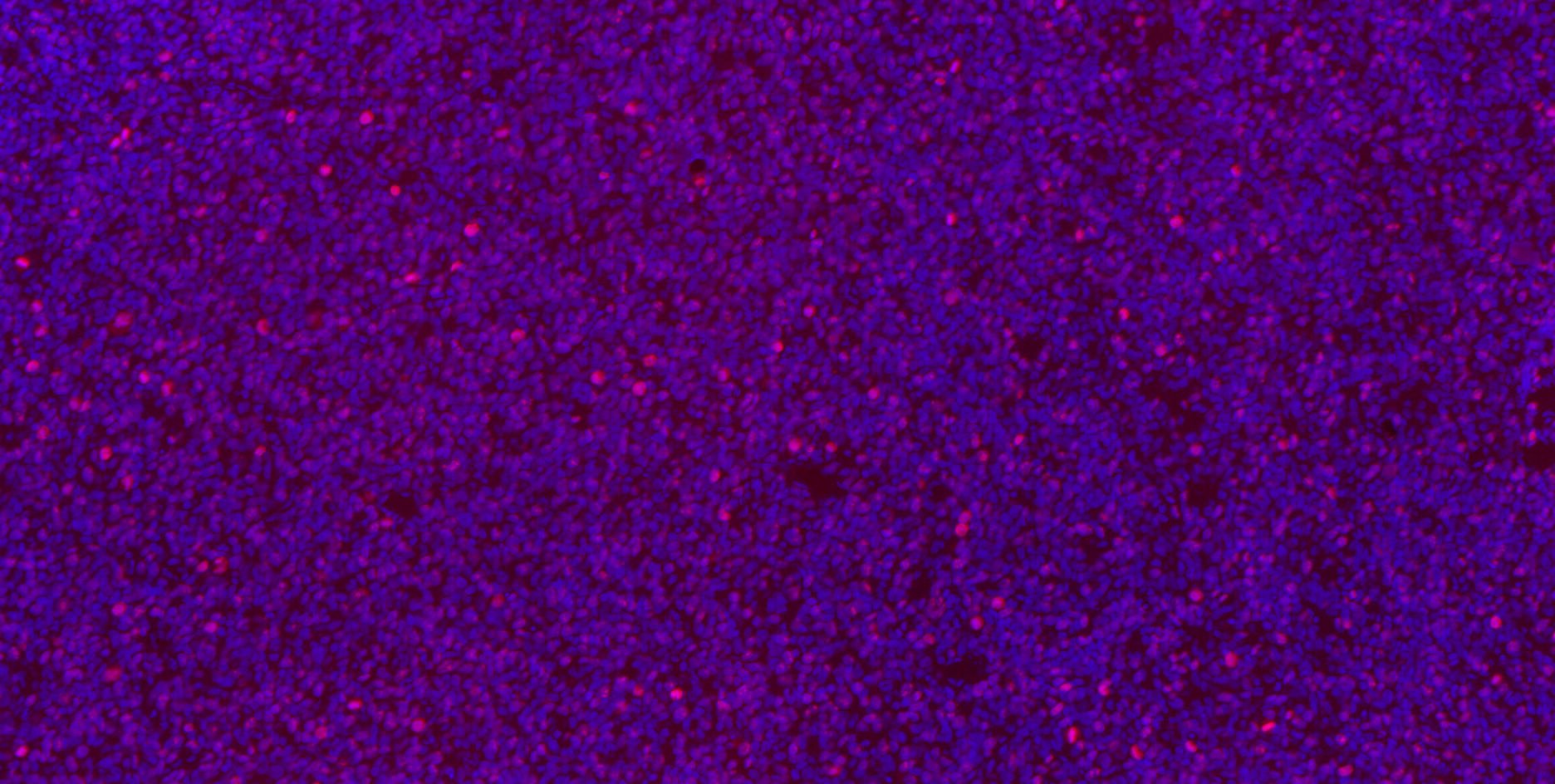

Supplement: Supplementary file 10 — Source data Fig. 4 [file 44321_2025_195_MOESM10_ESM.zip › Figure 4/4M/Figure 4M Ki67 DAPI vehicle control.jpg]

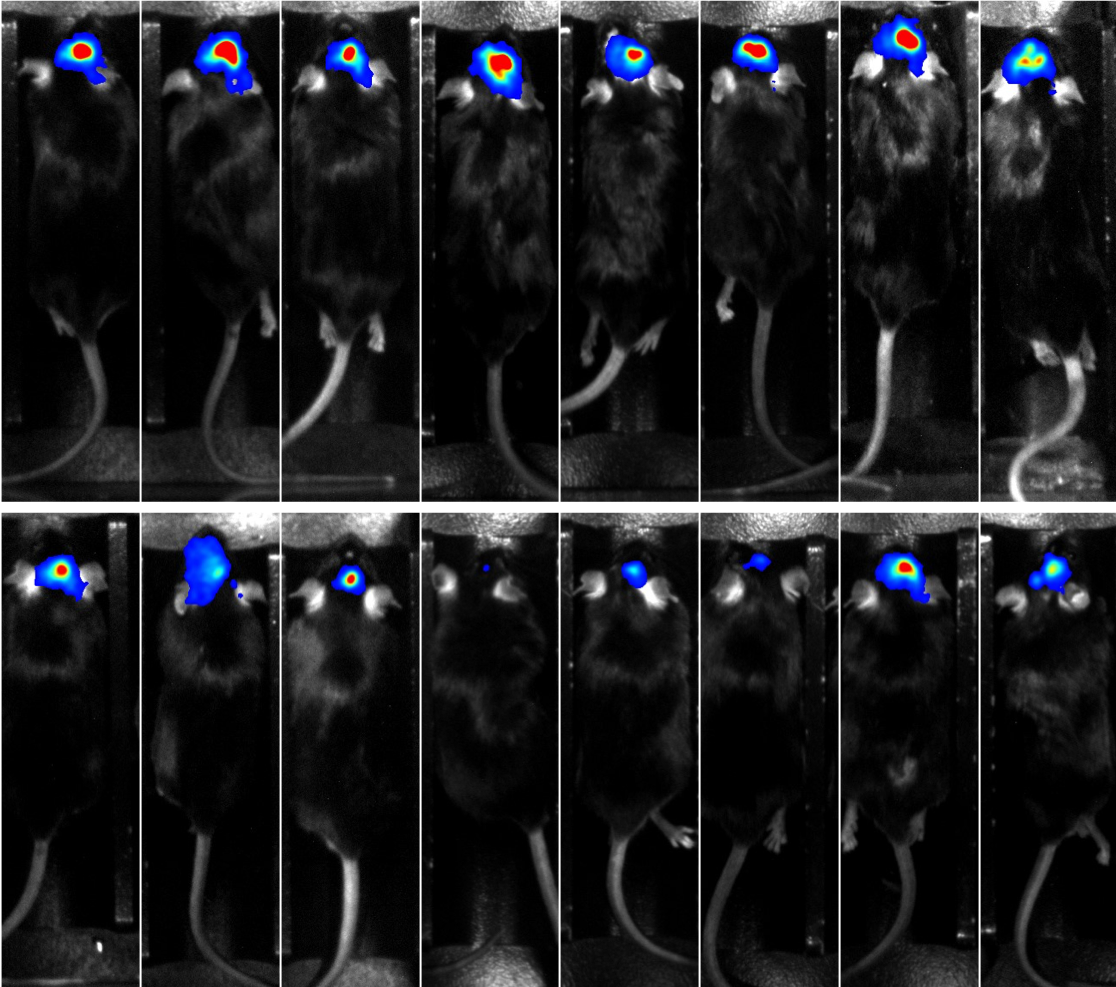

Supplement: Supplementary file 10 — Source data Fig. 4 [file 44321_2025_195_MOESM10_ESM.zip › Figure 4/4D/Figure 4D.png]

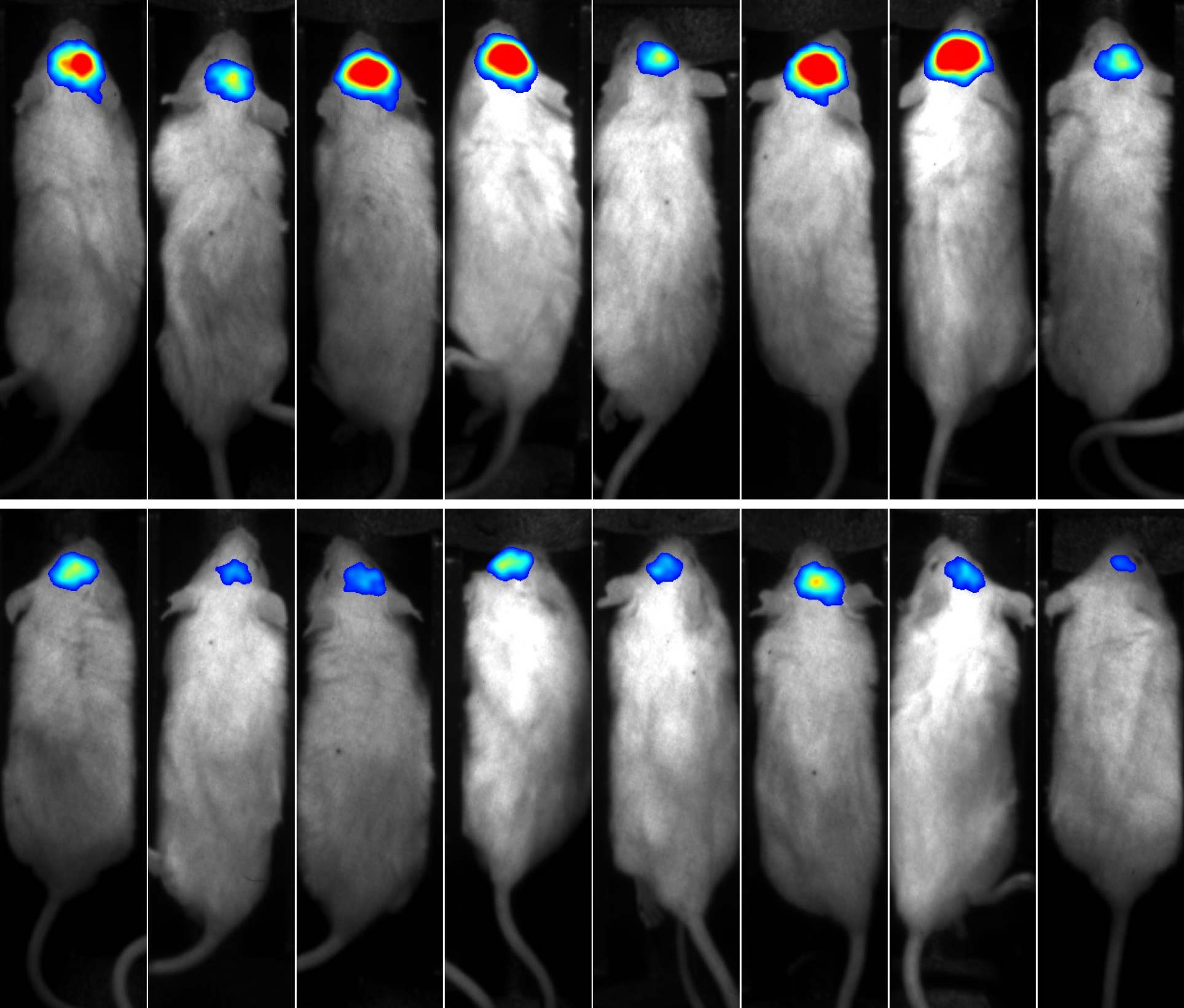

Supplement: Supplementary file 10 — Source data Fig. 4 [file 44321_2025_195_MOESM10_ESM.zip › Figure 4/4G/Figure 4G.png]

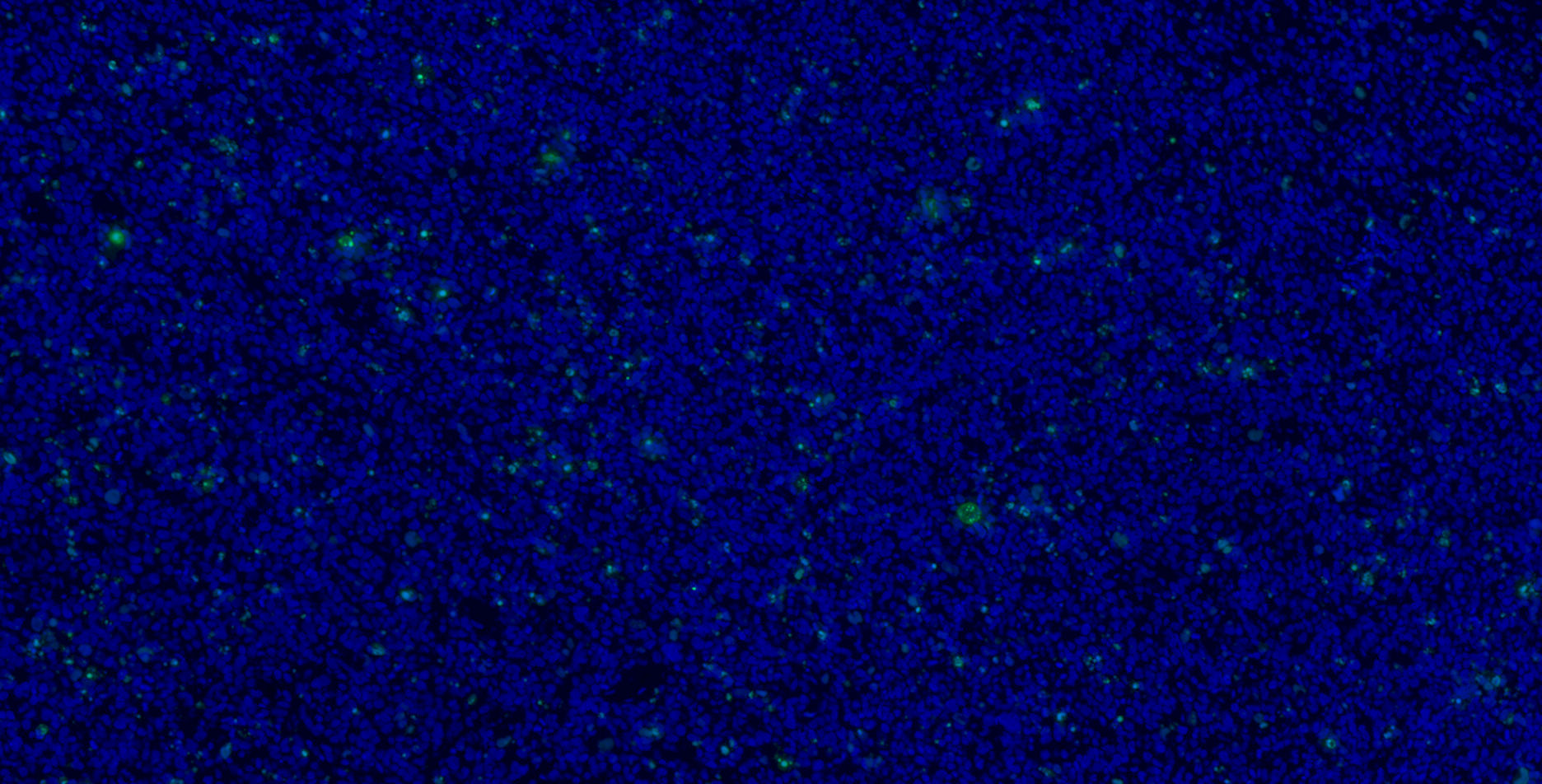

Supplement: Supplementary file 11 — Source data Fig. 5 [file 44321_2025_195_MOESM11_ESM.zip › Figure 5/5N/Figure 5N IR pH2AX DAPI.jpg]

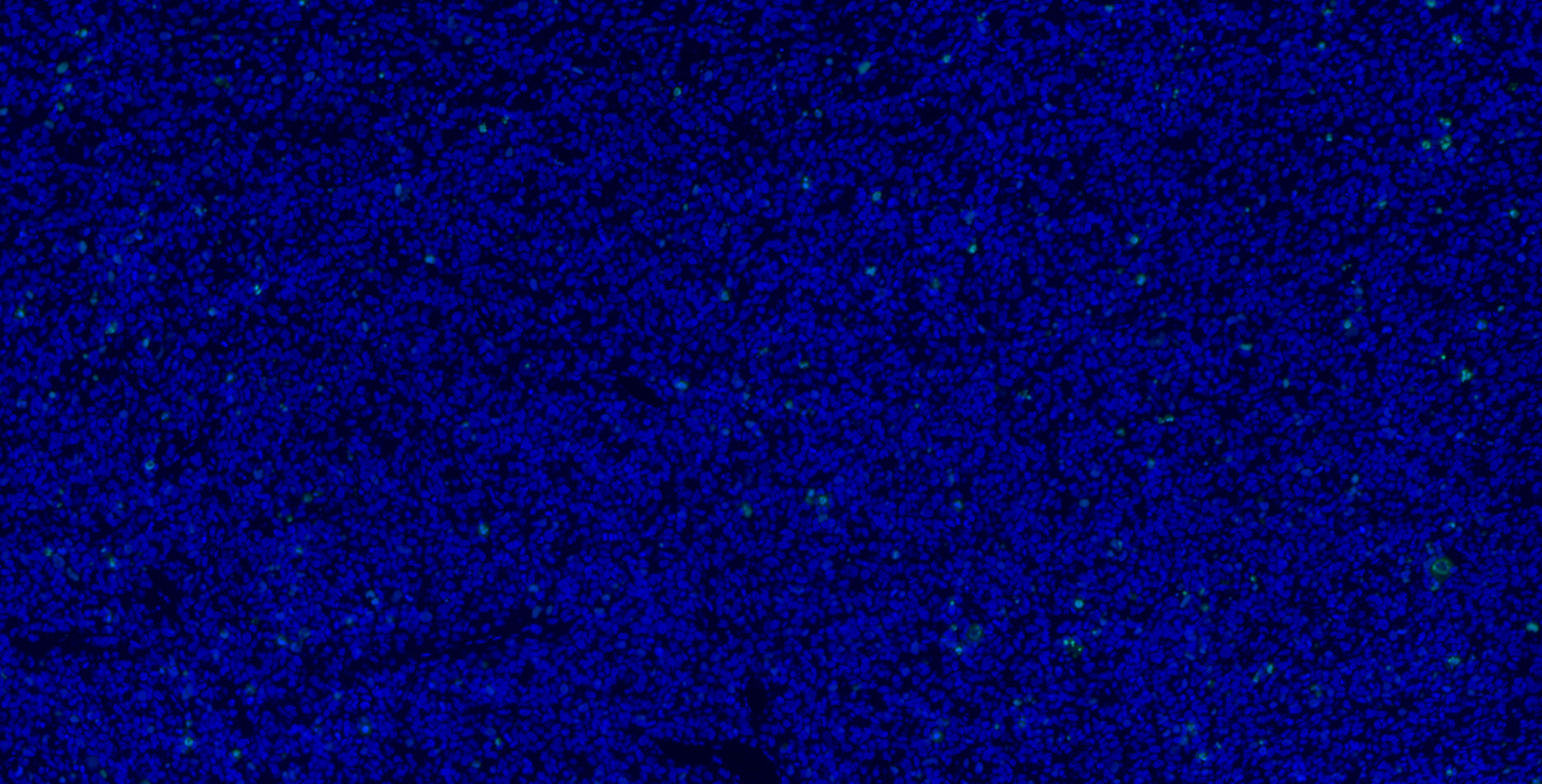

Supplement: Supplementary file 11 — Source data Fig. 5 [file 44321_2025_195_MOESM11_ESM.zip › Figure 5/5N/Figure 5N Vehicle control pH2AX DAPI.jpg]

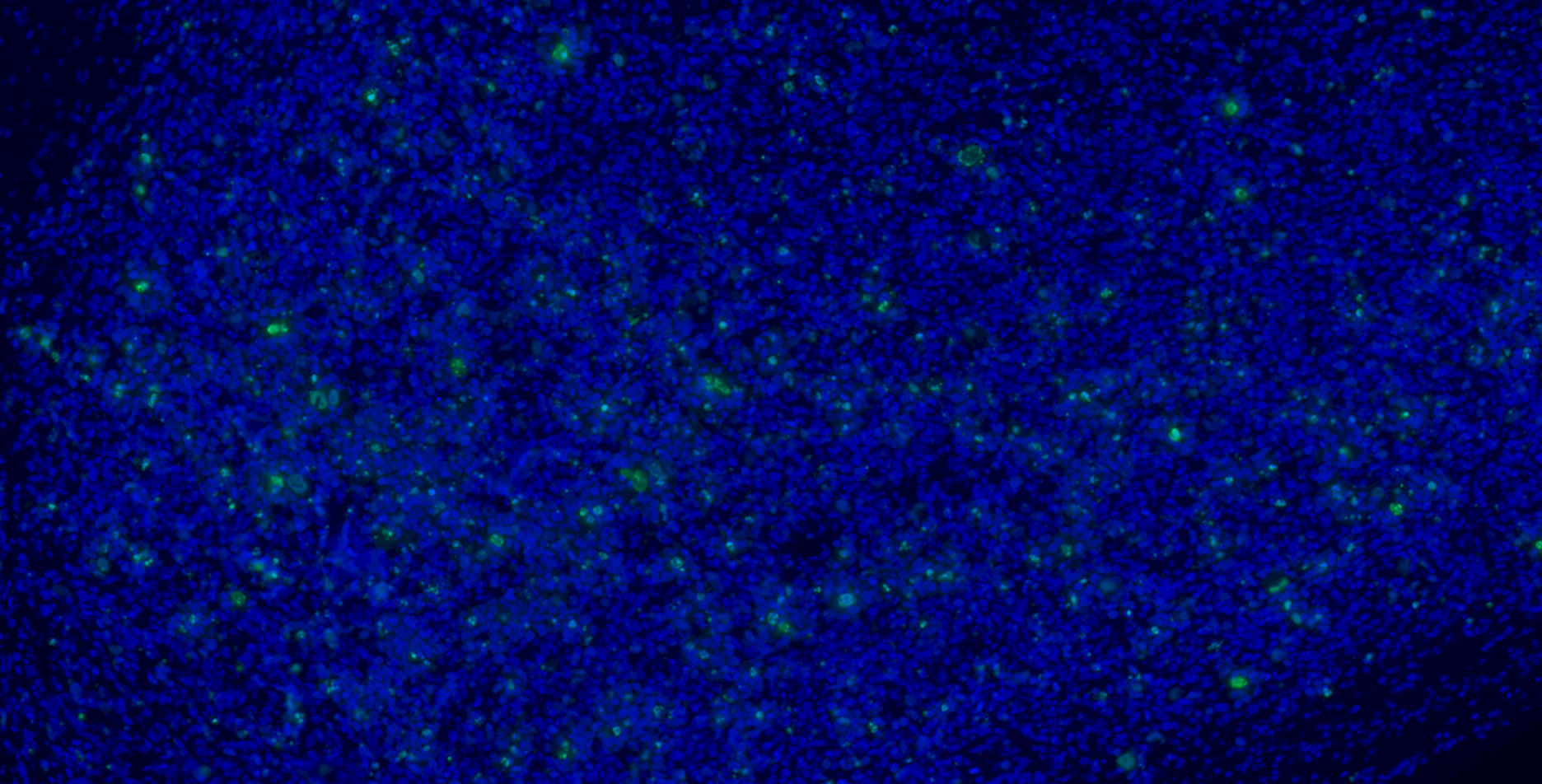

Supplement: Supplementary file 11 — Source data Fig. 5 [file 44321_2025_195_MOESM11_ESM.zip › Figure 5/5N/Figure 5N mubritinib + IR pH2AX DAPI.jpg]

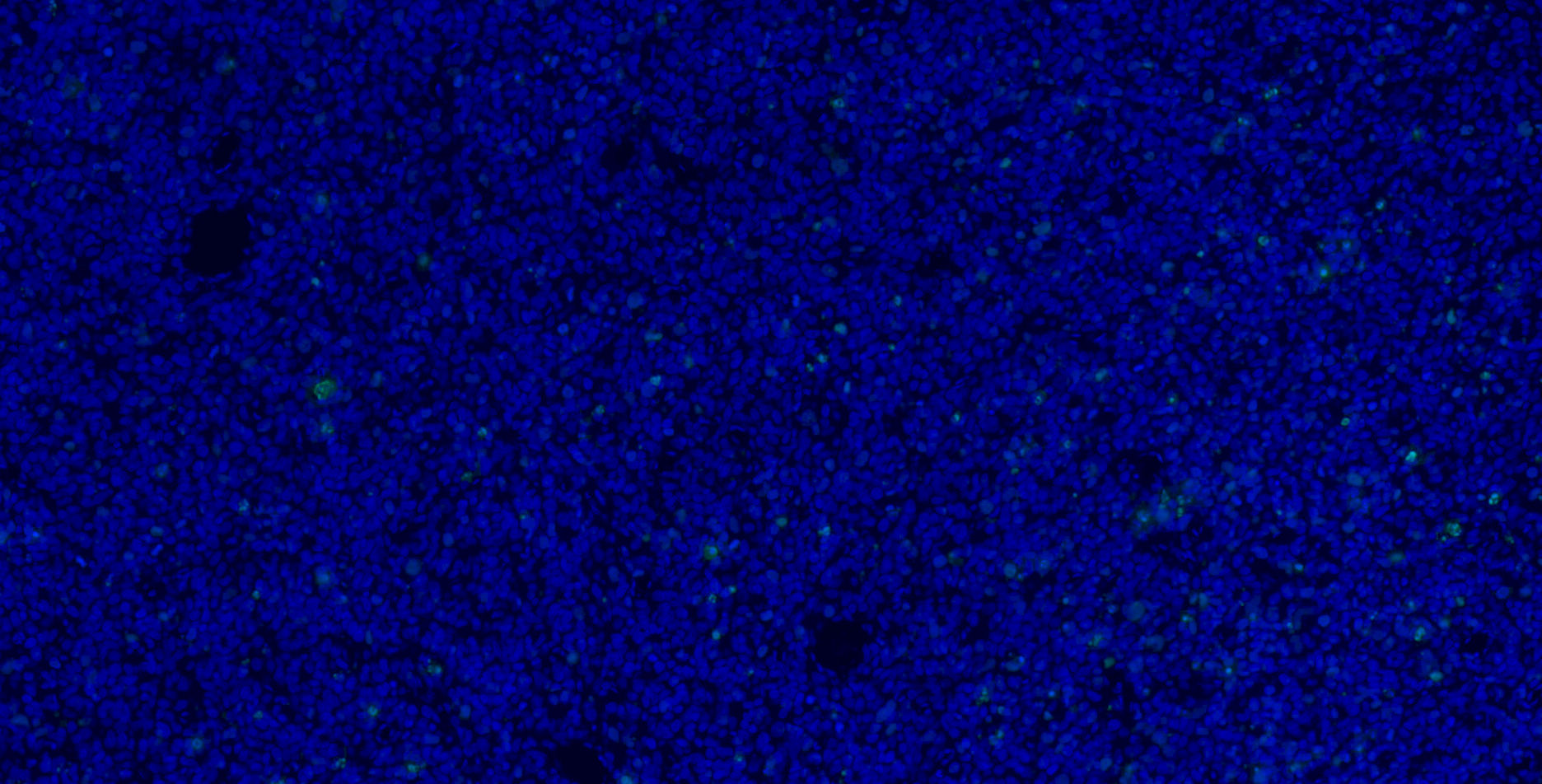

Supplement: Supplementary file 11 — Source data Fig. 5 [file 44321_2025_195_MOESM11_ESM.zip › Figure 5/5N/Figure 5N mubritinib pH2AX DAPI.jpg]

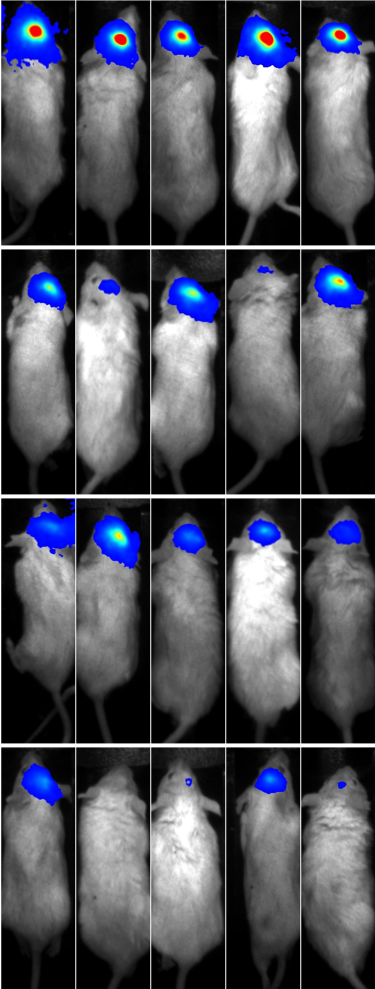

Supplement: Supplementary file 11 — Source data Fig. 5 [file 44321_2025_195_MOESM11_ESM.zip › Figure 5/5F/Figure 5F.png]

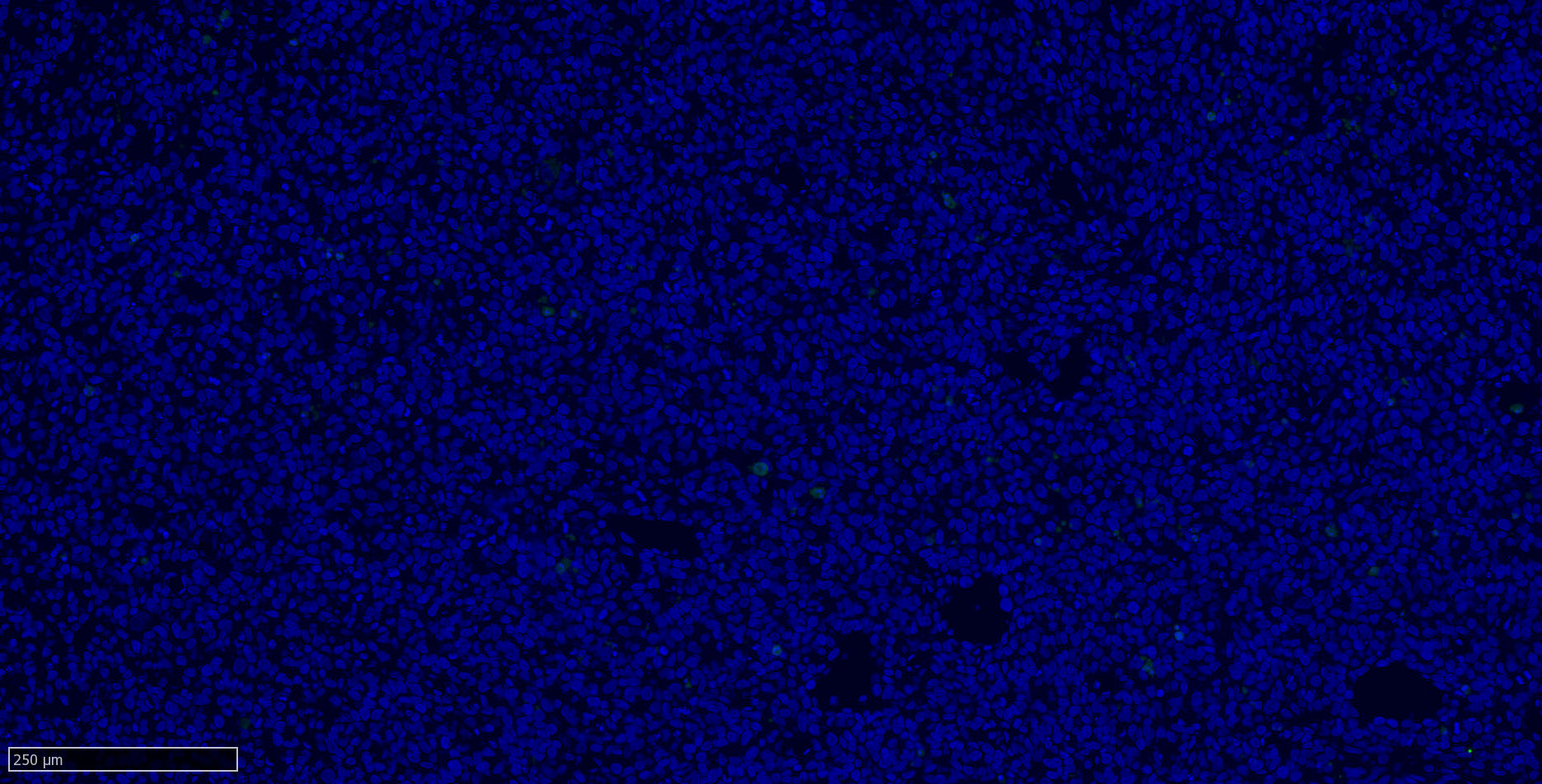

Supplement: Supplementary file 11 — Source data Fig. 5 [file 44321_2025_195_MOESM11_ESM.zip › Figure 5/5O/Figure 5O CC3 DAPI mubritinib.jpg]

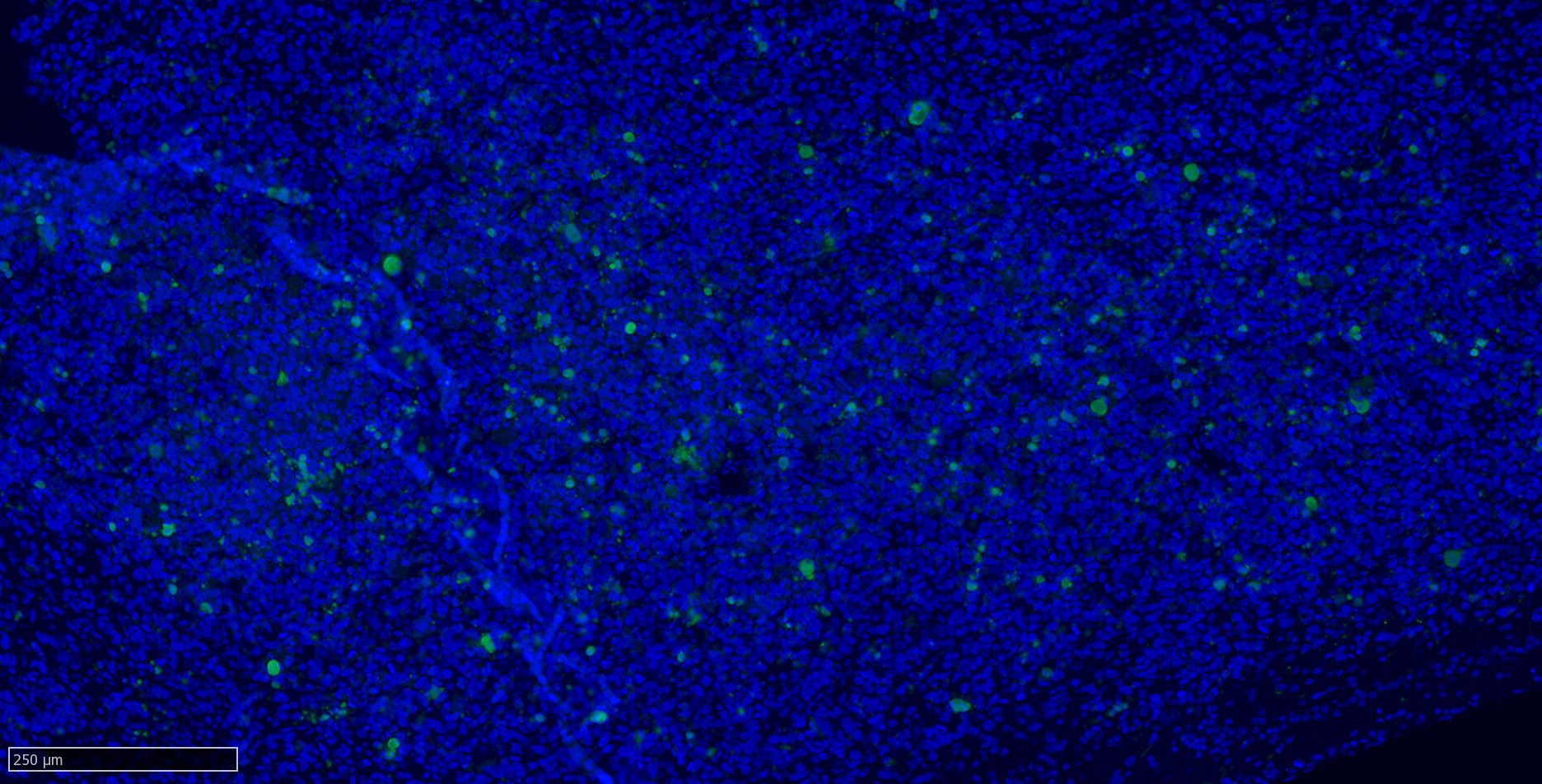

Supplement: Supplementary file 11 — Source data Fig. 5 [file 44321_2025_195_MOESM11_ESM.zip › Figure 5/5O/Figure 5O CC3 DAPI mubritinib + IR.jpg]

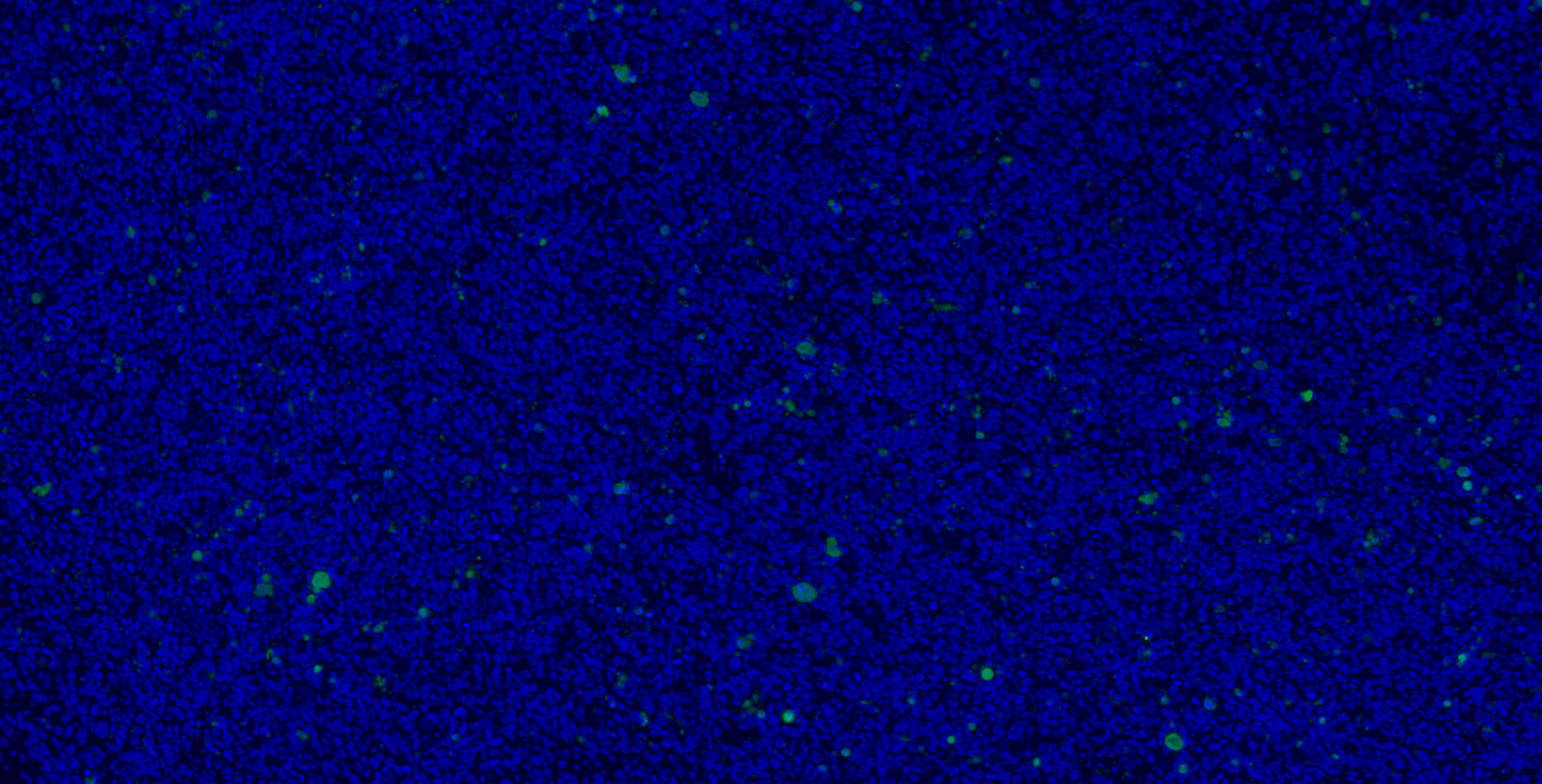

Supplement: Supplementary file 11 — Source data Fig. 5 [file 44321_2025_195_MOESM11_ESM.zip › Figure 5/5O/Figure 5O CC3 DAPI IR.jpg]

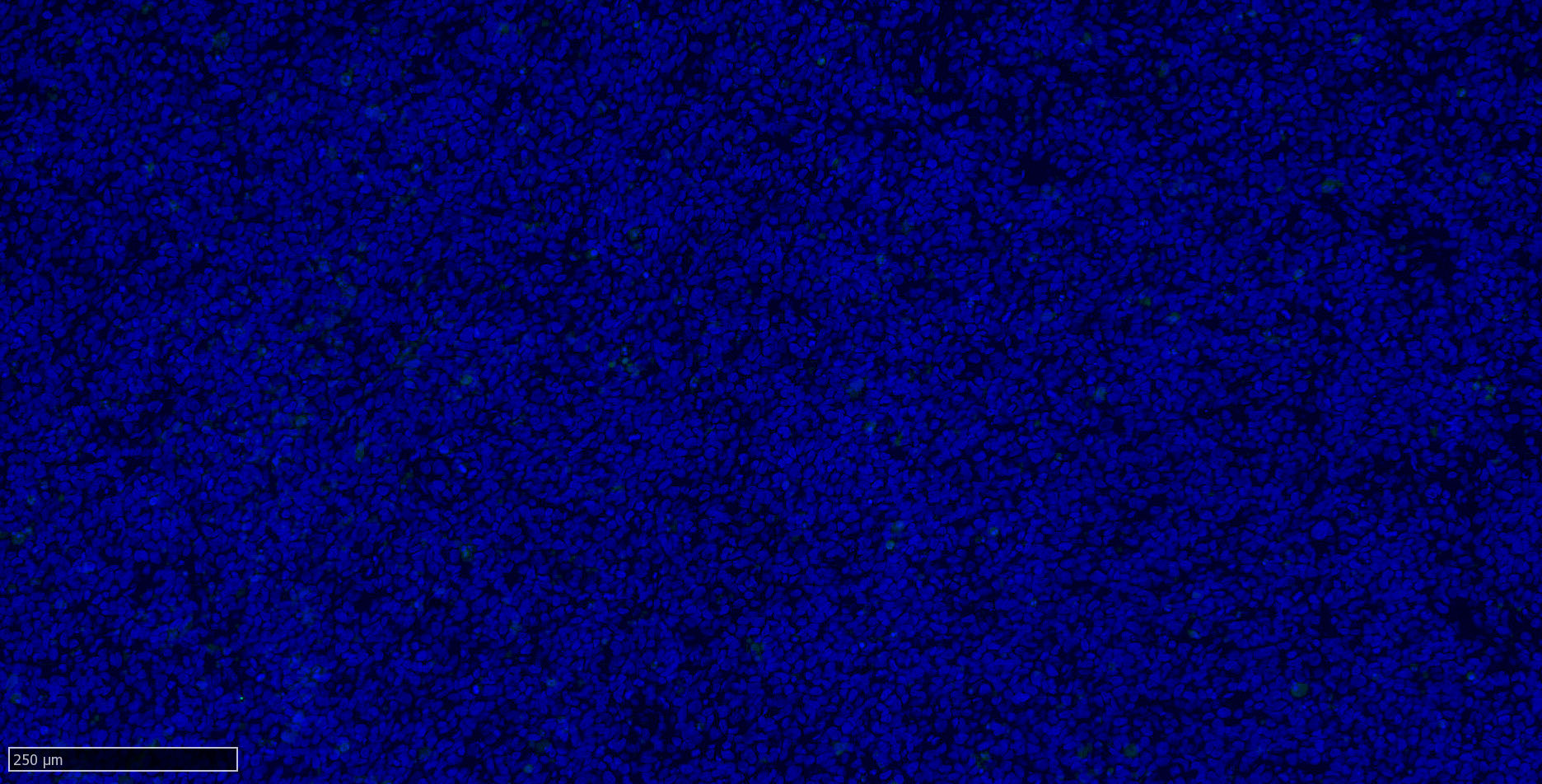

Supplement: Supplementary file 11 — Source data Fig. 5 [file 44321_2025_195_MOESM11_ESM.zip › Figure 5/5O/Figure 5O CC3 DAPI vehicle control.jpg]

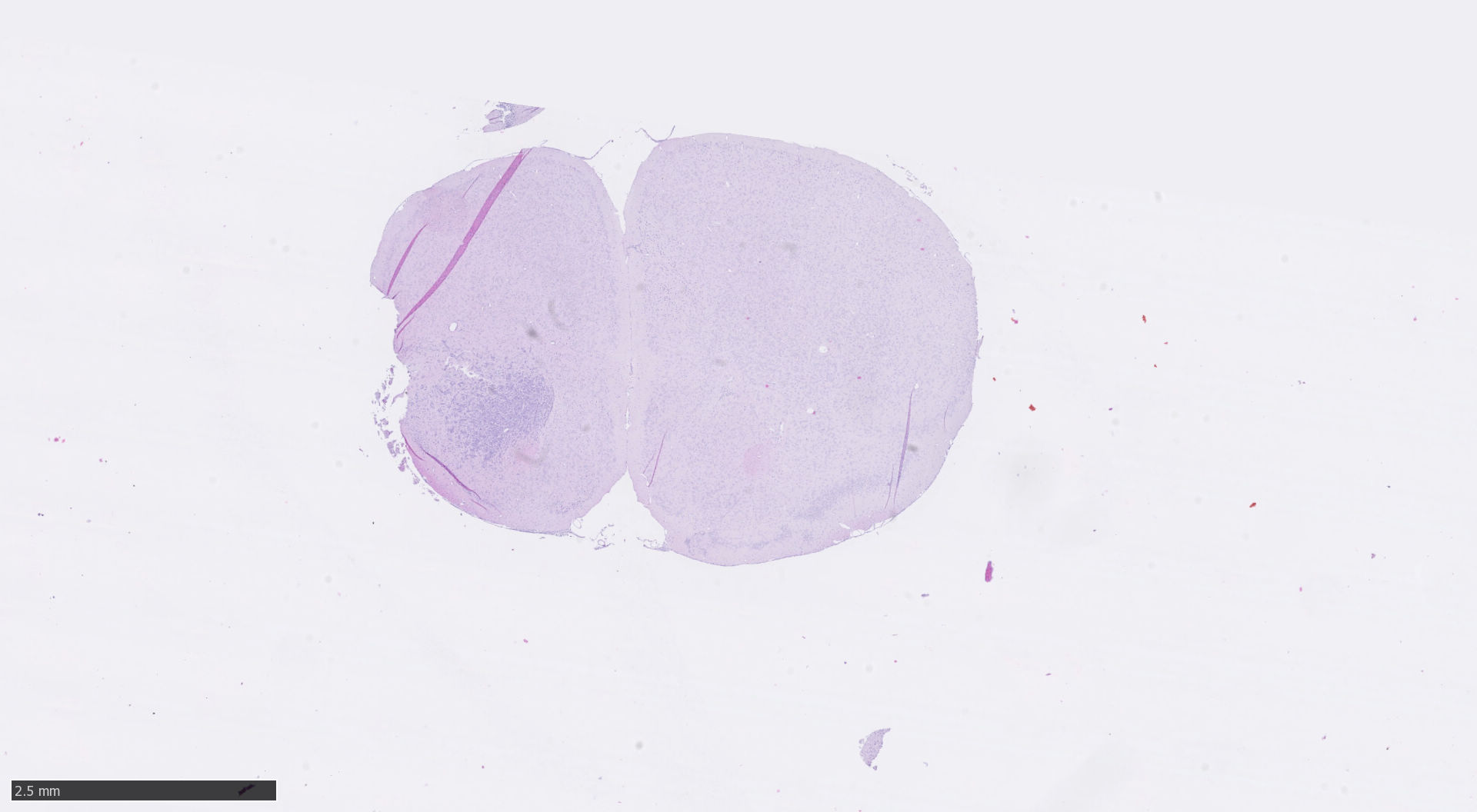

Supplement: Supplementary file 11 — Source data Fig. 5 [file 44321_2025_195_MOESM11_ESM.zip › Figure 5/5H/Mubritinib + IR.jpg]

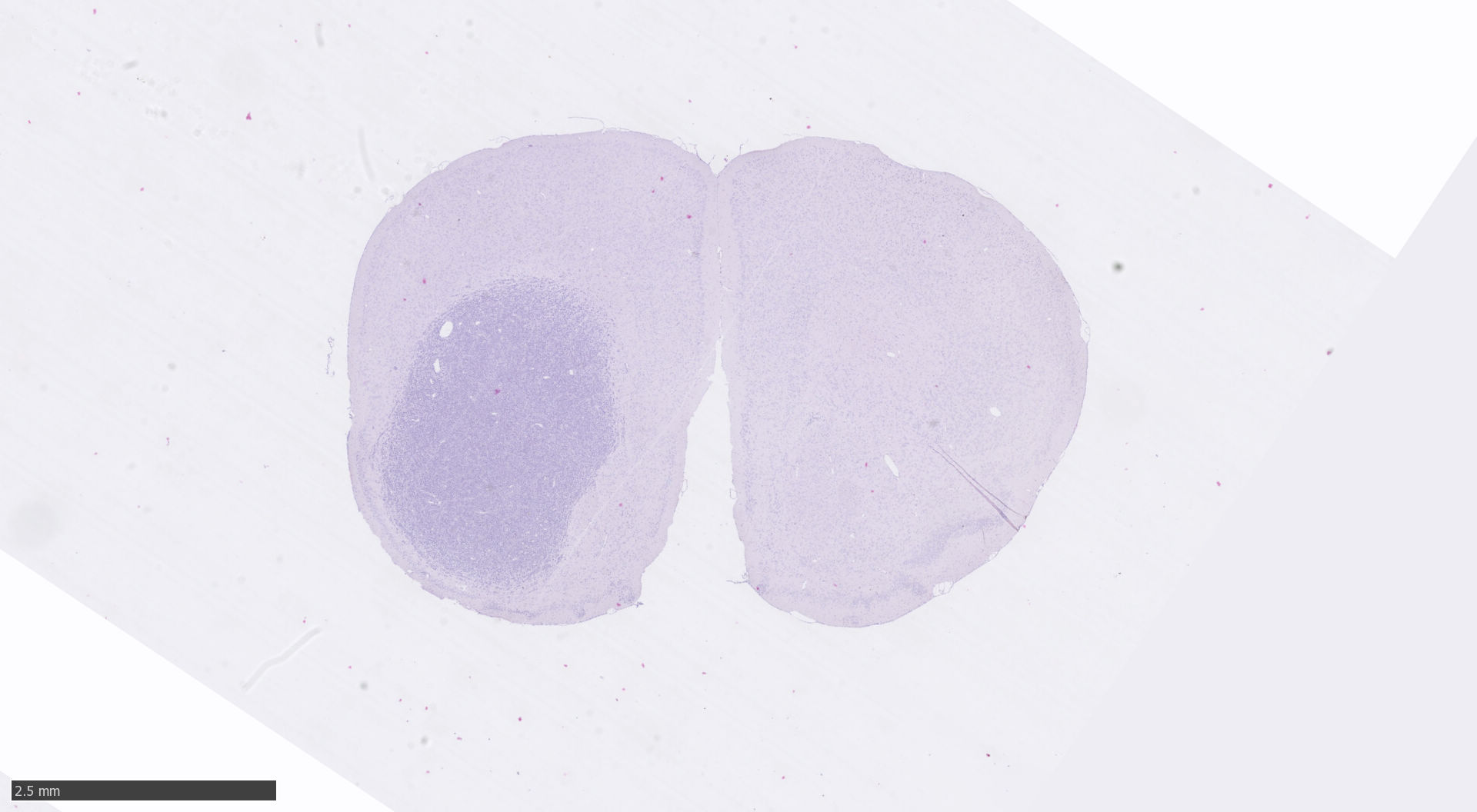

Supplement: Supplementary file 11 — Source data Fig. 5 [file 44321_2025_195_MOESM11_ESM.zip › Figure 5/5H/IR.jpg]
