# Supplementary figures and images for: Exploiting metabolic vulnerability in glioblastoma using a brain-penetrant drug with a safe profile (part 2 of 2)
Source: EMBO Mol Med. 2025 Feb 3;17(3):469–503. doi: 10.1038/s44321-025-00195-6 (PMC11903783; doi:10.1038/s44321-025-00195-6)

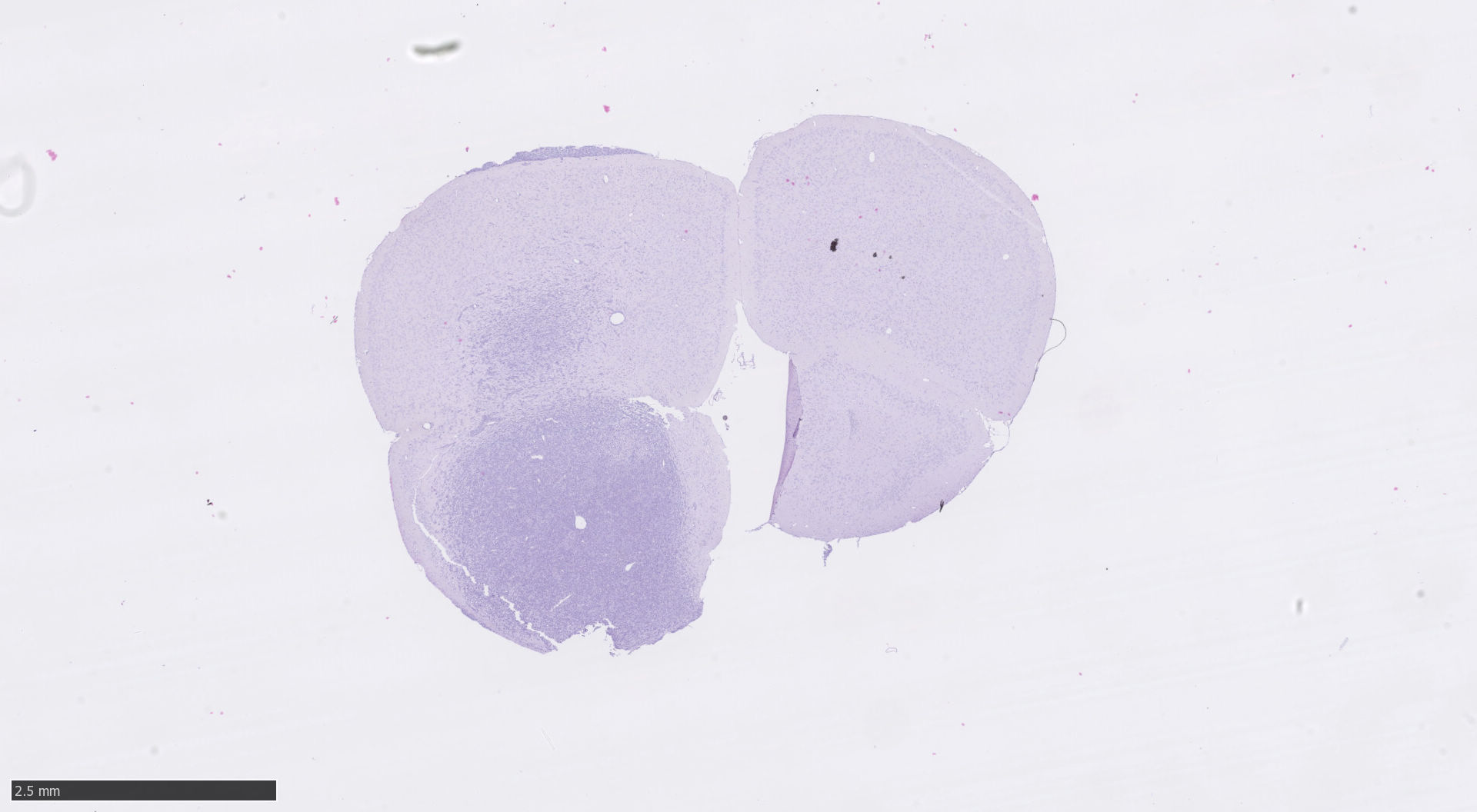

Supplement: Supplementary file 11 — Source data Fig. 5 [file 44321_2025_195_MOESM11_ESM.zip › Figure 5/5H/Mubritinib.jpg]

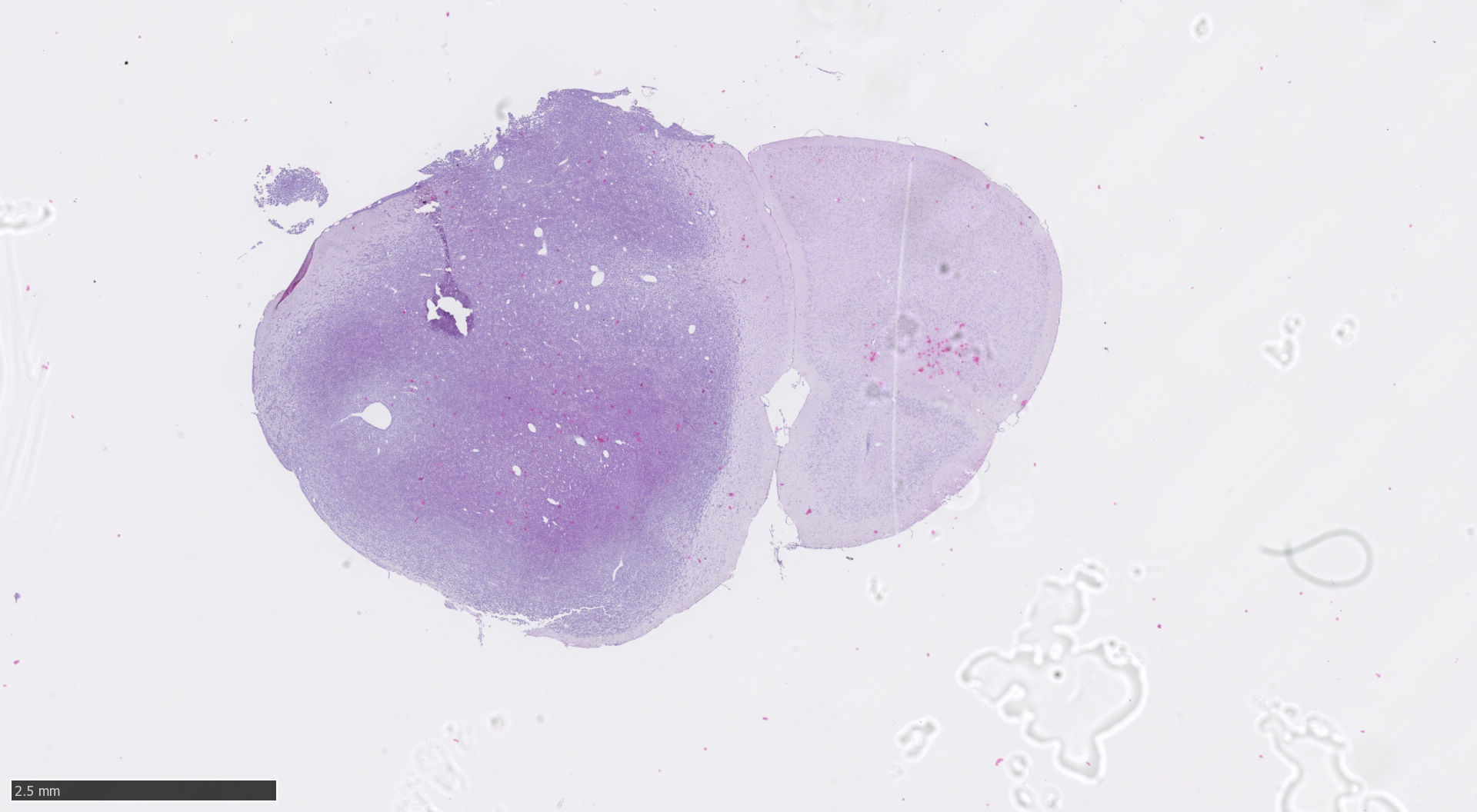

Supplement: Supplementary file 11 — Source data Fig. 5 [file 44321_2025_195_MOESM11_ESM.zip › Figure 5/5H/Vehicle control.jpg]

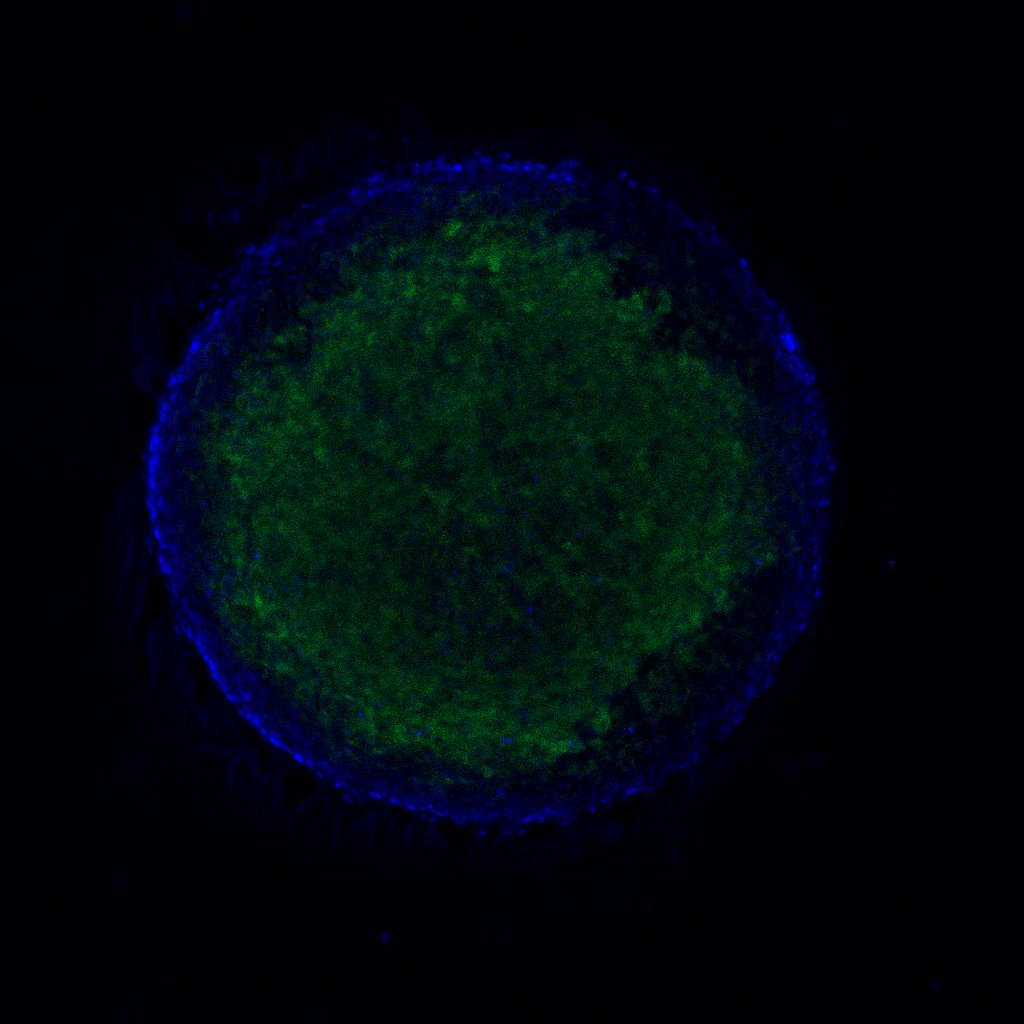

Supplement: Supplementary file 12 — Source data Fig. 6 [file 44321_2025_195_MOESM12_ESM.zip › Figure 6/6F/Figure 6F control normoxia higher exposure.jpg]

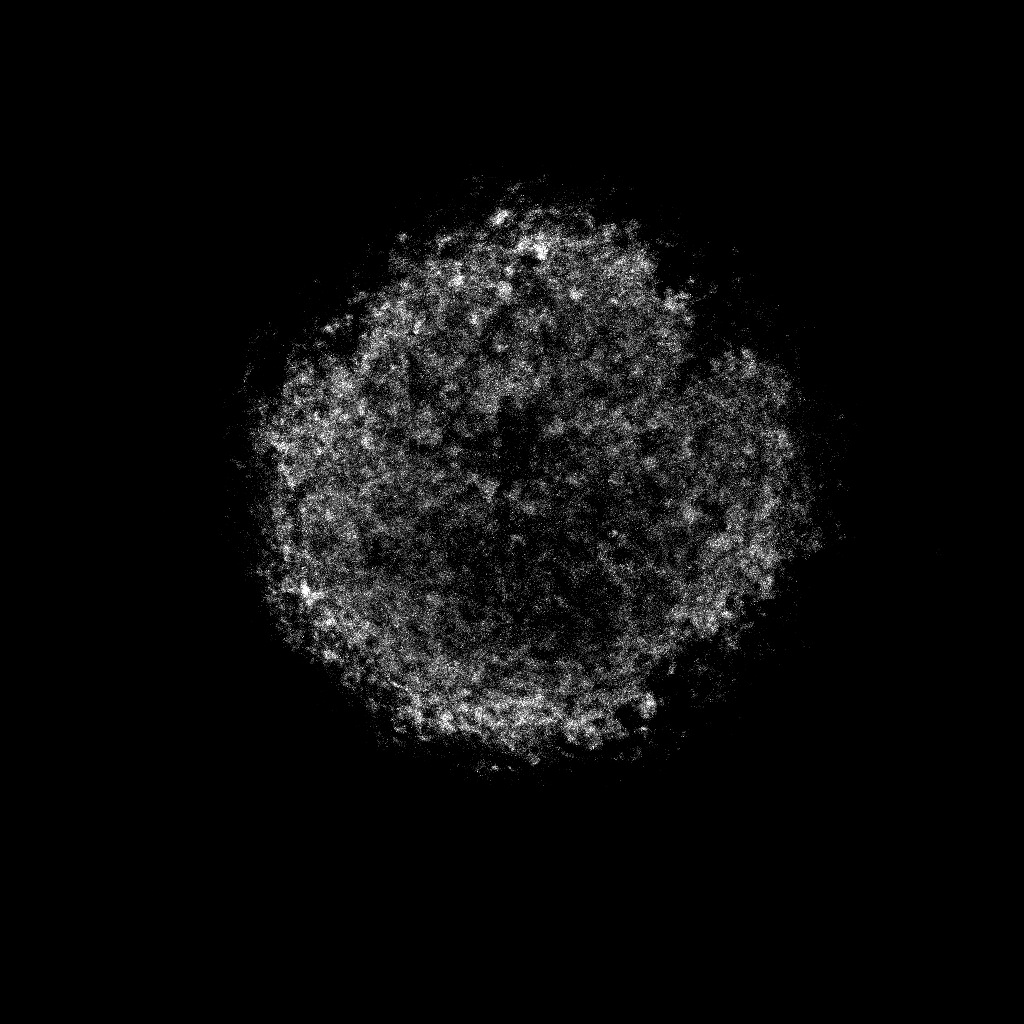

Supplement: Supplementary file 12 — Source data Fig. 6 [file 44321_2025_195_MOESM12_ESM.zip › Figure 6/6F/Figure 6F control normoxia.jpg]

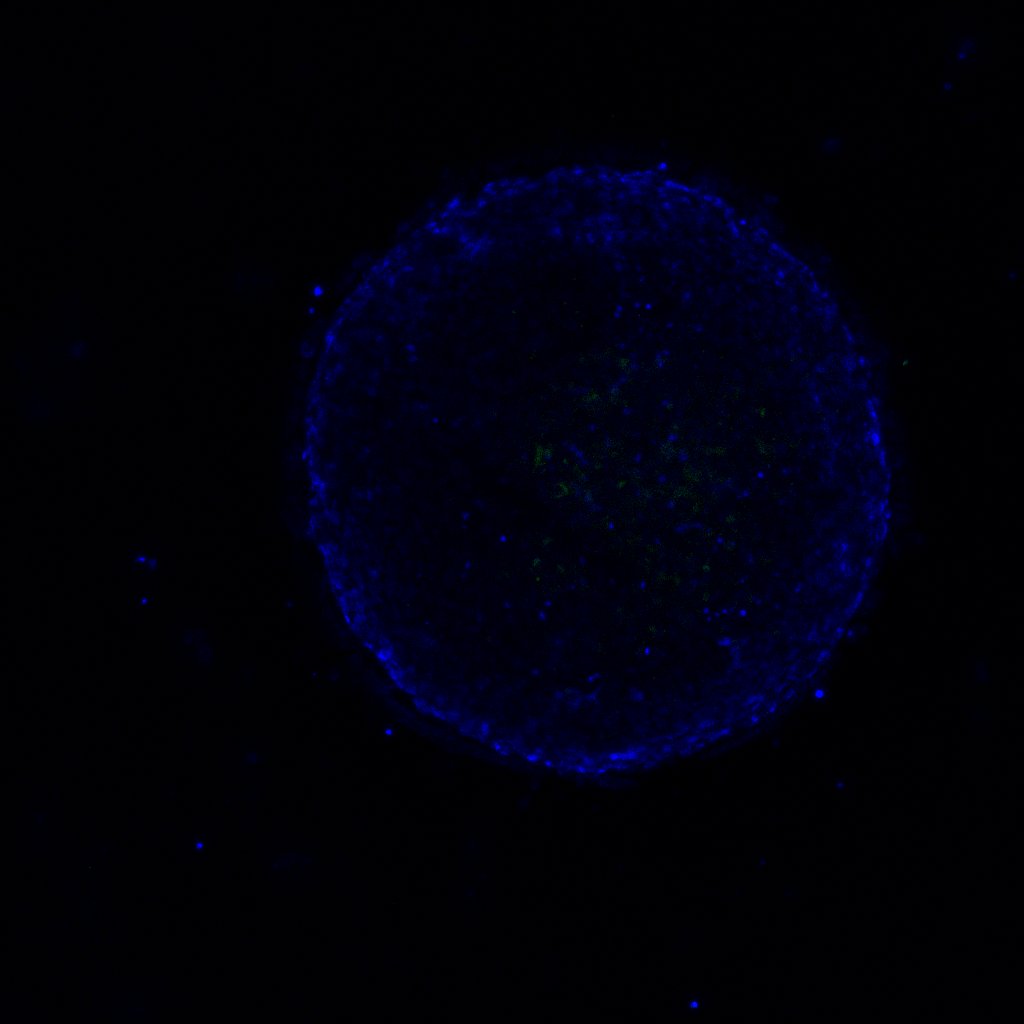

Supplement: Supplementary file 12 — Source data Fig. 6 [file 44321_2025_195_MOESM12_ESM.zip › Figure 6/6F/Figure 6F mubritinib normoxia higher exposure.jpg]

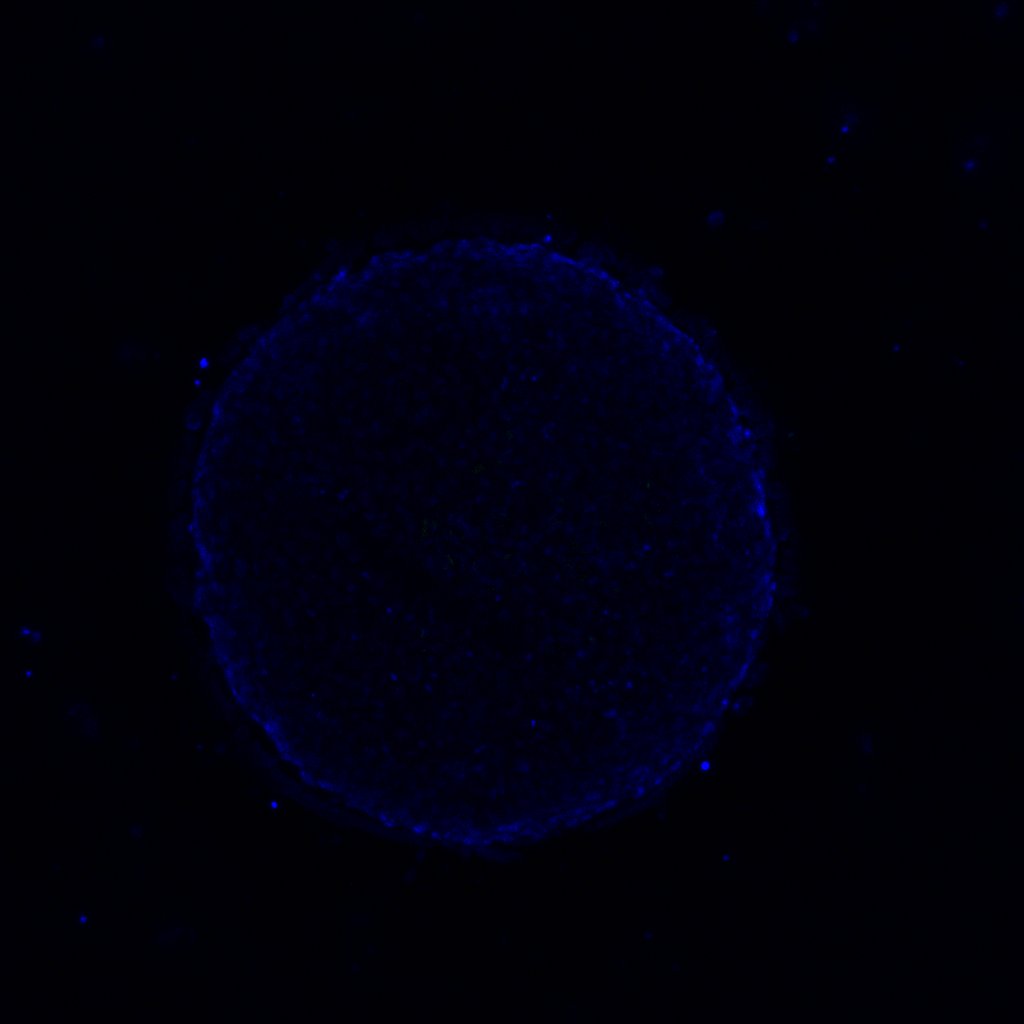

Supplement: Supplementary file 12 — Source data Fig. 6 [file 44321_2025_195_MOESM12_ESM.zip › Figure 6/6F/Figure 6F mubritinib normoxia.jpg]

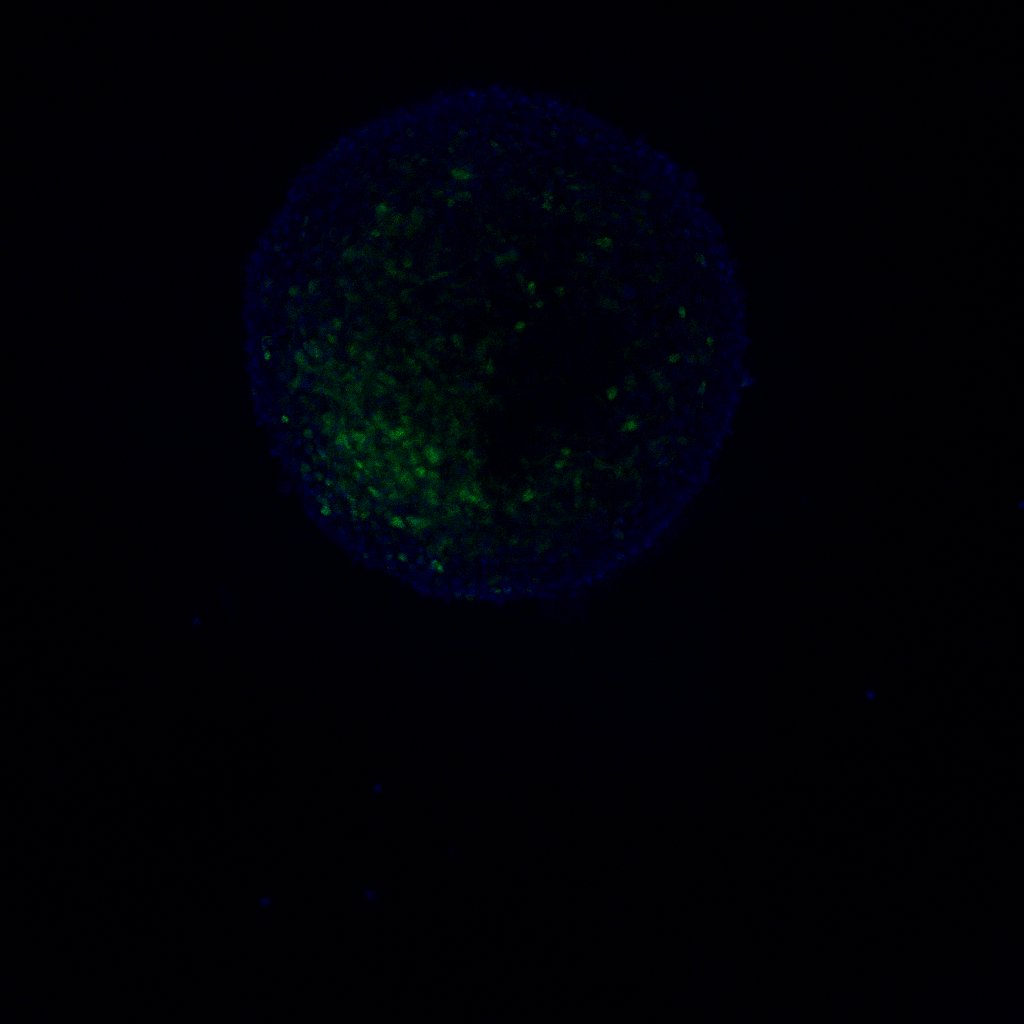

Supplement: Supplementary file 12 — Source data Fig. 6 [file 44321_2025_195_MOESM12_ESM.zip › Figure 6/6F/Figure 6F mubritinib hypoxia.jpg]

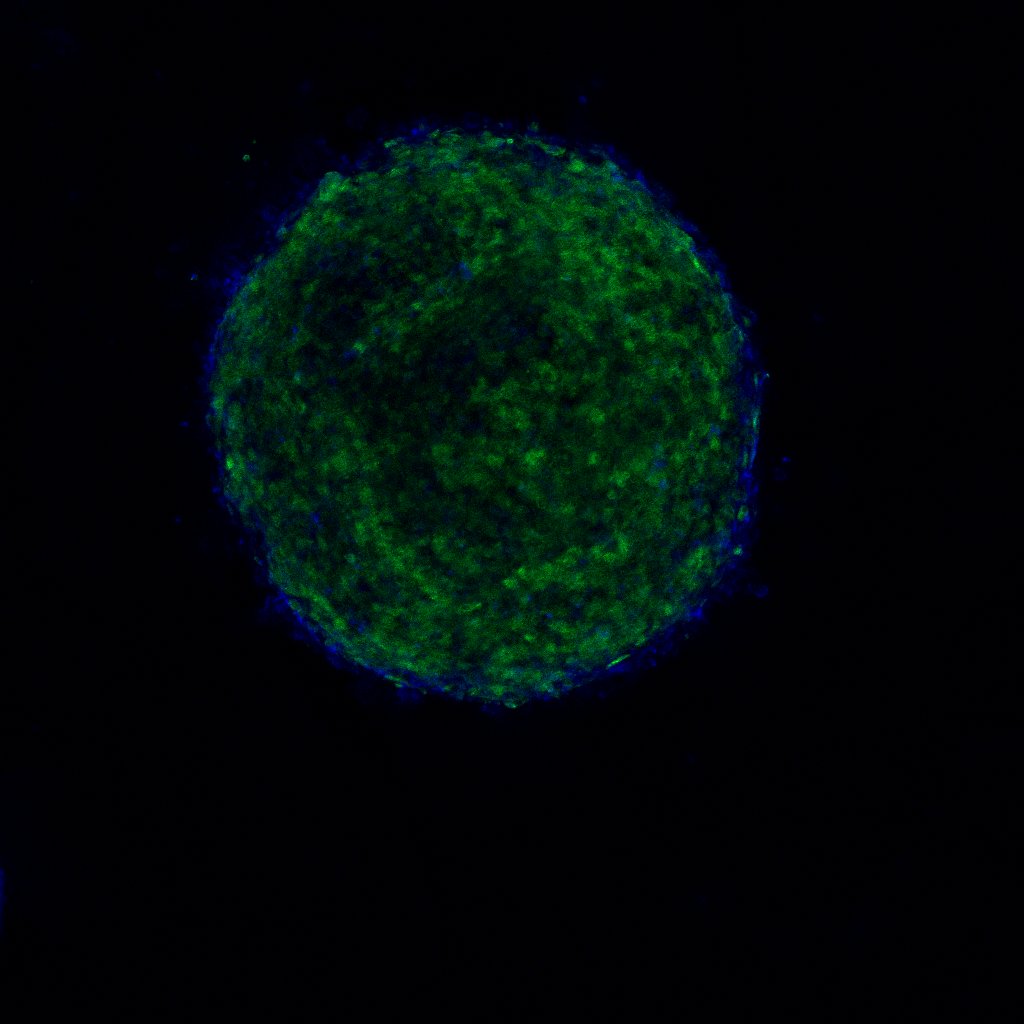

Supplement: Supplementary file 12 — Source data Fig. 6 [file 44321_2025_195_MOESM12_ESM.zip › Figure 6/6F/Figure 6F control hypoxia.jpg]

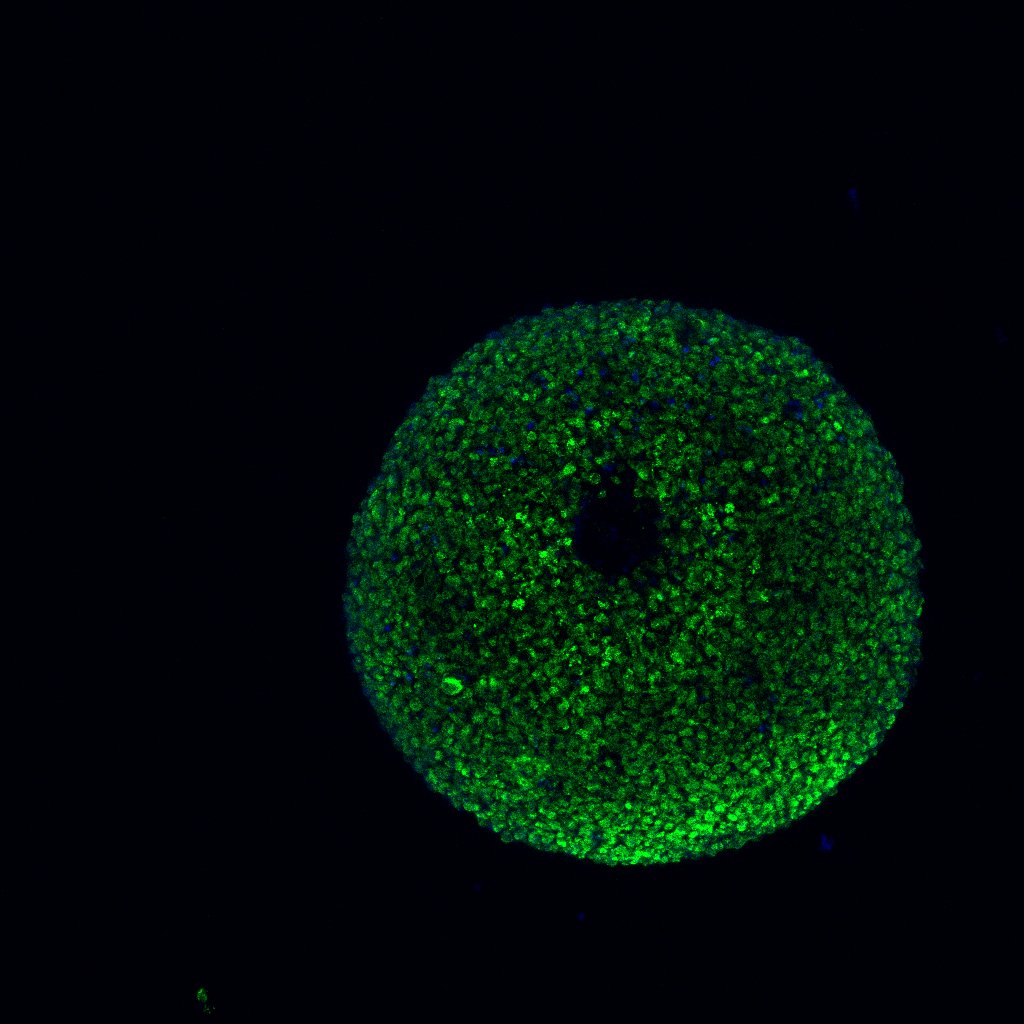

Supplement: Supplementary file 12 — Source data Fig. 6 [file 44321_2025_195_MOESM12_ESM.zip › Figure 6/6A/Figure 6A hypoxia control.jpg]

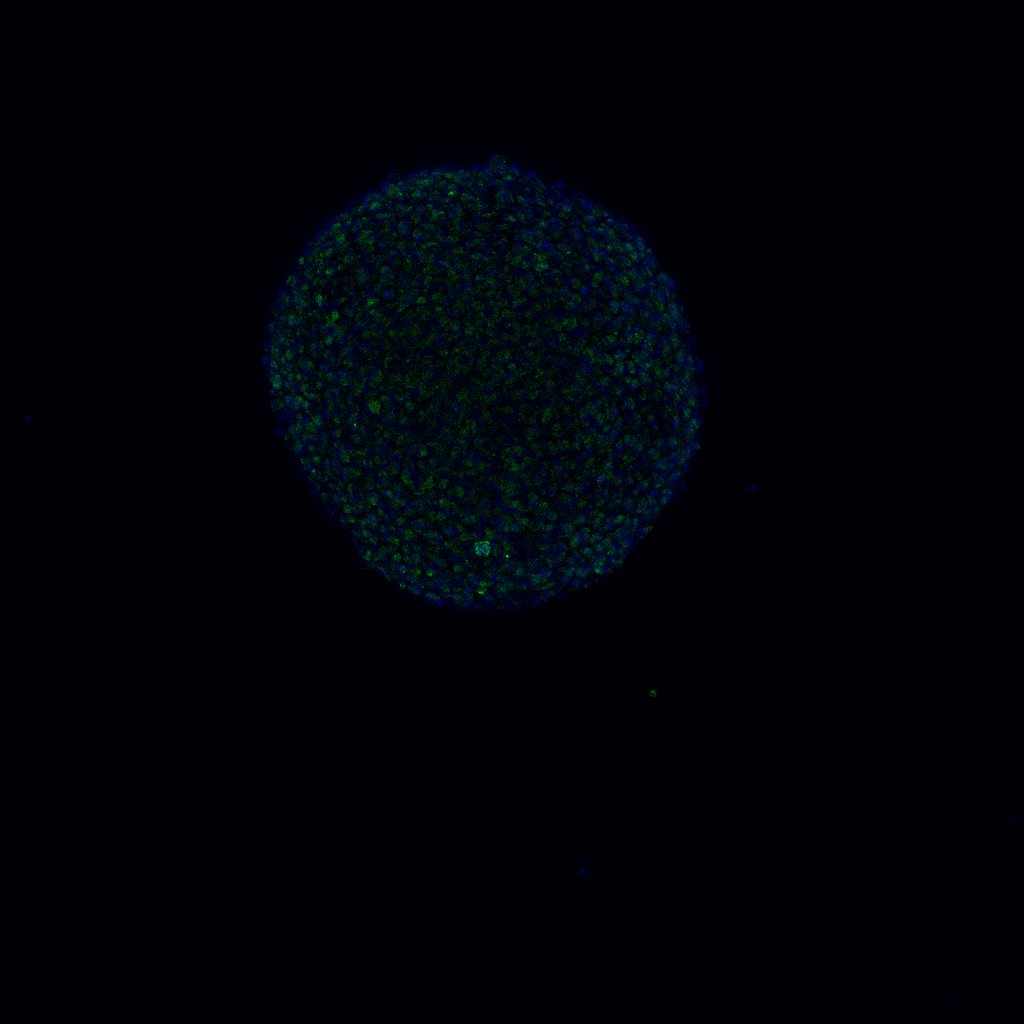

Supplement: Supplementary file 12 — Source data Fig. 6 [file 44321_2025_195_MOESM12_ESM.zip › Figure 6/6A/Figure 6A hypoxia mubritinib.jpg]

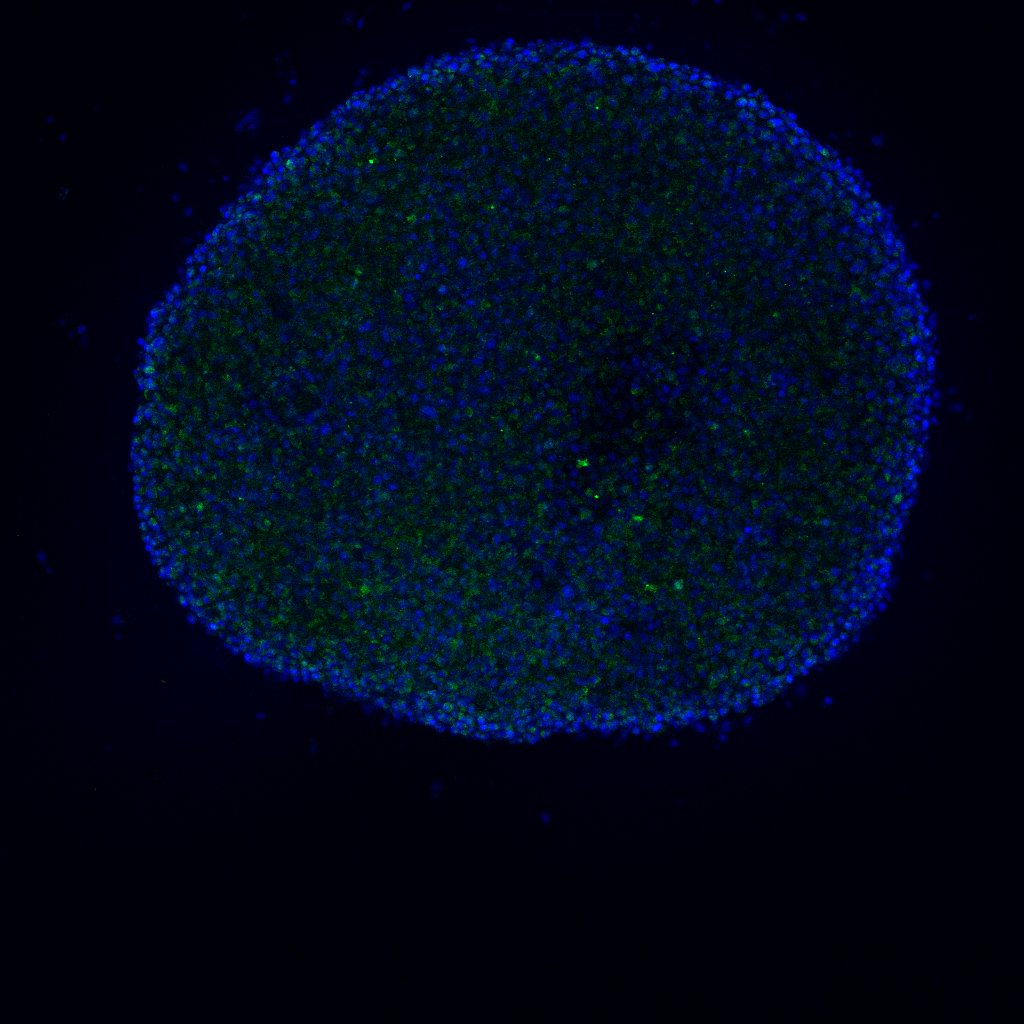

Supplement: Supplementary file 12 — Source data Fig. 6 [file 44321_2025_195_MOESM12_ESM.zip › Figure 6/6A/Figure 6A normoxia mubritinib.jpg]

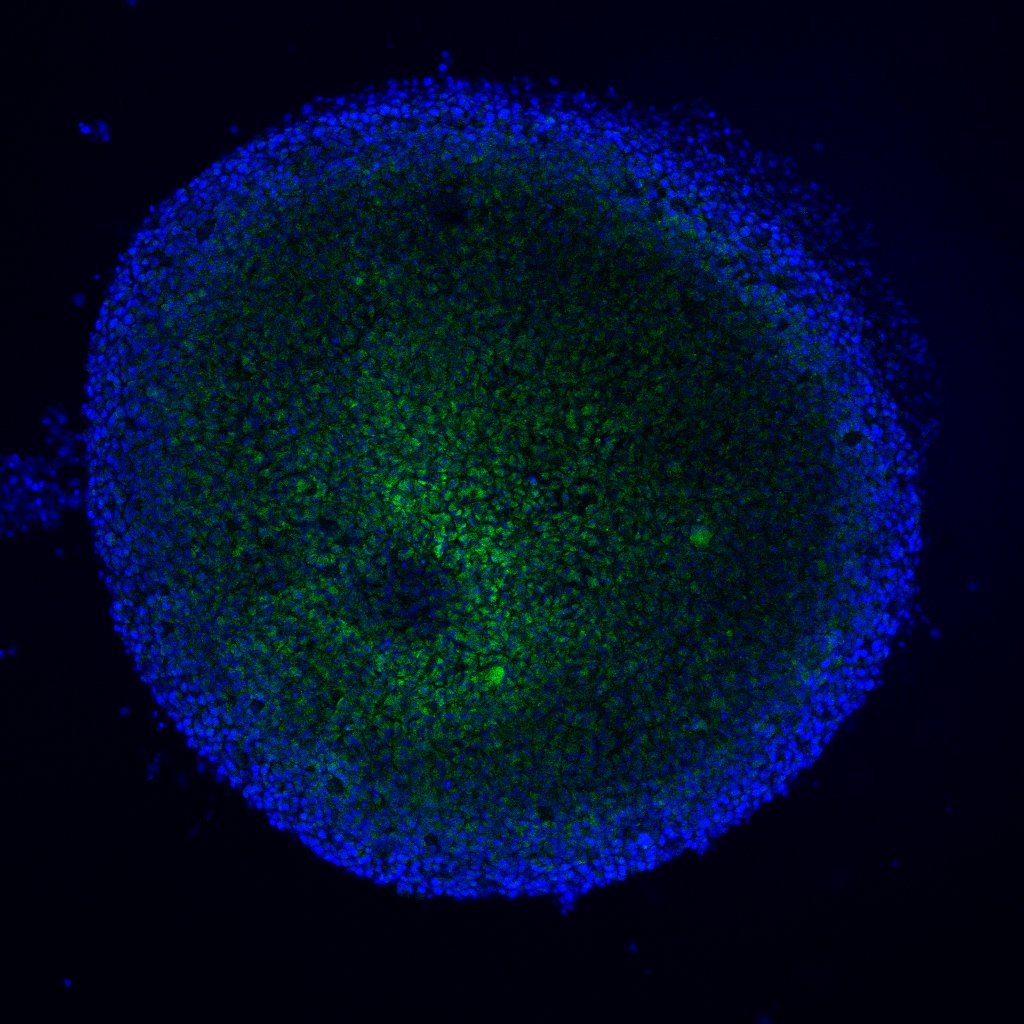

Supplement: Supplementary file 12 — Source data Fig. 6 [file 44321_2025_195_MOESM12_ESM.zip › Figure 6/6A/Figure 6A normoxia control.jpg]

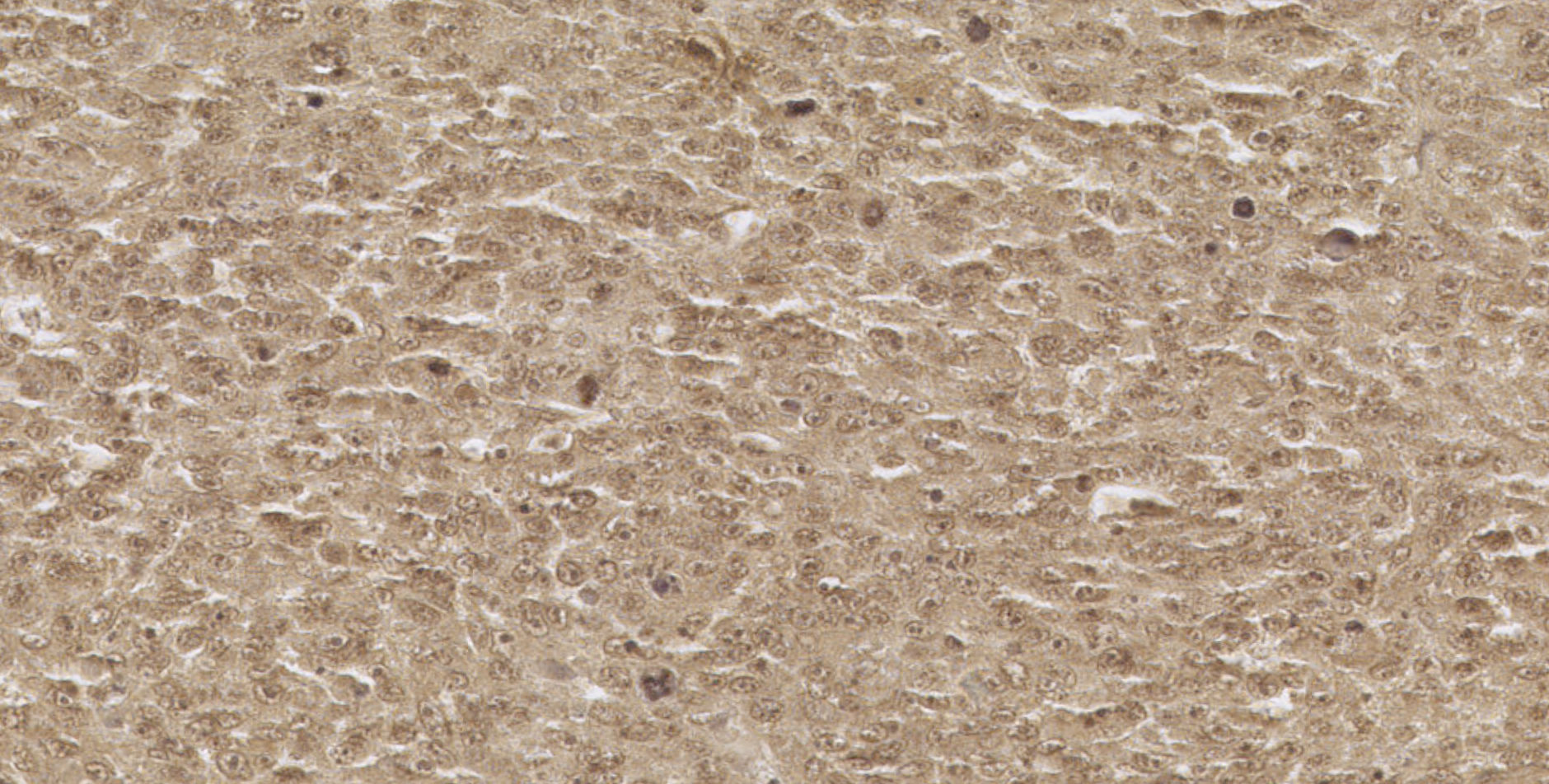

Supplement: Supplementary file 12 — Source data Fig. 6 [file 44321_2025_195_MOESM12_ESM.zip › Figure 6/6N/Figure 6N IR.jpg]

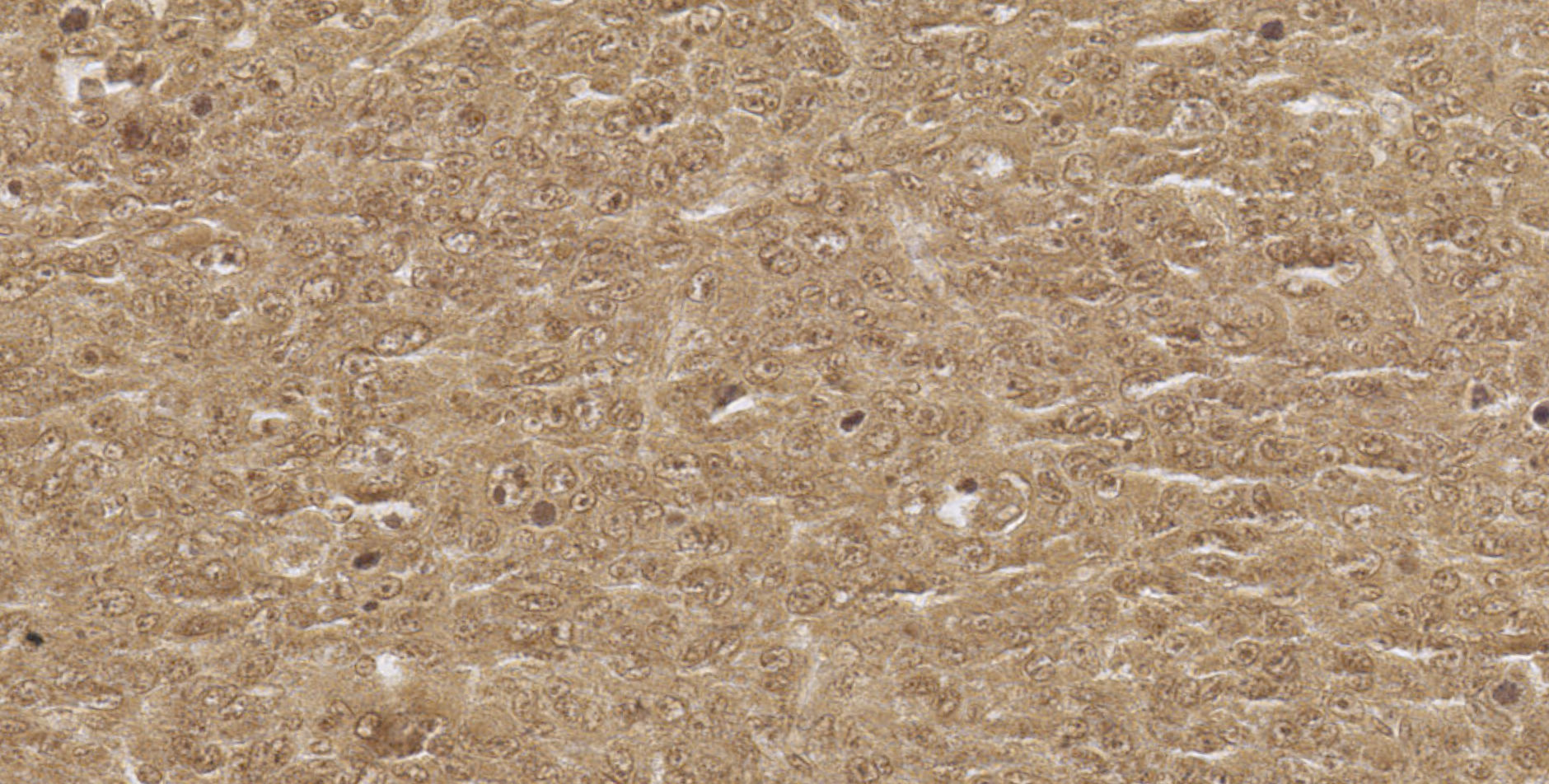

Supplement: Supplementary file 12 — Source data Fig. 6 [file 44321_2025_195_MOESM12_ESM.zip › Figure 6/6N/Figure 6N mubritinib + IR.jpg]

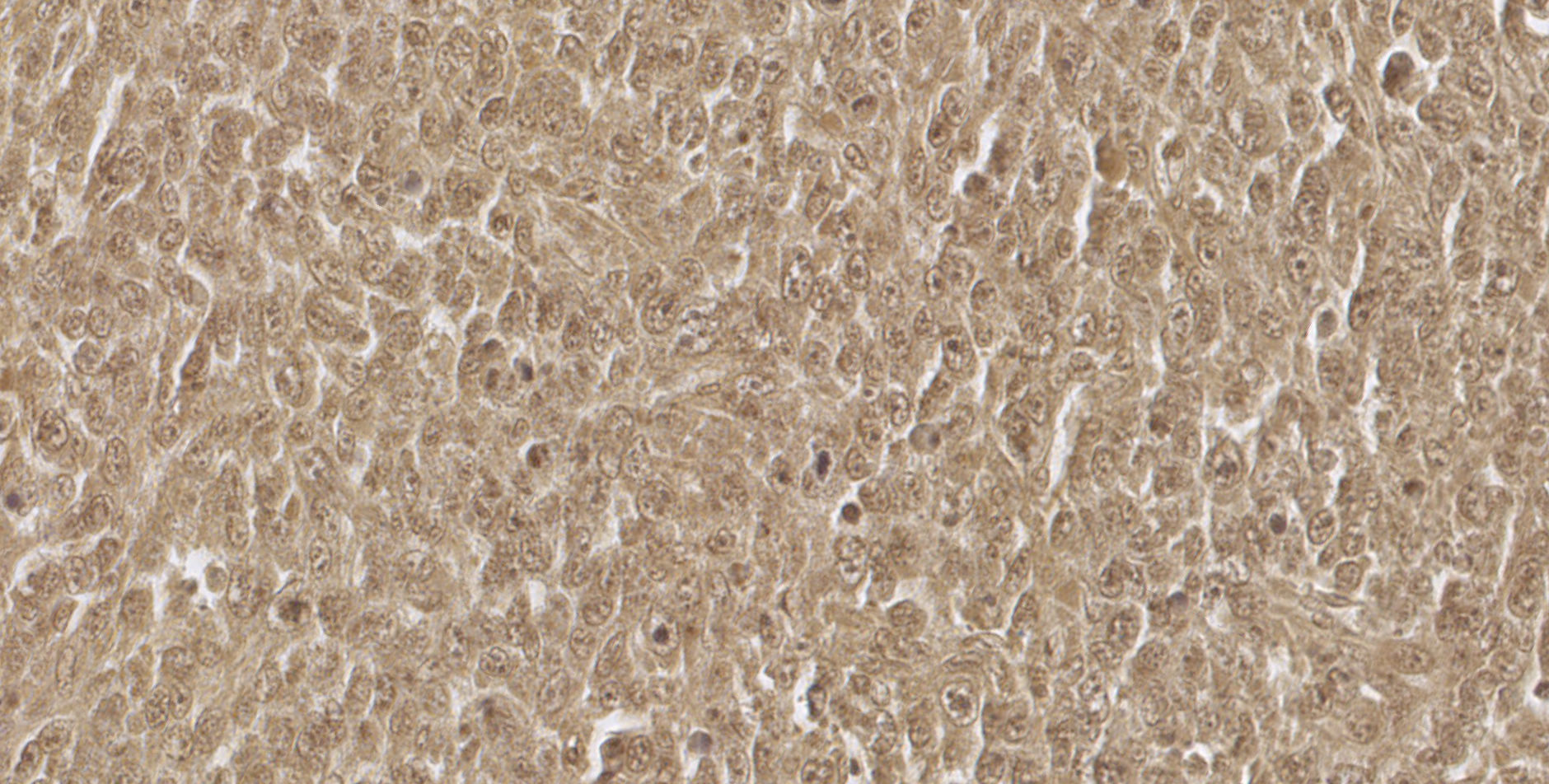

Supplement: Supplementary file 12 — Source data Fig. 6 [file 44321_2025_195_MOESM12_ESM.zip › Figure 6/6N/Figure 6N mubritinib.jpg]

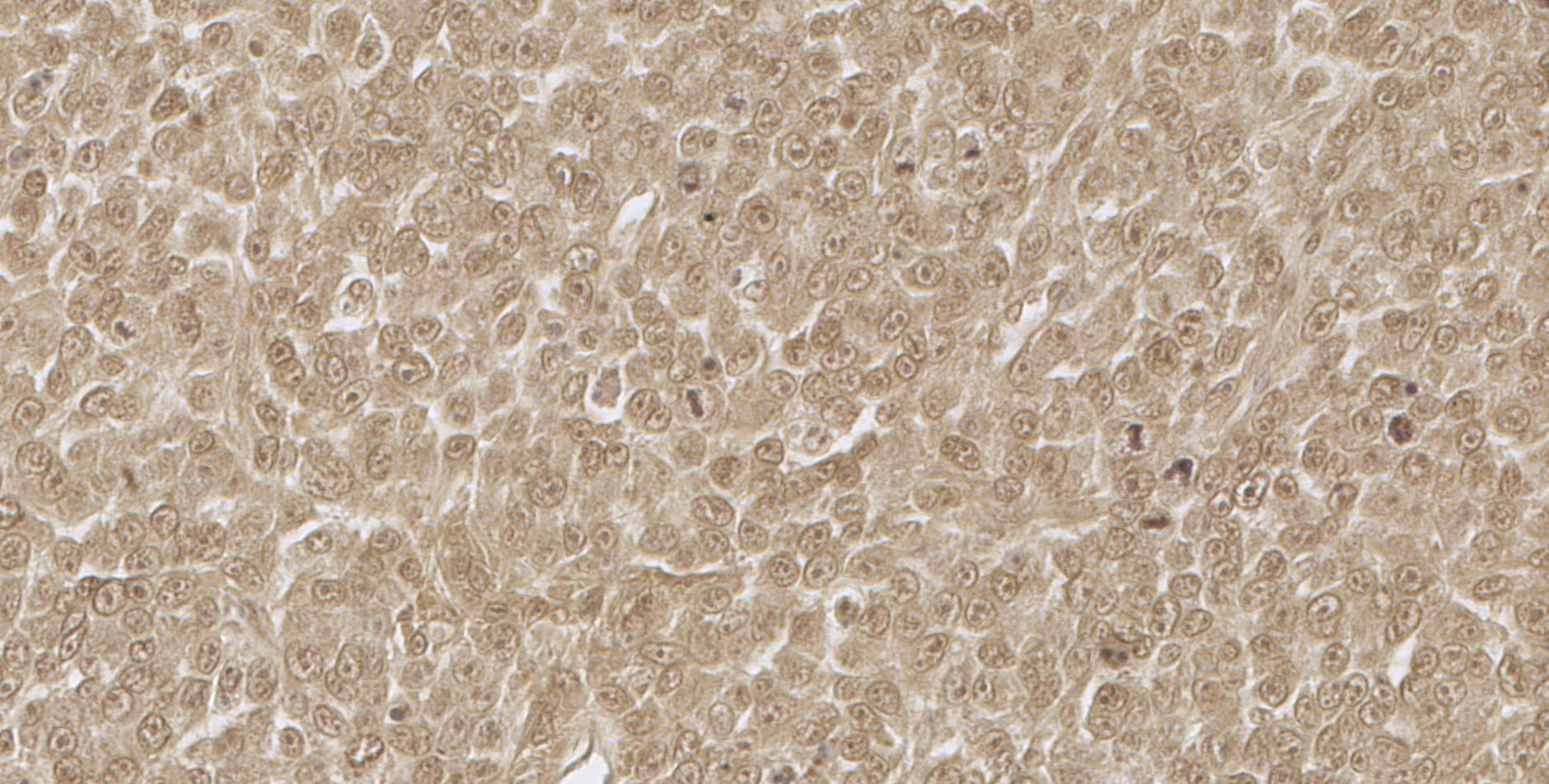

Supplement: Supplementary file 12 — Source data Fig. 6 [file 44321_2025_195_MOESM12_ESM.zip › Figure 6/6N/Figure 6N Vehicle.jpg]

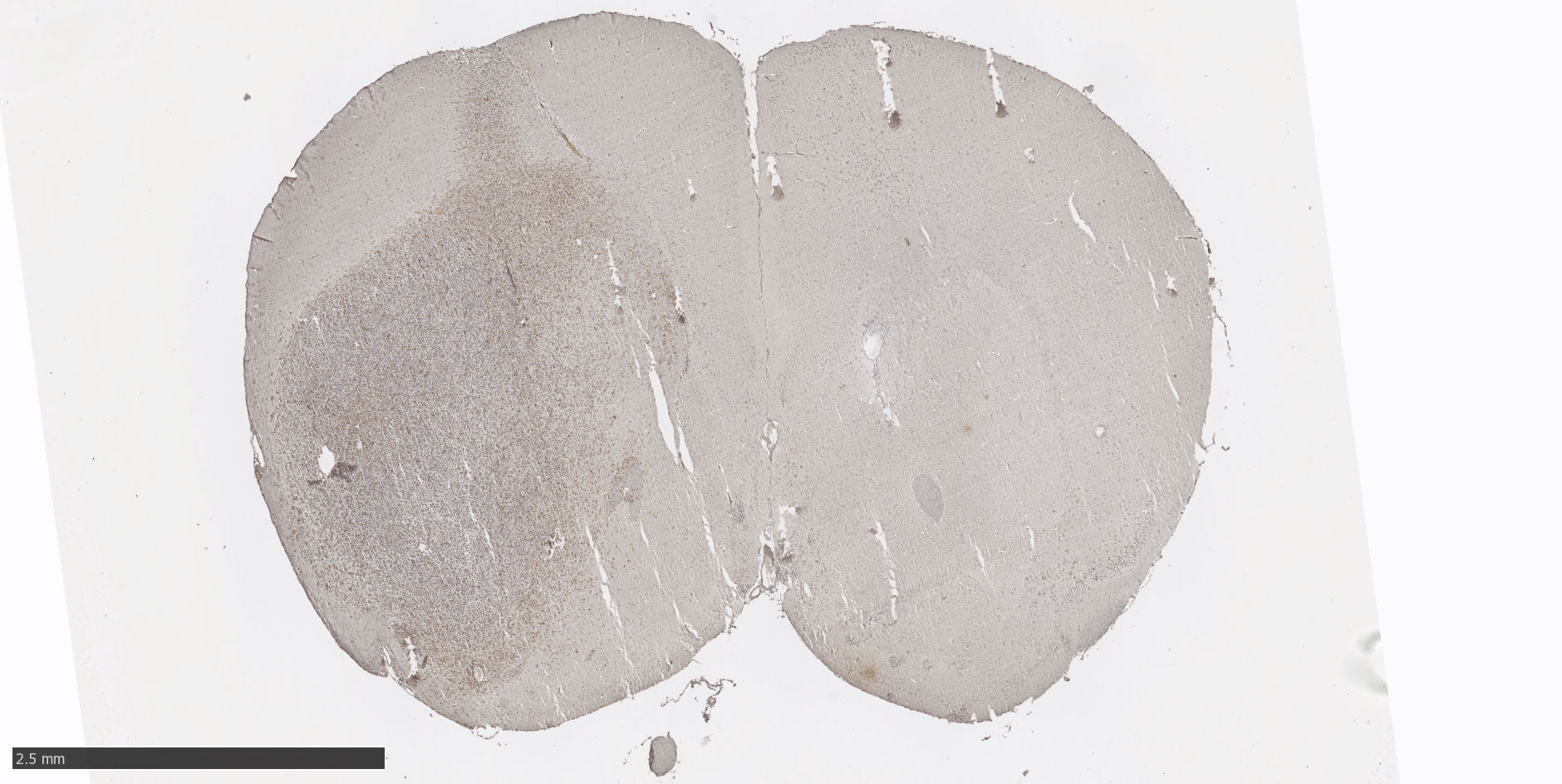

Supplement: Supplementary file 12 — Source data Fig. 6 [file 44321_2025_195_MOESM12_ESM.zip › Figure 6/6I/Figure 6I Vehicle control.jpg]

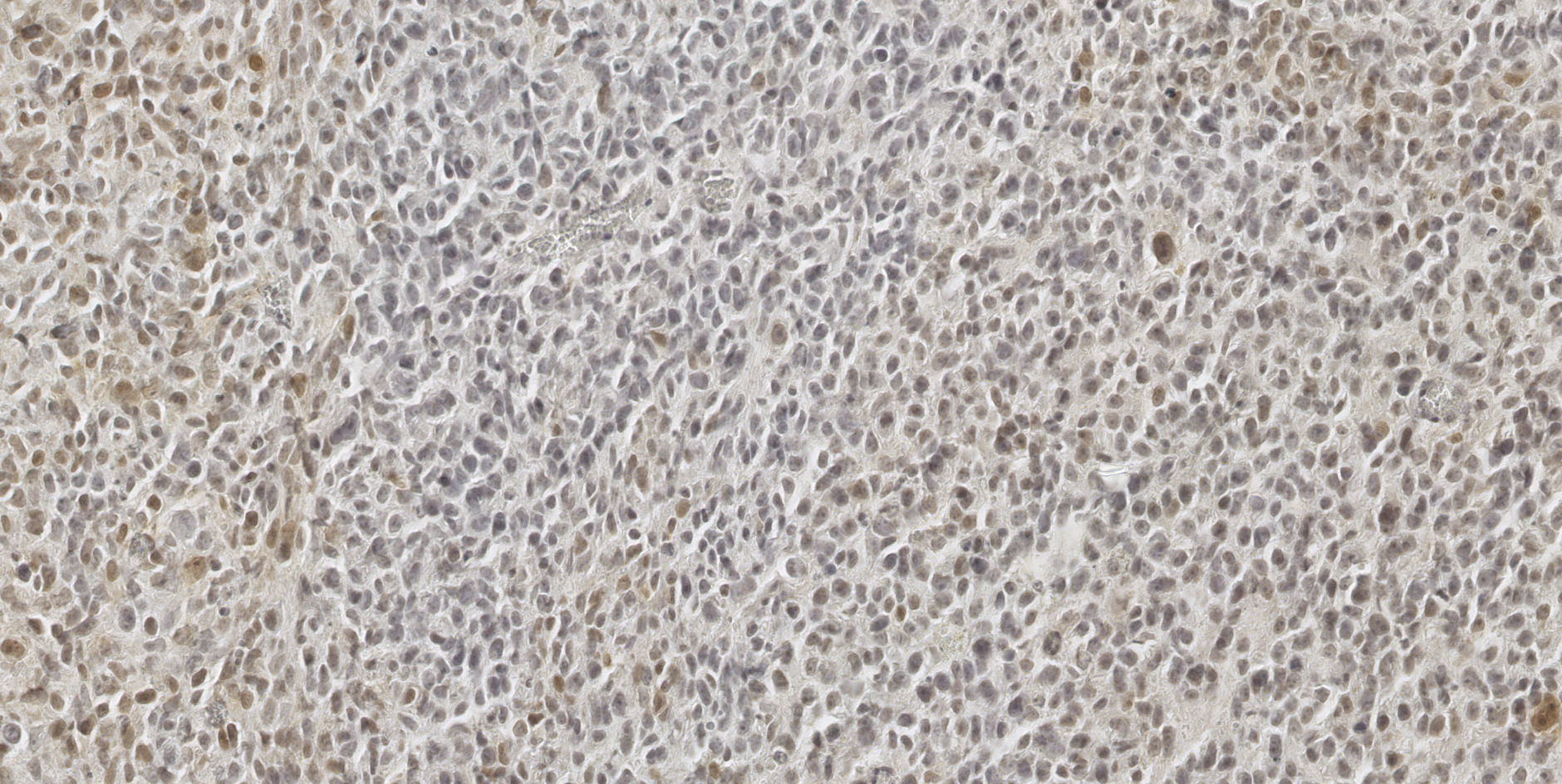

Supplement: Supplementary file 12 — Source data Fig. 6 [file 44321_2025_195_MOESM12_ESM.zip › Figure 6/6I/Figure 6I mubritinib inset 4.jpg]

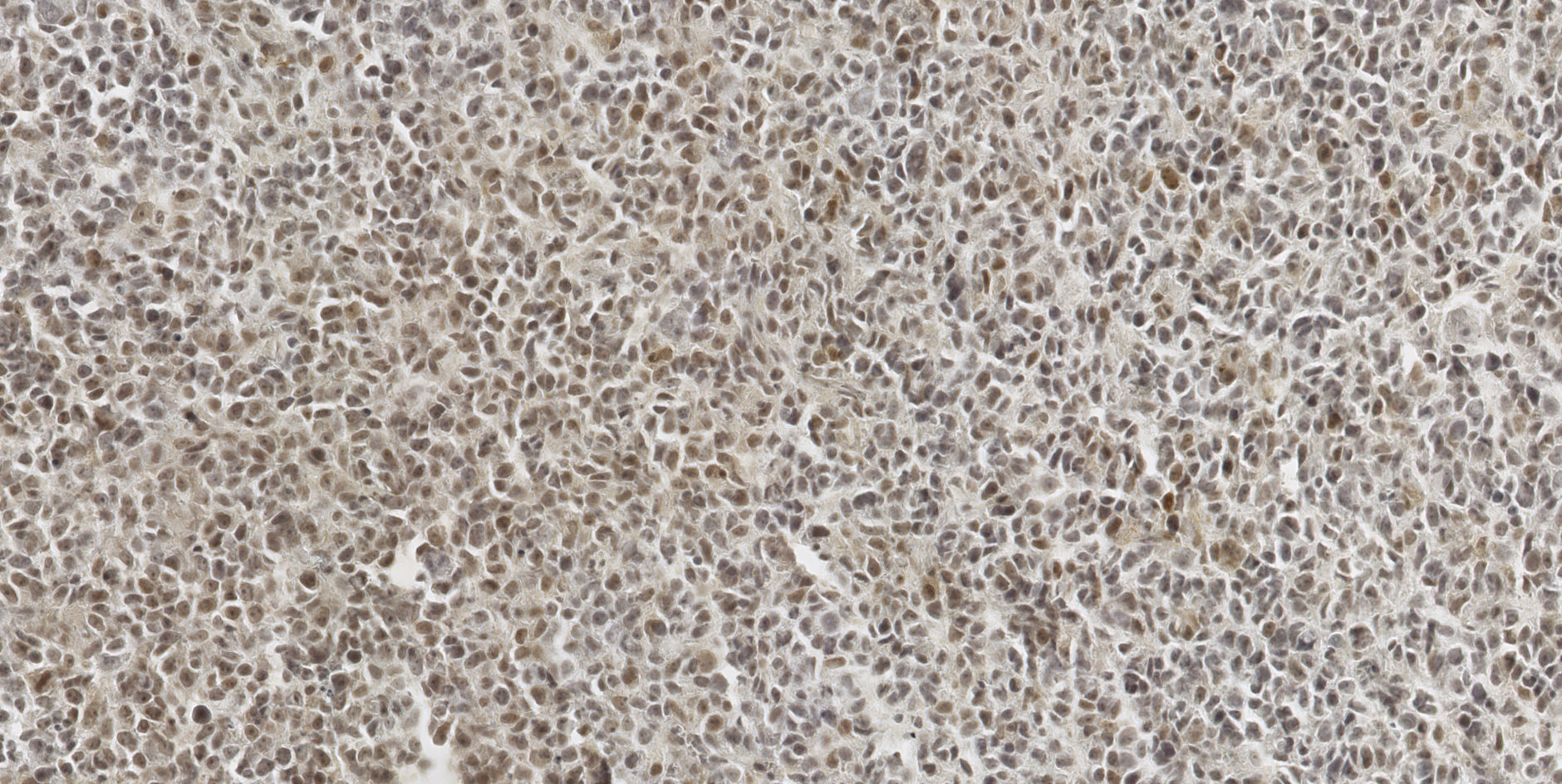

Supplement: Supplementary file 12 — Source data Fig. 6 [file 44321_2025_195_MOESM12_ESM.zip › Figure 6/6I/Figure 6I Vehicle control inset 2.jpg]

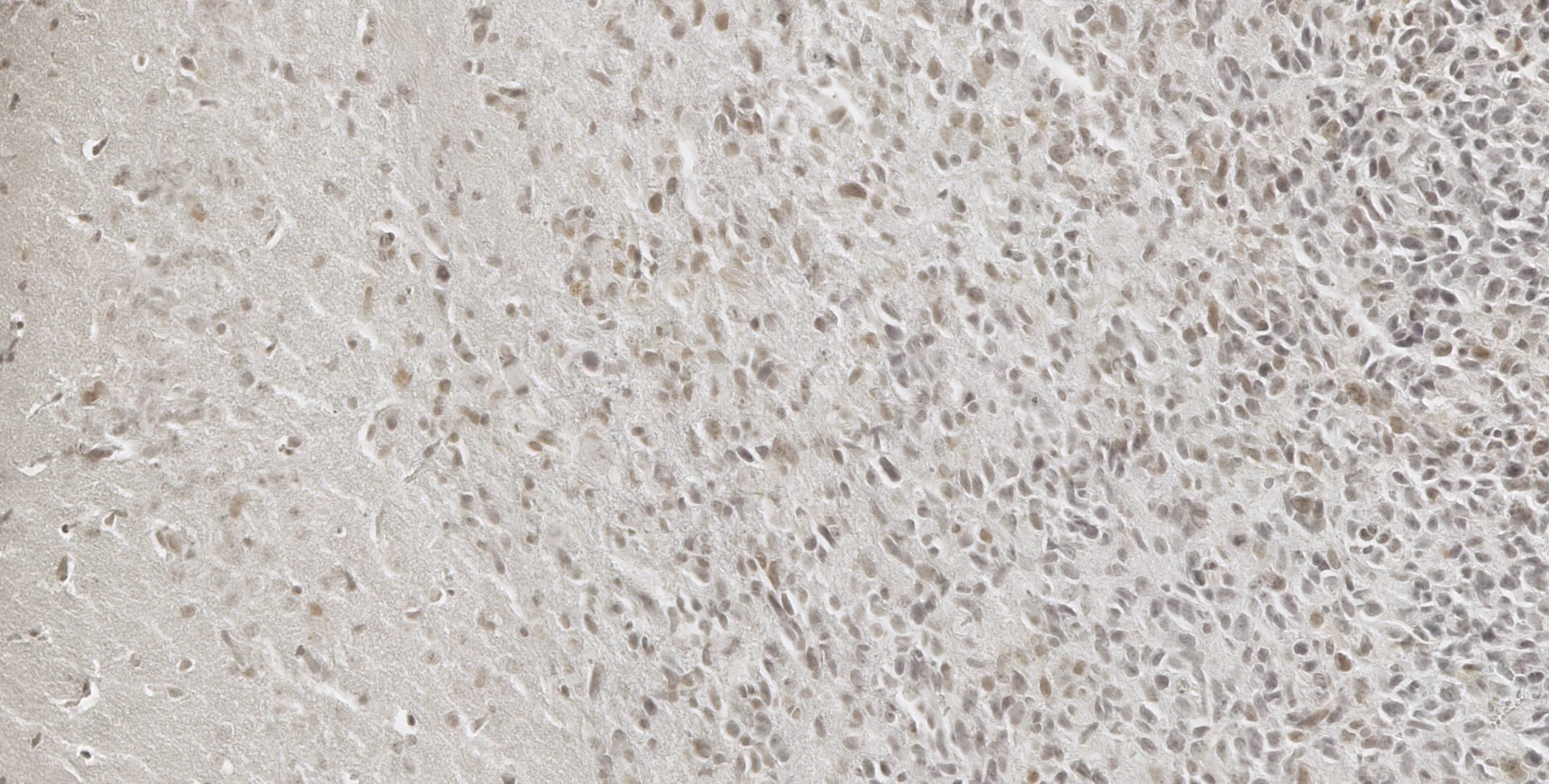

Supplement: Supplementary file 12 — Source data Fig. 6 [file 44321_2025_195_MOESM12_ESM.zip › Figure 6/6I/Figure 6I mubritinib inset 3.jpg]

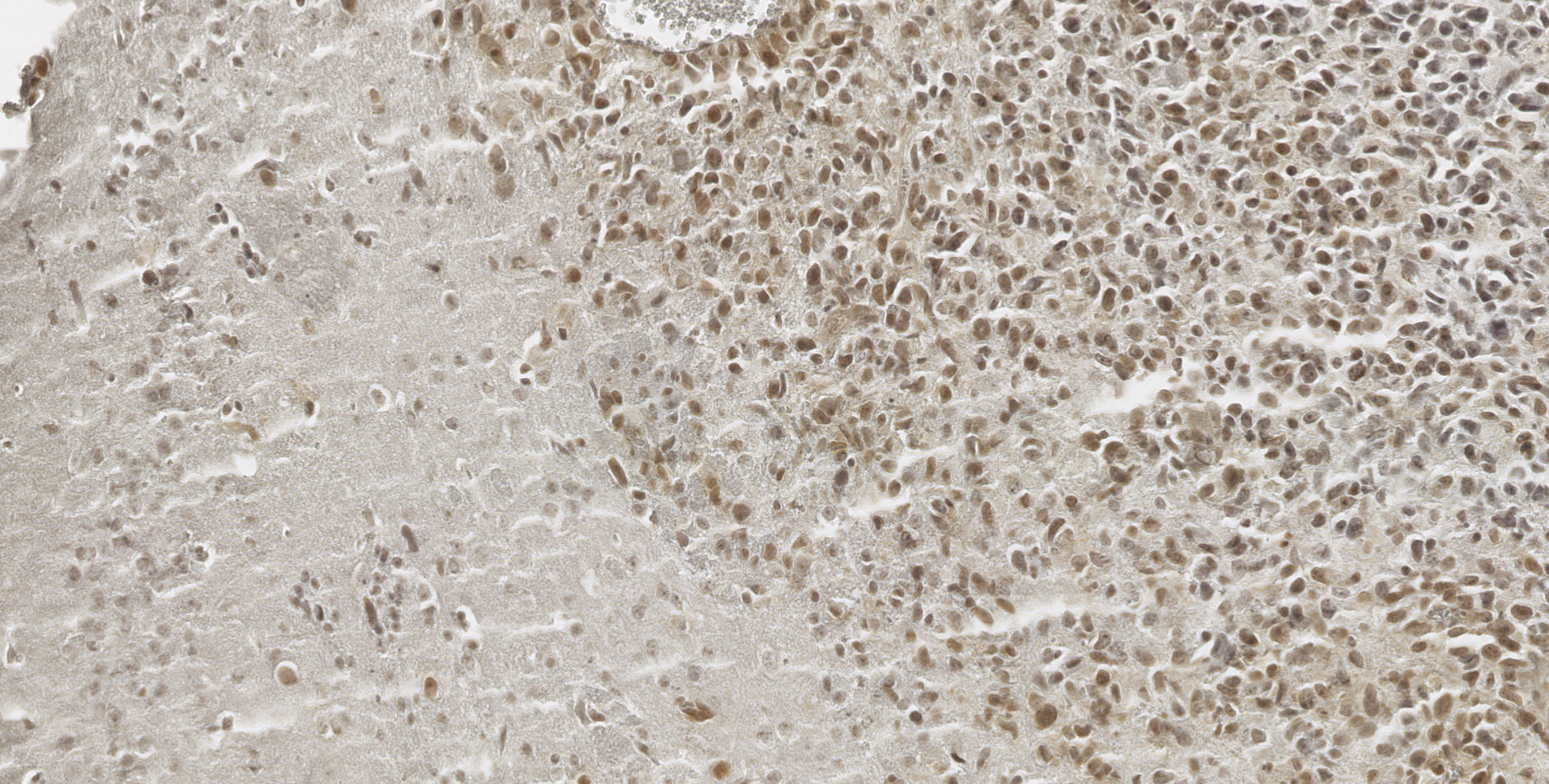

Supplement: Supplementary file 12 — Source data Fig. 6 [file 44321_2025_195_MOESM12_ESM.zip › Figure 6/6I/Figure 6I vehicle control inset 1.jpg]

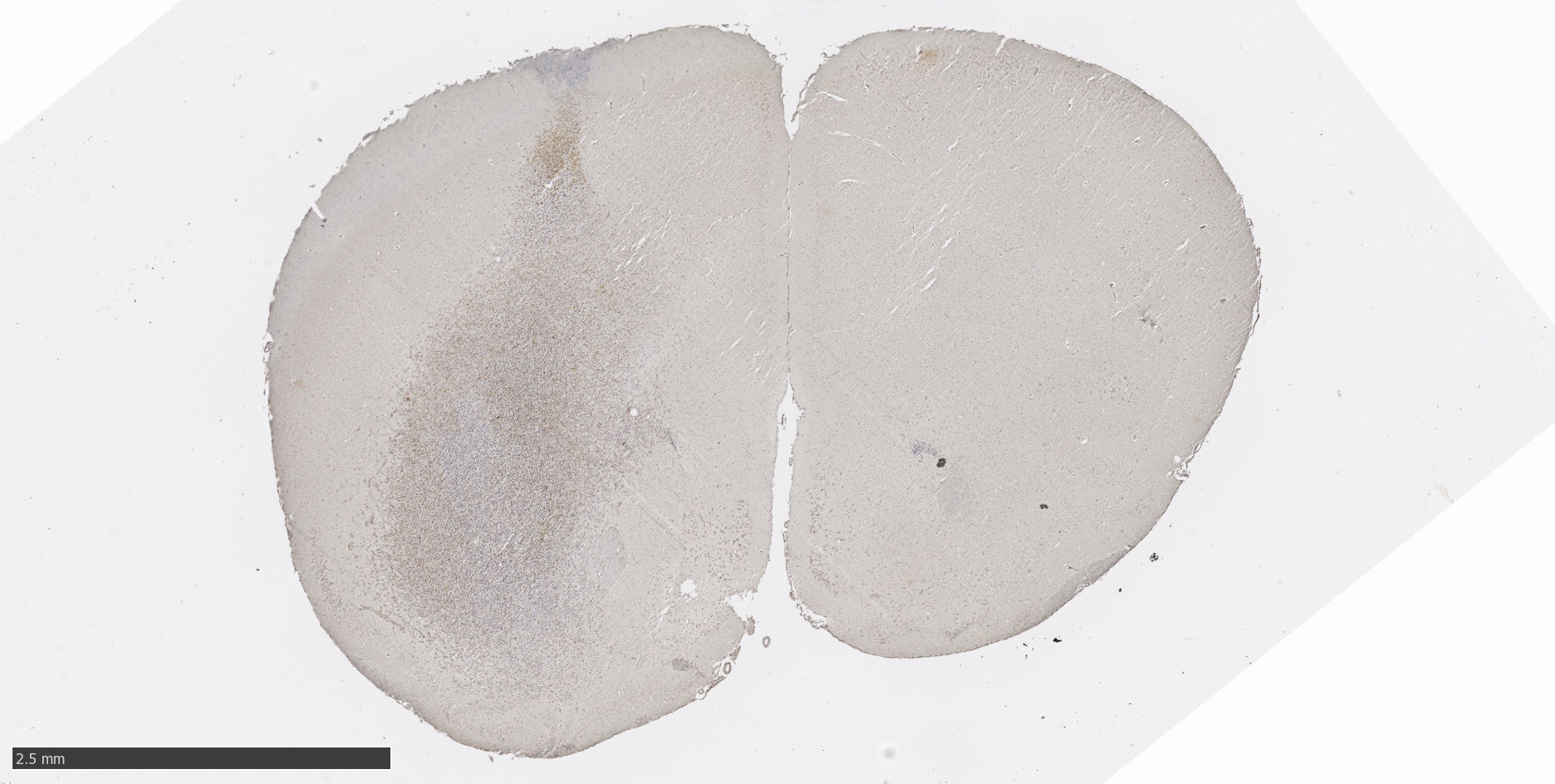

Supplement: Supplementary file 12 — Source data Fig. 6 [file 44321_2025_195_MOESM12_ESM.zip › Figure 6/6I/Figure 6I mubritinib.jpg]

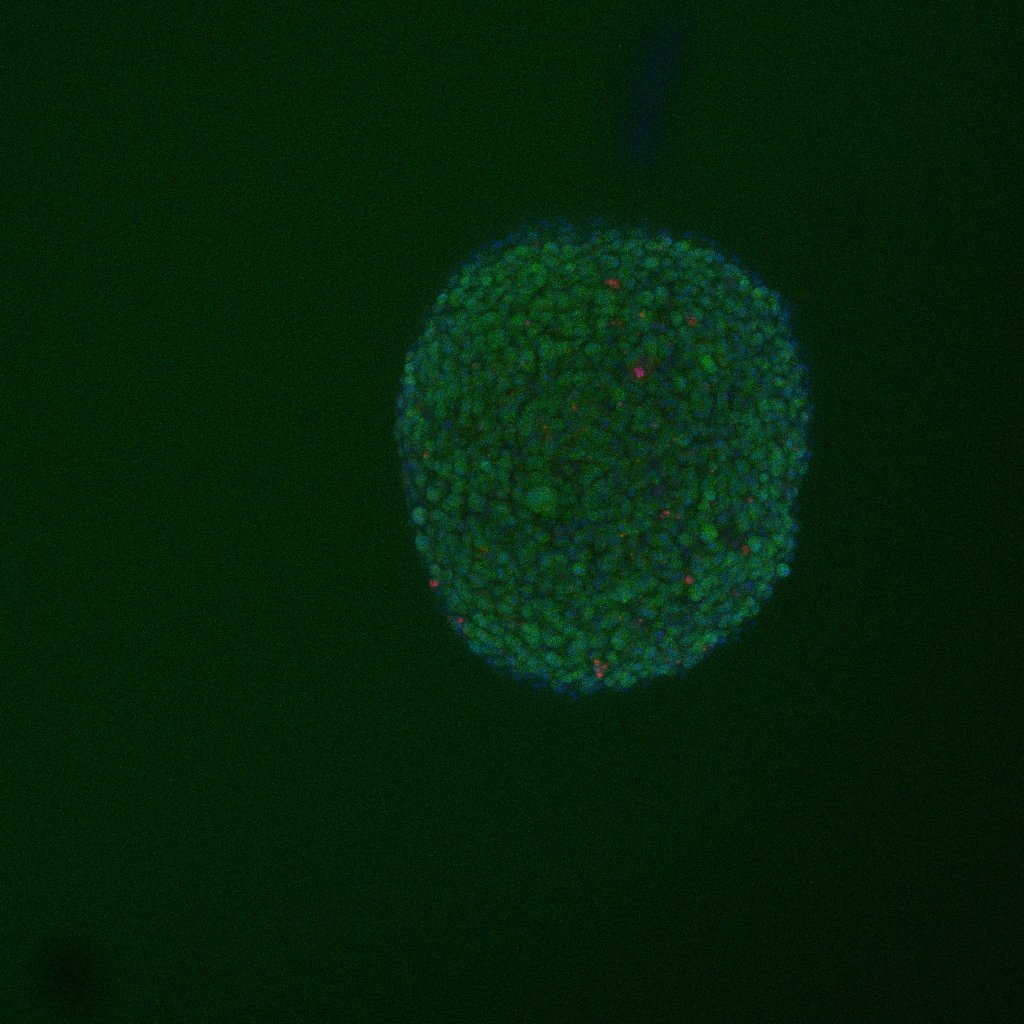

Supplement: Supplementary file 12 — Source data Fig. 6 [file 44321_2025_195_MOESM12_ESM.zip › Figure 6/6L/Figure 6L H2DCFDA MitoSOX DAPI mubritinib.jpg]

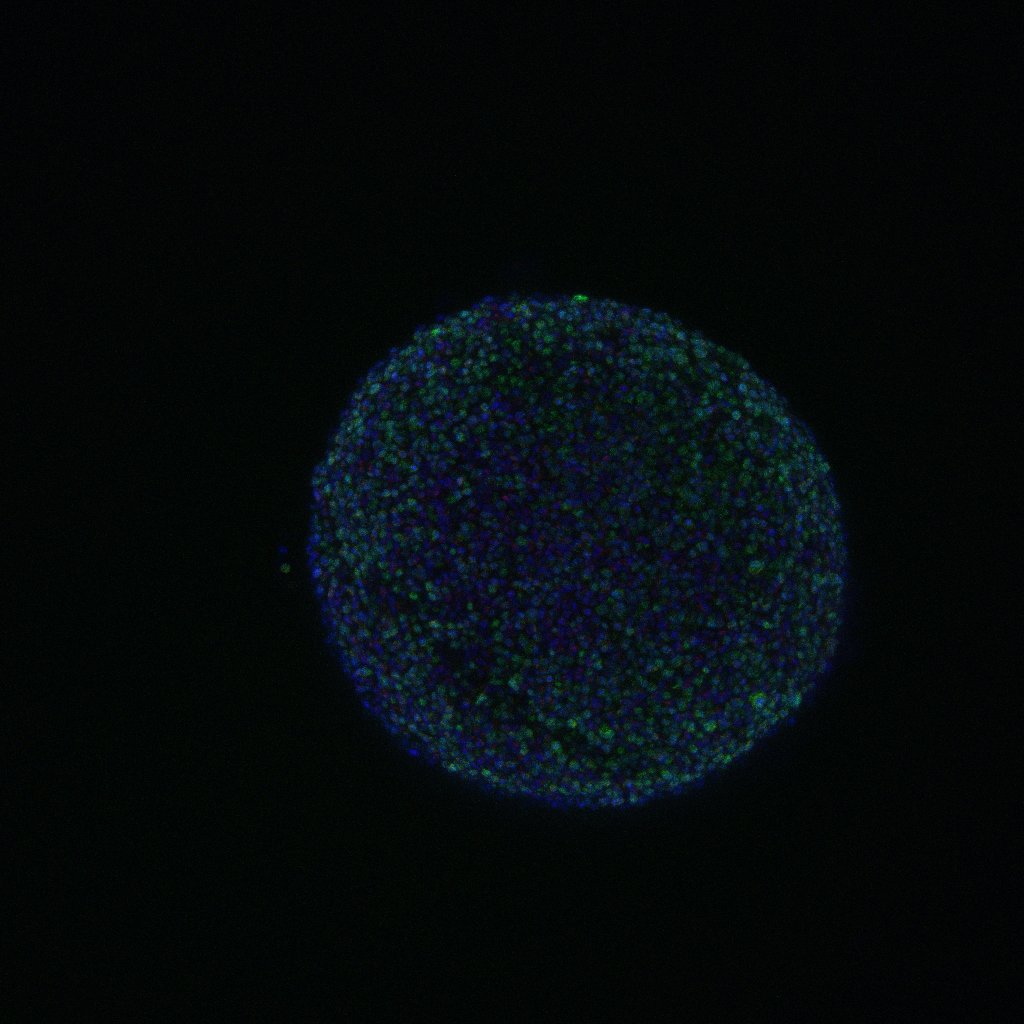

Supplement: Supplementary file 12 — Source data Fig. 6 [file 44321_2025_195_MOESM12_ESM.zip › Figure 6/6L/Figure 6L H2DCFDA MitoSOX DAPI Control.jpg]

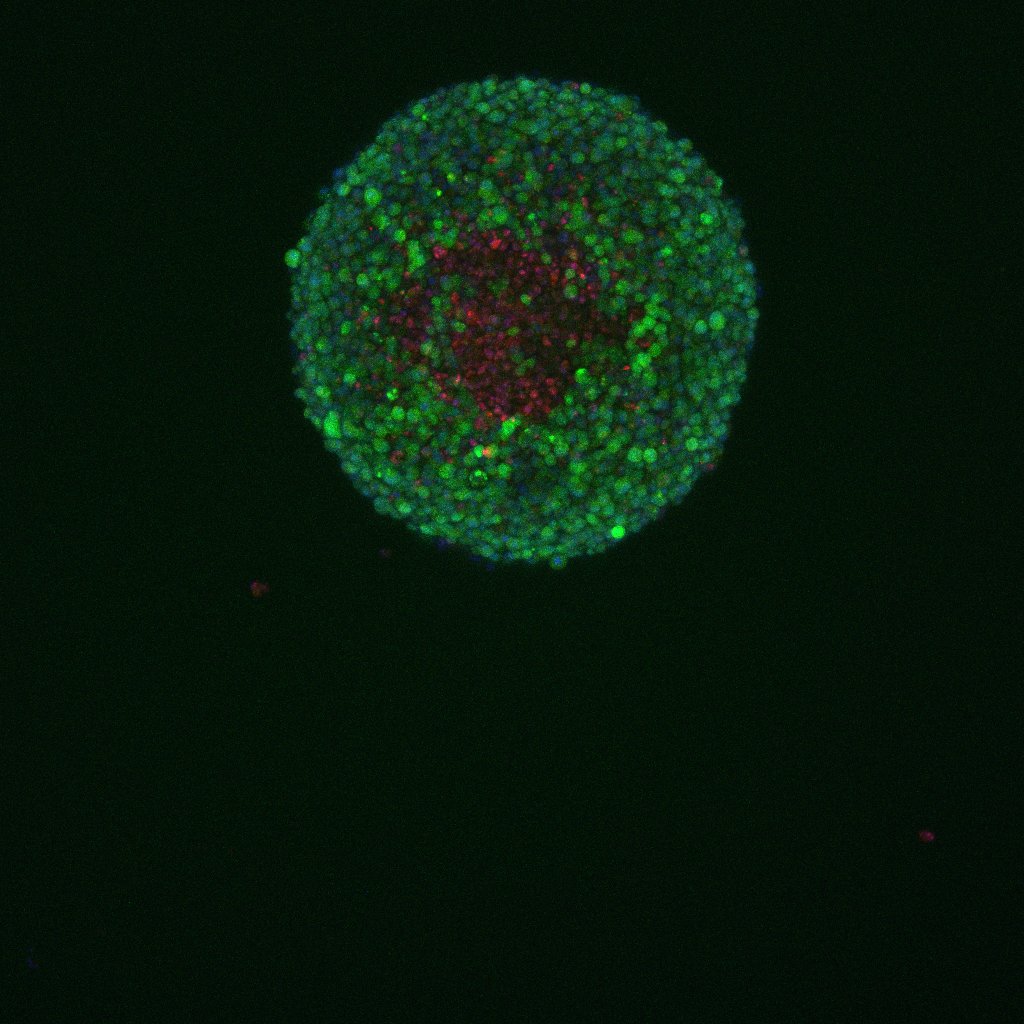

Supplement: Supplementary file 12 — Source data Fig. 6 [file 44321_2025_195_MOESM12_ESM.zip › Figure 6/6L/Figure 6L H2DCFDA MitoSOX DAPI mubritinib + IR.jpg]

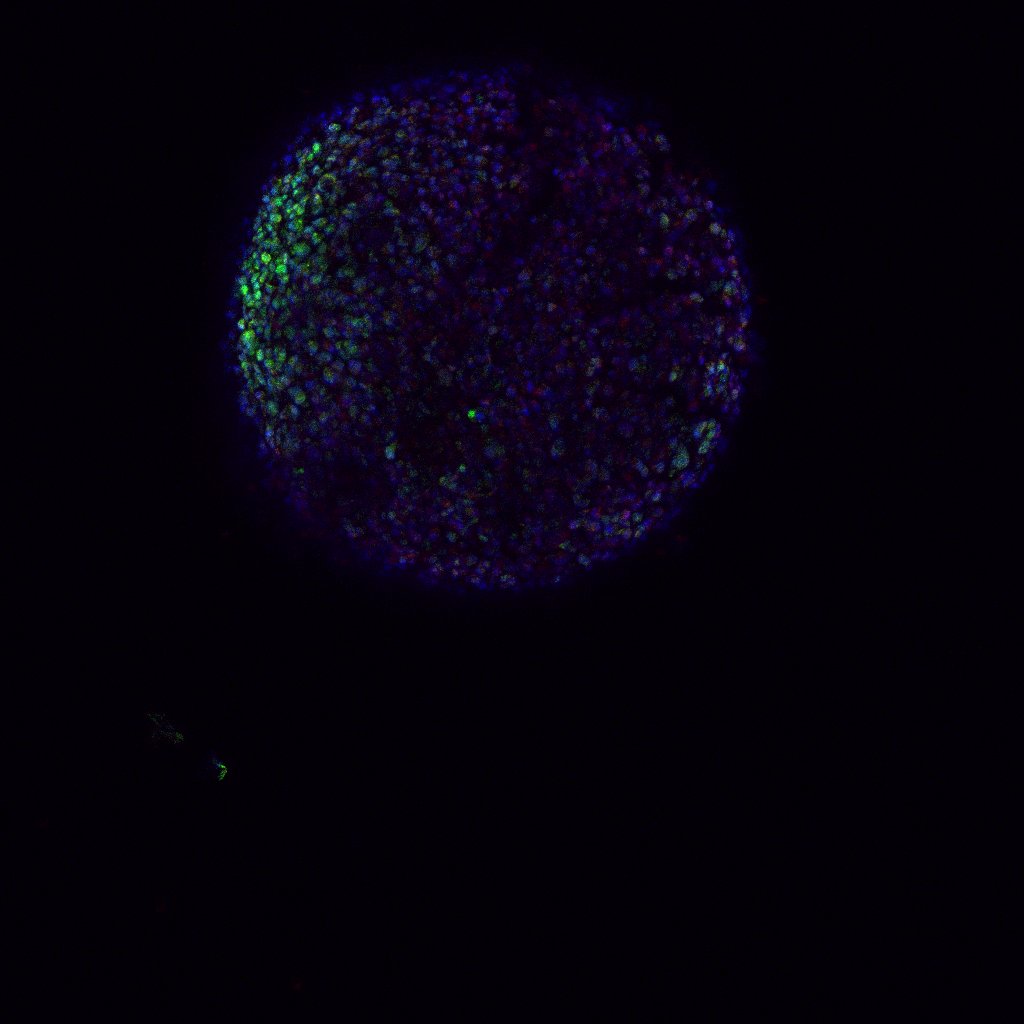

Supplement: Supplementary file 12 — Source data Fig. 6 [file 44321_2025_195_MOESM12_ESM.zip › Figure 6/6L/Figure 6L H2DCFDA MitoSOX DAPI IR.jpg]

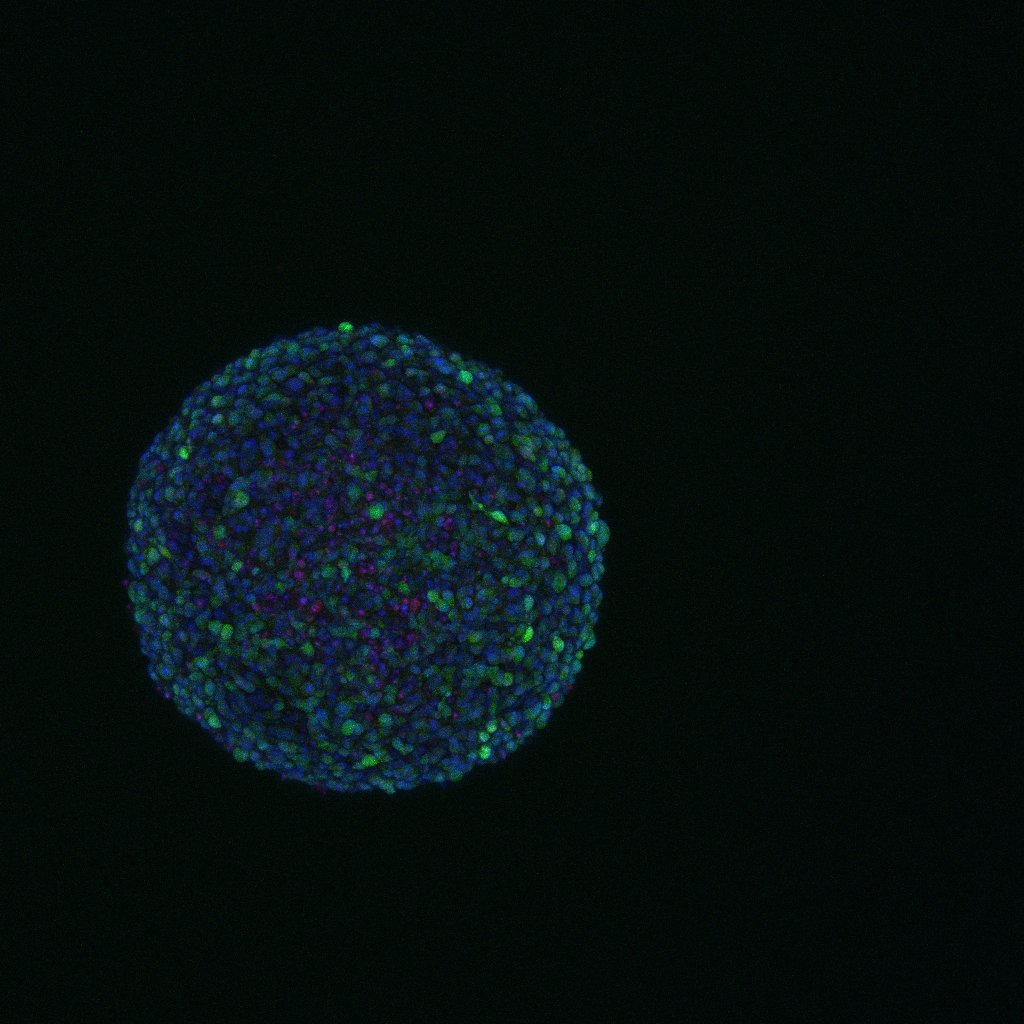

Supplement: Supplementary file 12 — Source data Fig. 6 [file 44321_2025_195_MOESM12_ESM.zip › Figure 6/6J/Figure 6J mubritinib + IR H2DCFDA MitosXOX DAPI.jpg]

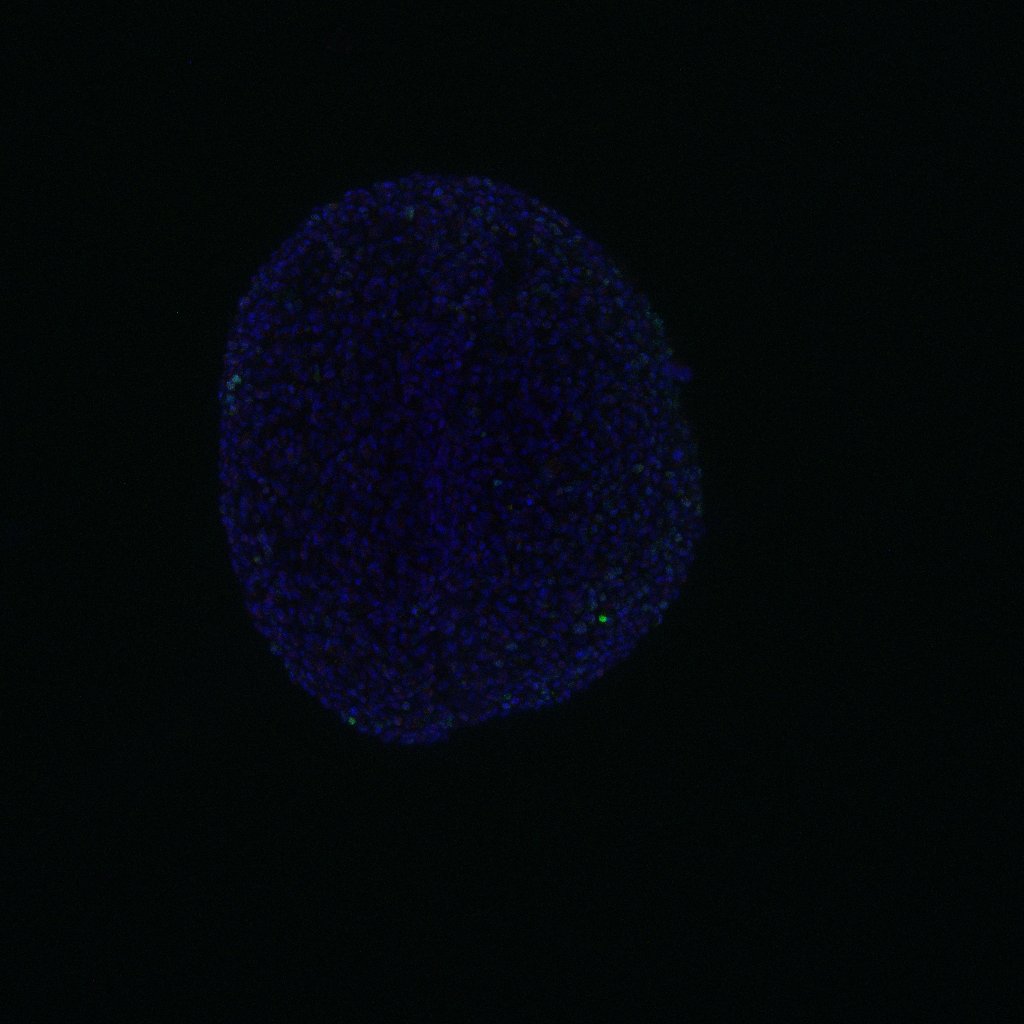

Supplement: Supplementary file 12 — Source data Fig. 6 [file 44321_2025_195_MOESM12_ESM.zip › Figure 6/6J/Figure 6J IR H2DCFDA MitosXOX DAPI.jpg]

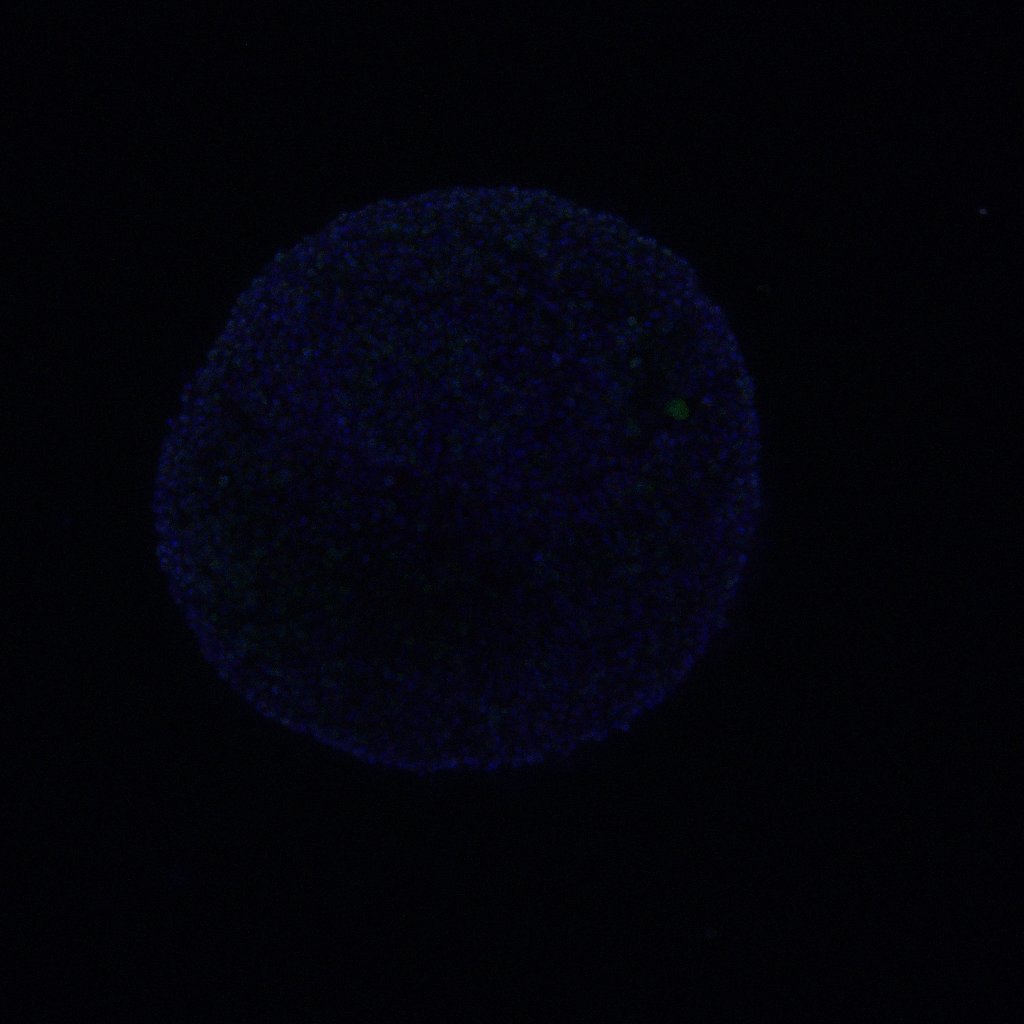

Supplement: Supplementary file 12 — Source data Fig. 6 [file 44321_2025_195_MOESM12_ESM.zip › Figure 6/6J/Figure 6J Control H2DCFDA MitosXOX DAPI.jpg]

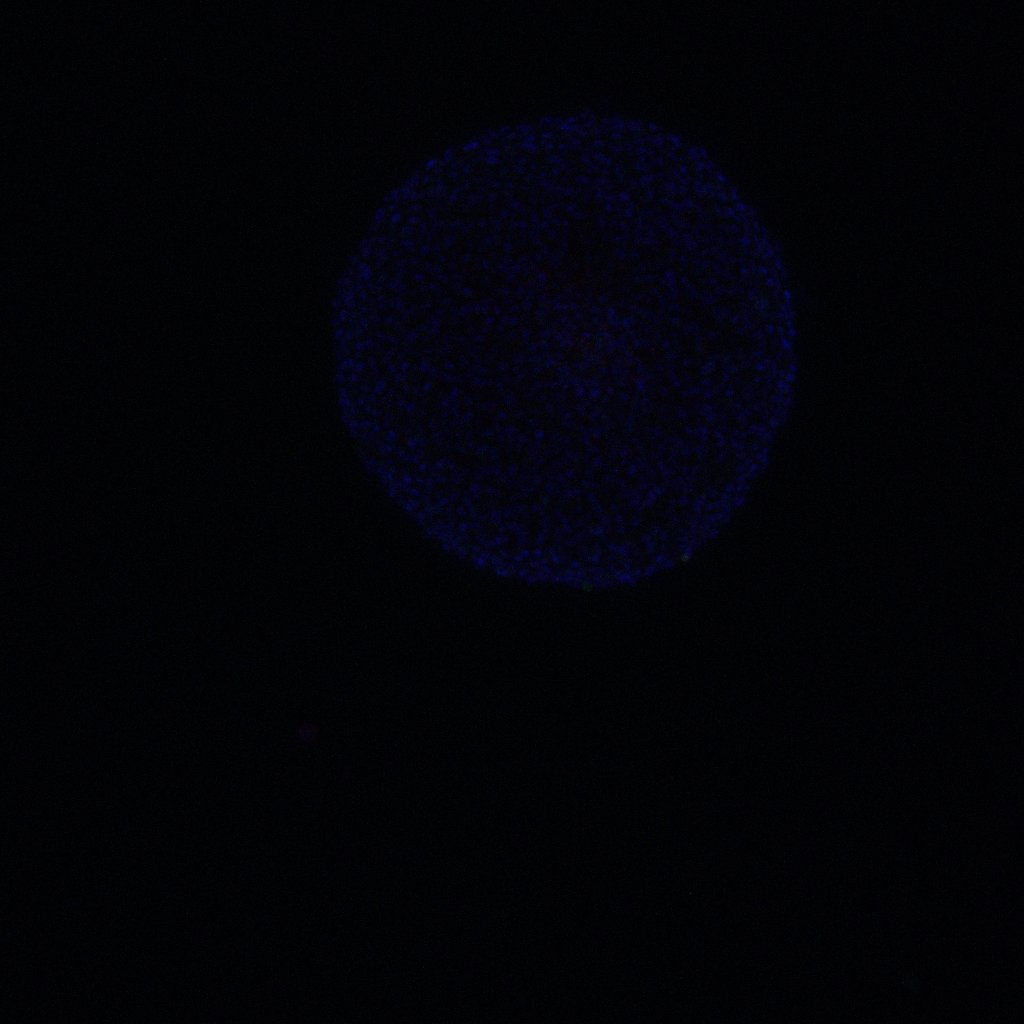

Supplement: Supplementary file 12 — Source data Fig. 6 [file 44321_2025_195_MOESM12_ESM.zip › Figure 6/6J/Figure 6J mubritinib H2DCFDA MitosXOX DAPI.jpg]

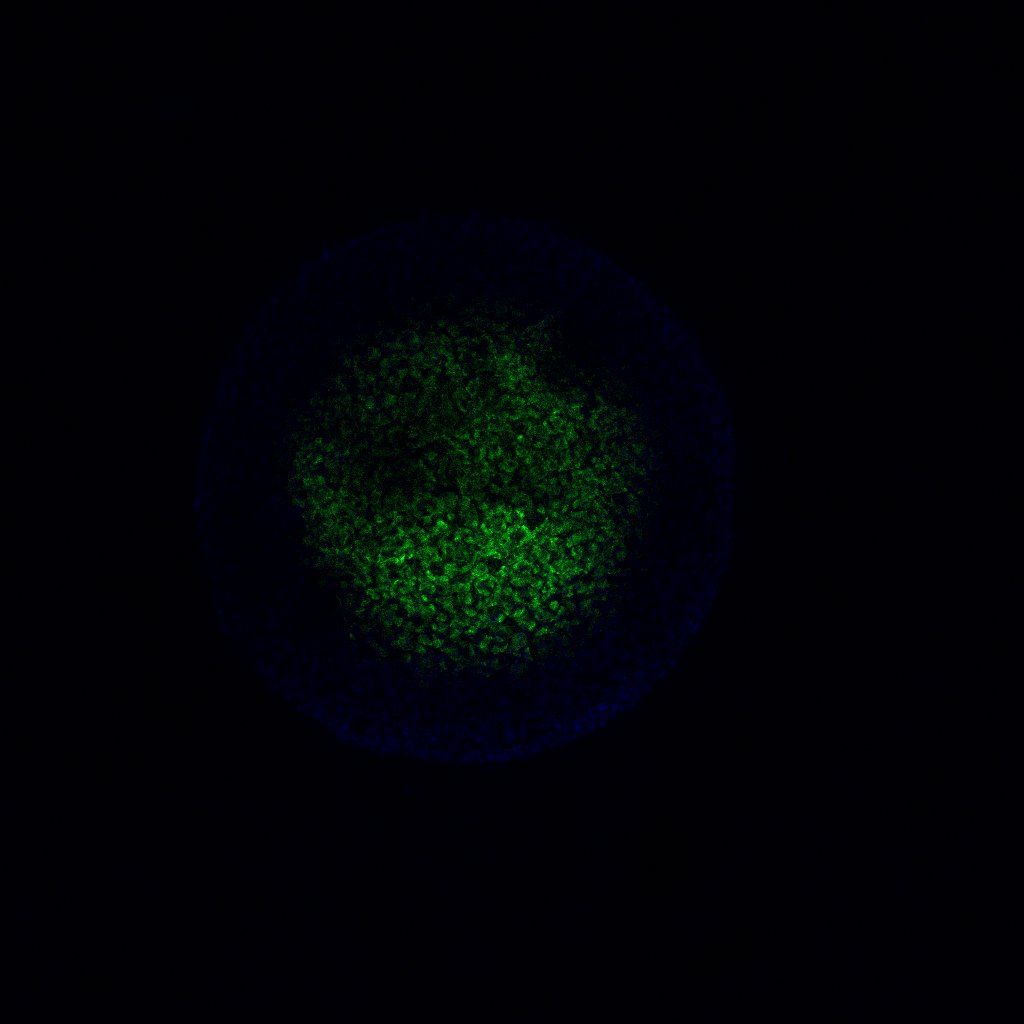

Supplement: Supplementary file 12 — Source data Fig. 6 [file 44321_2025_195_MOESM12_ESM.zip › Figure 6/6C/Figure 6C control normoxia.jpg]

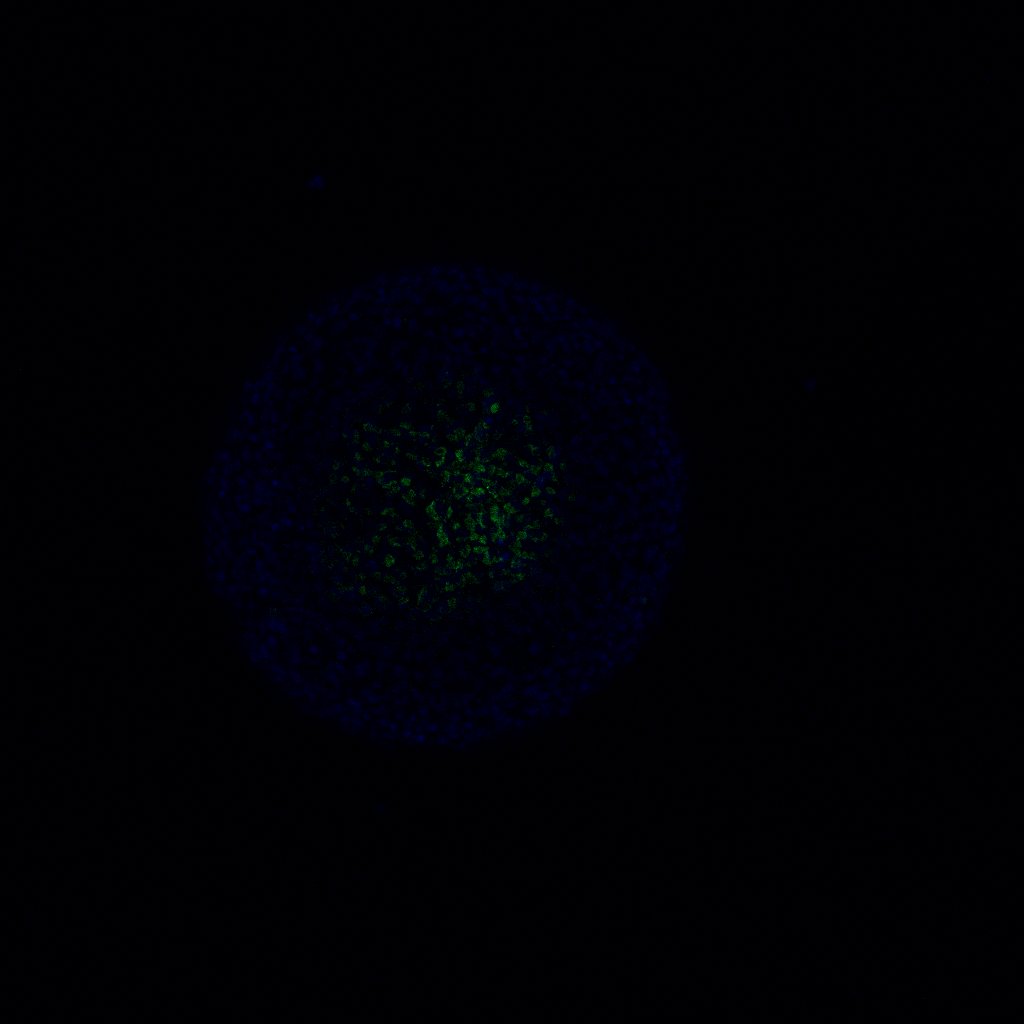

Supplement: Supplementary file 12 — Source data Fig. 6 [file 44321_2025_195_MOESM12_ESM.zip › Figure 6/6C/Figure 6C mubritinib normoxia.jpg]

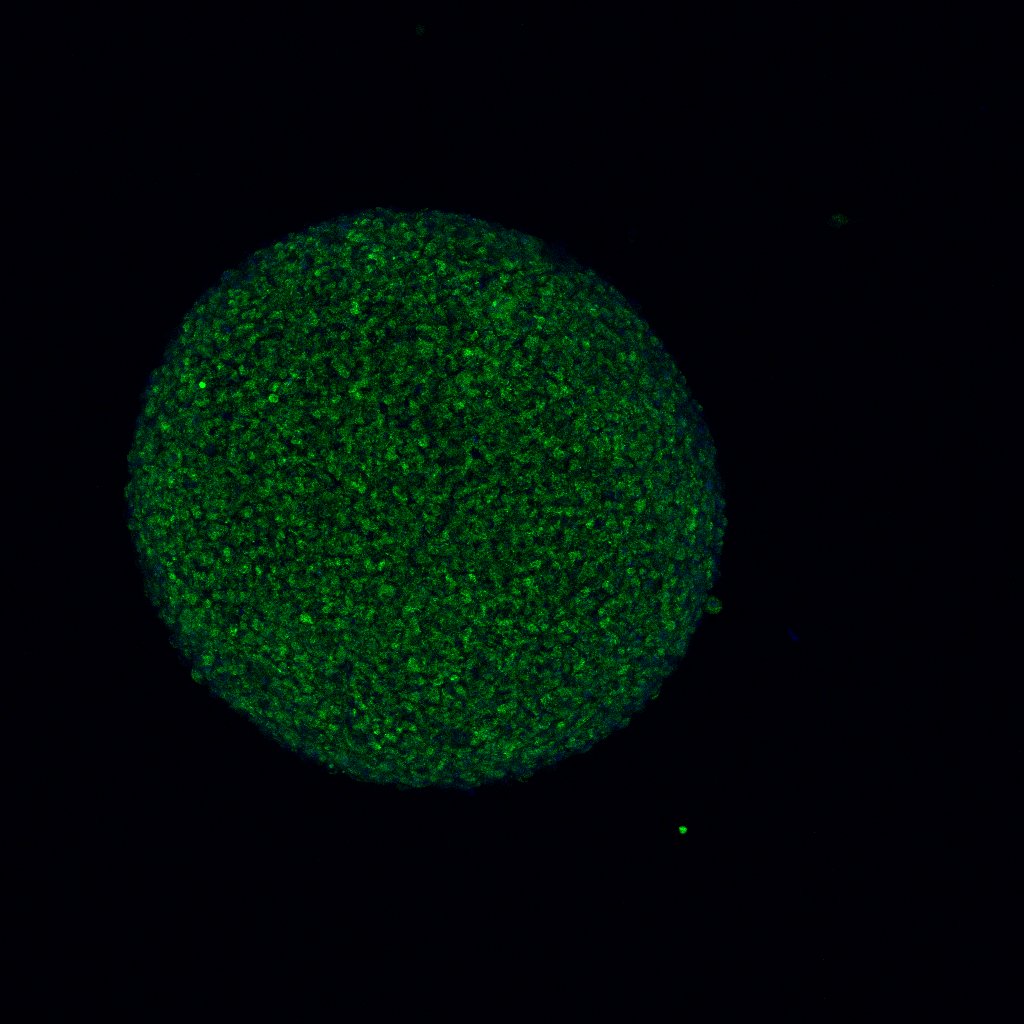

Supplement: Supplementary file 12 — Source data Fig. 6 [file 44321_2025_195_MOESM12_ESM.zip › Figure 6/6C/Figure 6C control hypoxia.jpg]

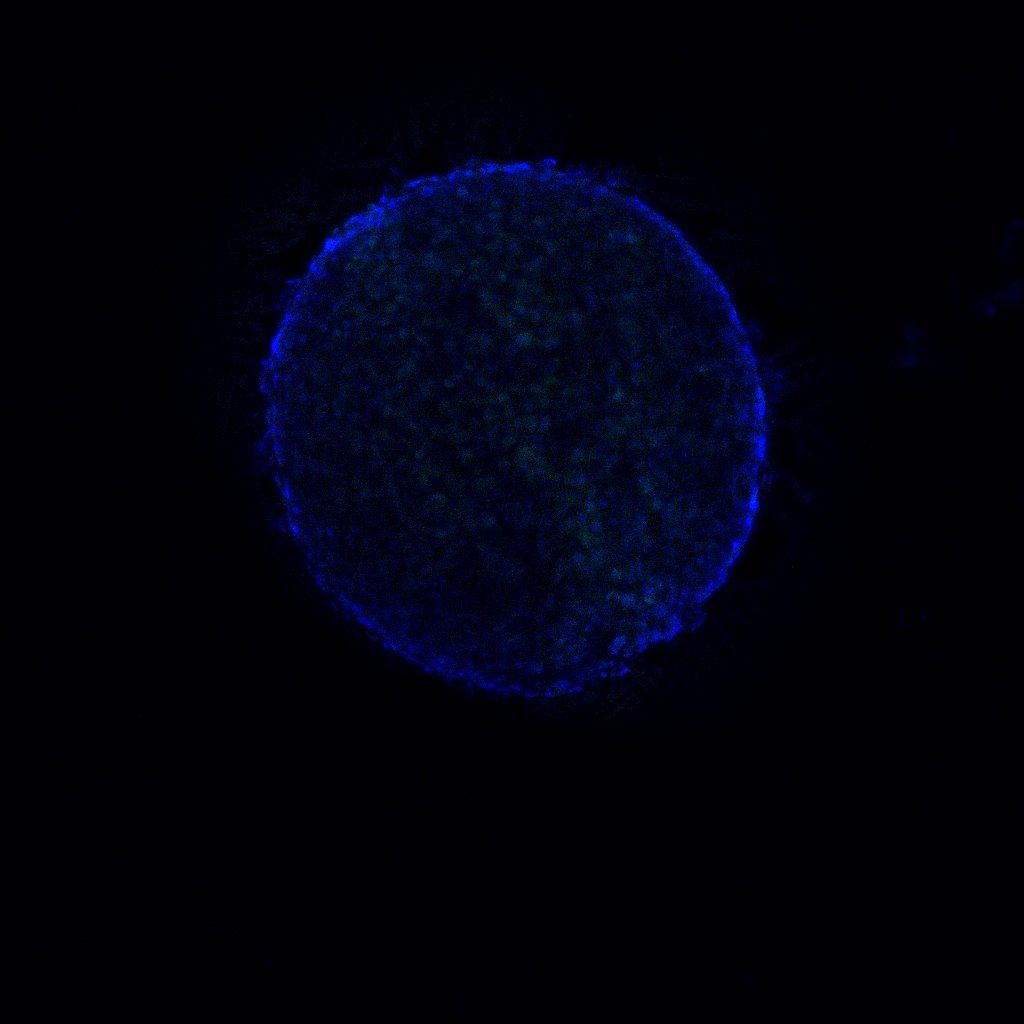

Supplement: Supplementary file 12 — Source data Fig. 6 [file 44321_2025_195_MOESM12_ESM.zip › Figure 6/6C/Figure 6C mubritinib hypoxia.jpg]

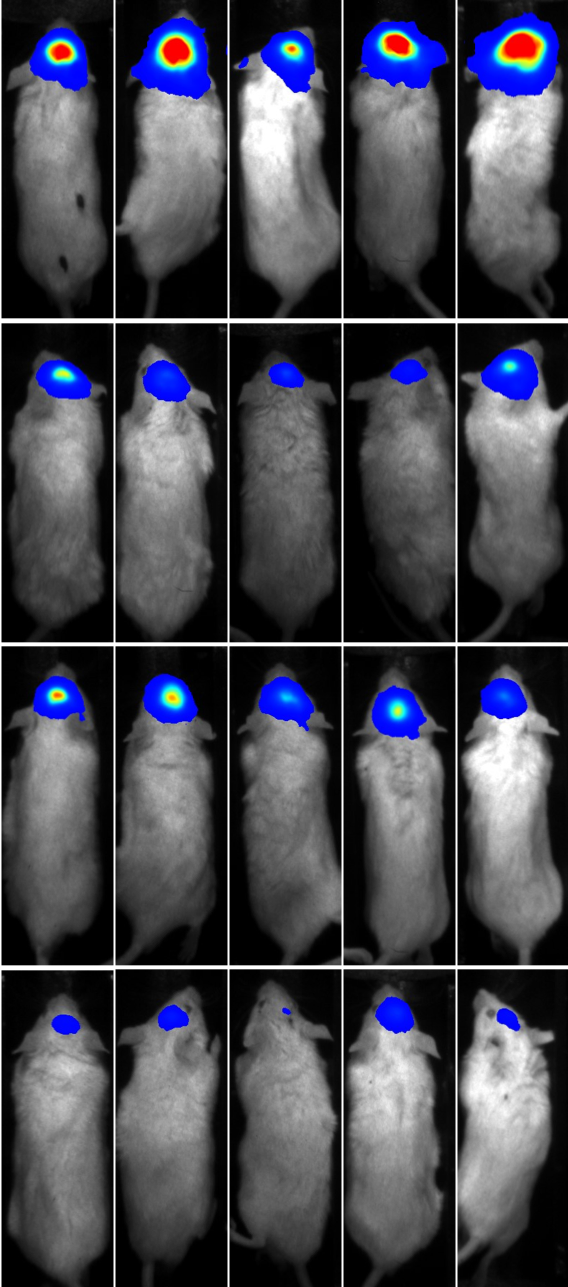

Supplement: Supplementary file 13 — Source data Fig. 7 [file 44321_2025_195_MOESM13_ESM.zip › Figure 7/7D/Figure 7D.png]

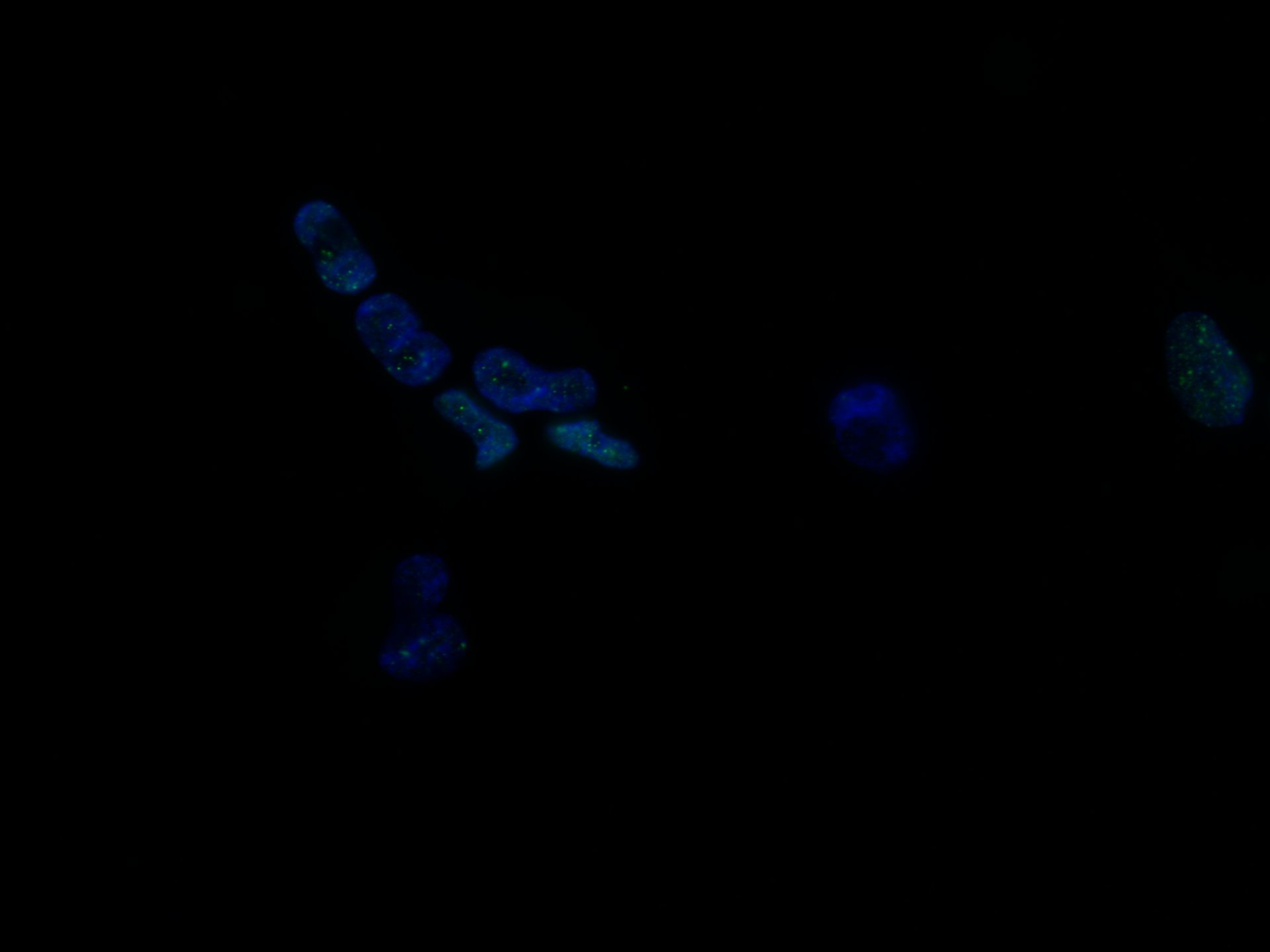

Supplement: Supplementary file 13 — Source data Fig. 7 [file 44321_2025_195_MOESM13_ESM.zip › Figure 7/7C/Figure 7C pH2AX DAPI control.jpg]

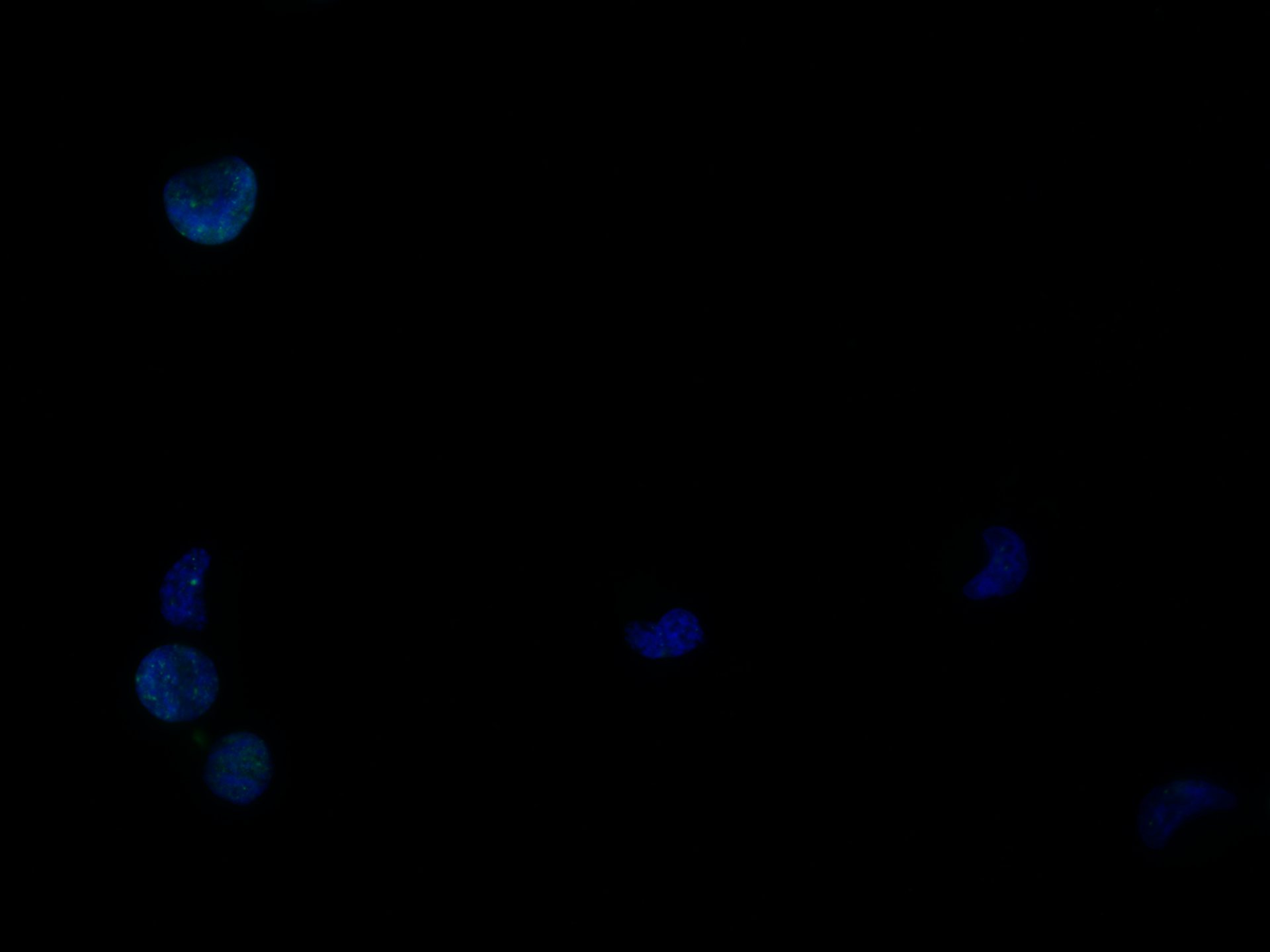

Supplement: Supplementary file 13 — Source data Fig. 7 [file 44321_2025_195_MOESM13_ESM.zip › Figure 7/7C/Figure 7C pH2AX DAPI Mubritinib.jpg]

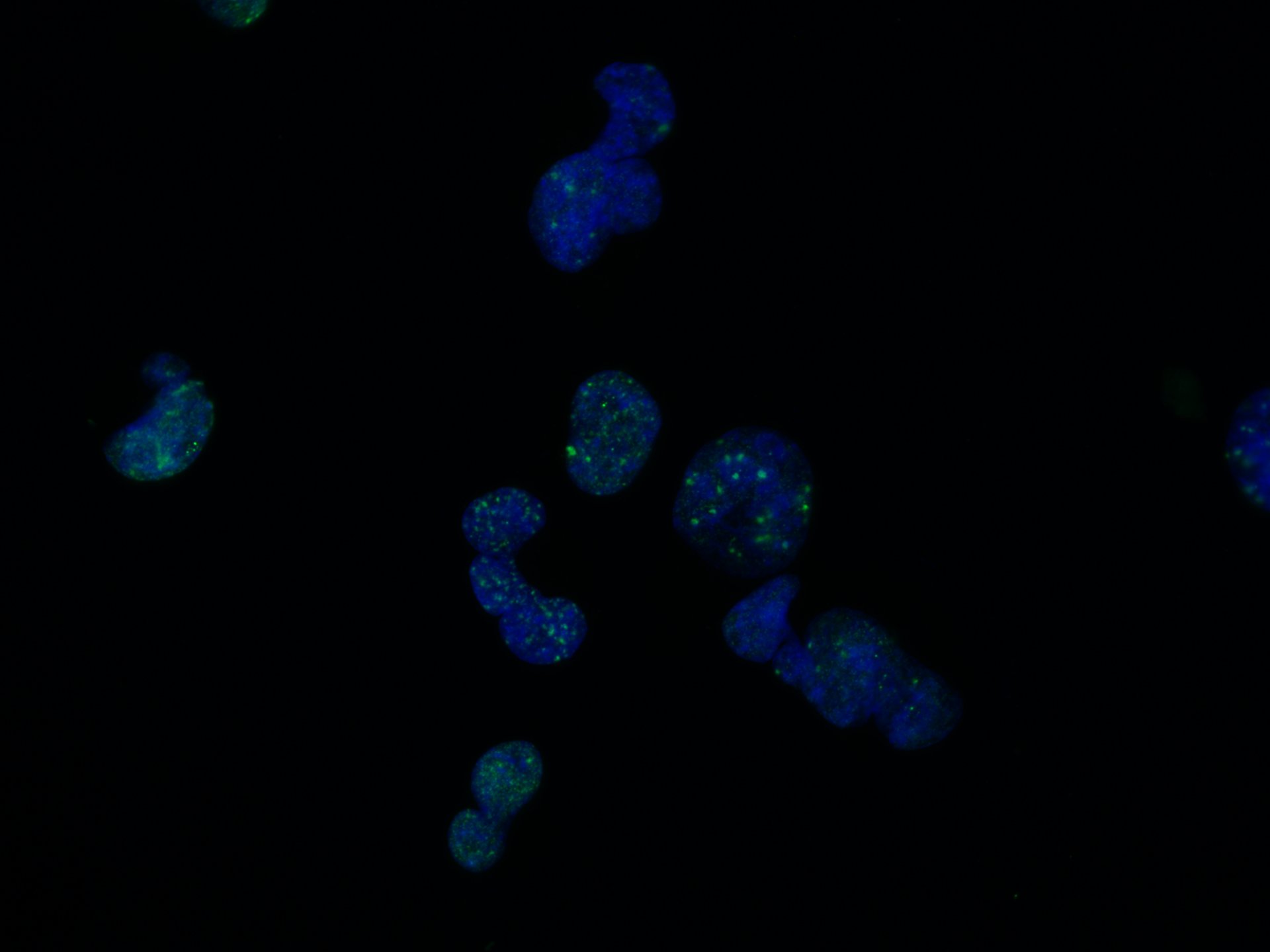

Supplement: Supplementary file 13 — Source data Fig. 7 [file 44321_2025_195_MOESM13_ESM.zip › Figure 7/7C/Figure 7C pH2AX DAPI TMZ.jpg]

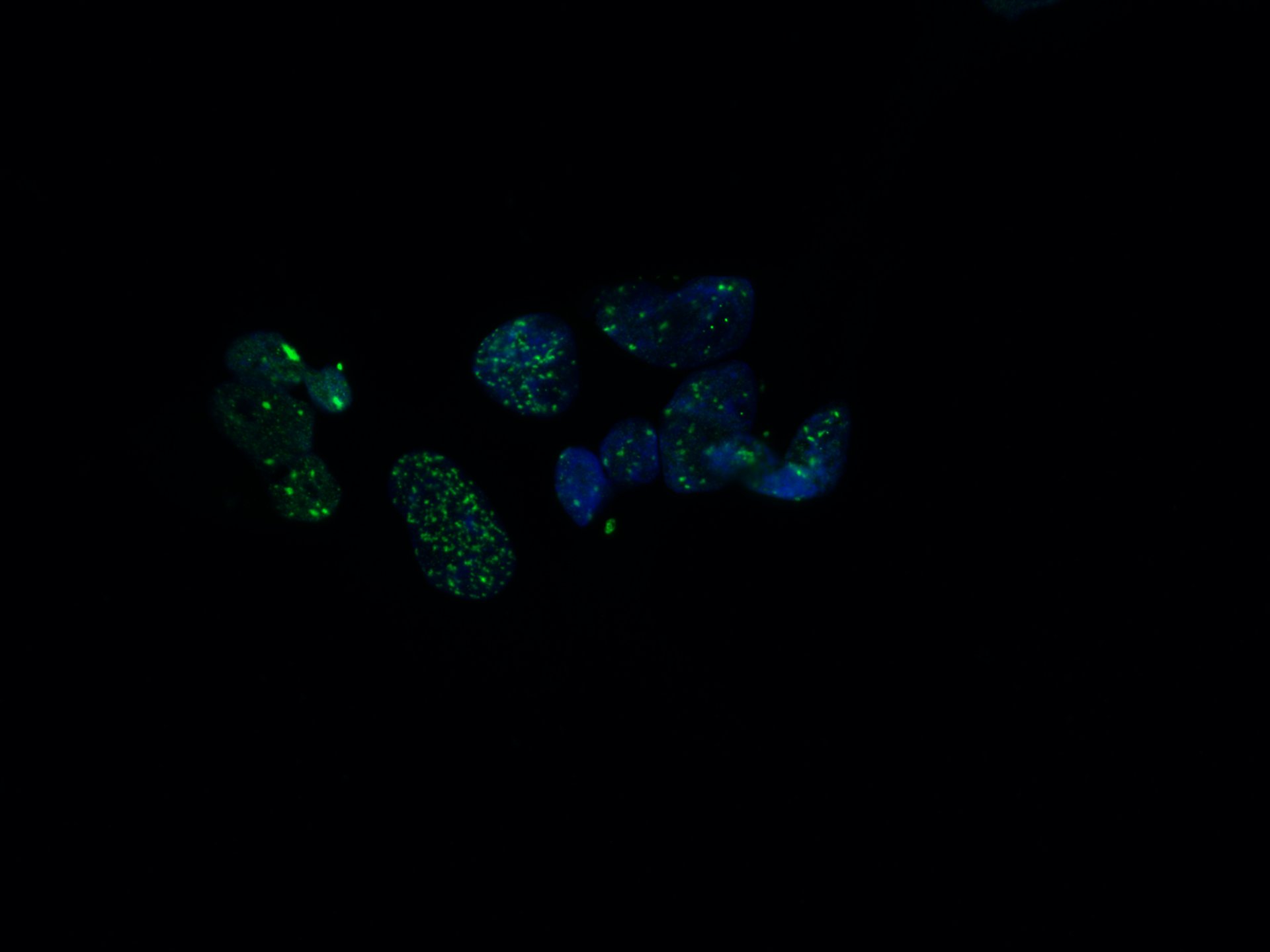

Supplement: Supplementary file 13 — Source data Fig. 7 [file 44321_2025_195_MOESM13_ESM.zip › Figure 7/7C/Figure 7C pH2AX DAPI Mubritinib + TMZ.jpg]

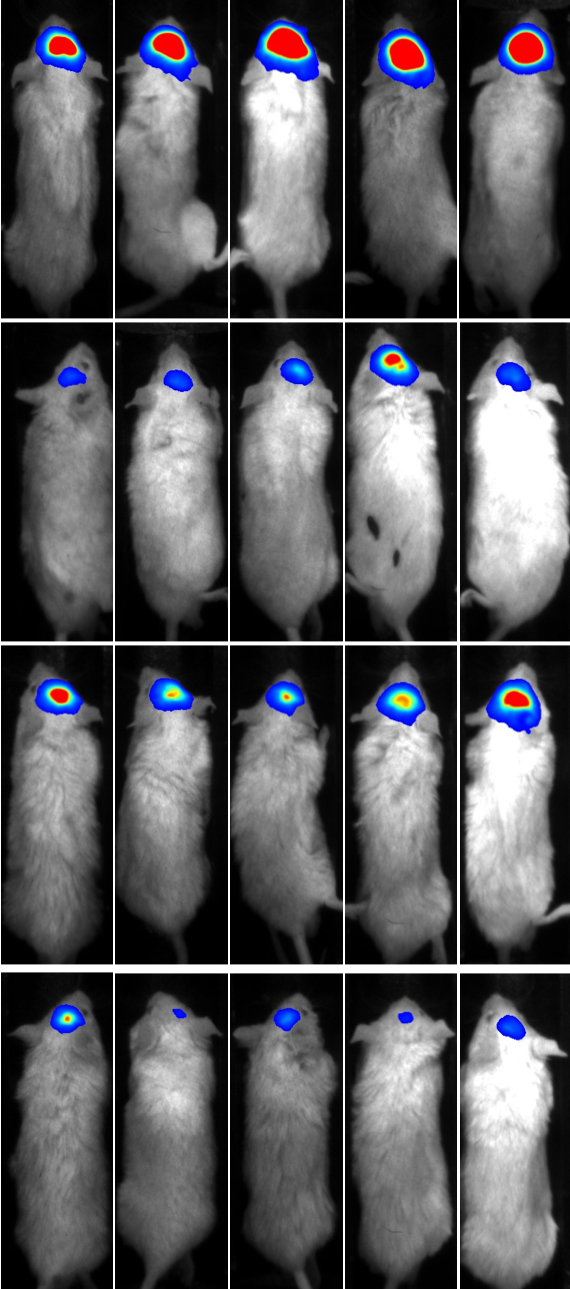

Supplement: Supplementary file 13 — Source data Fig. 7 [file 44321_2025_195_MOESM13_ESM.zip › Figure 7/7H/Figure 7H.png]

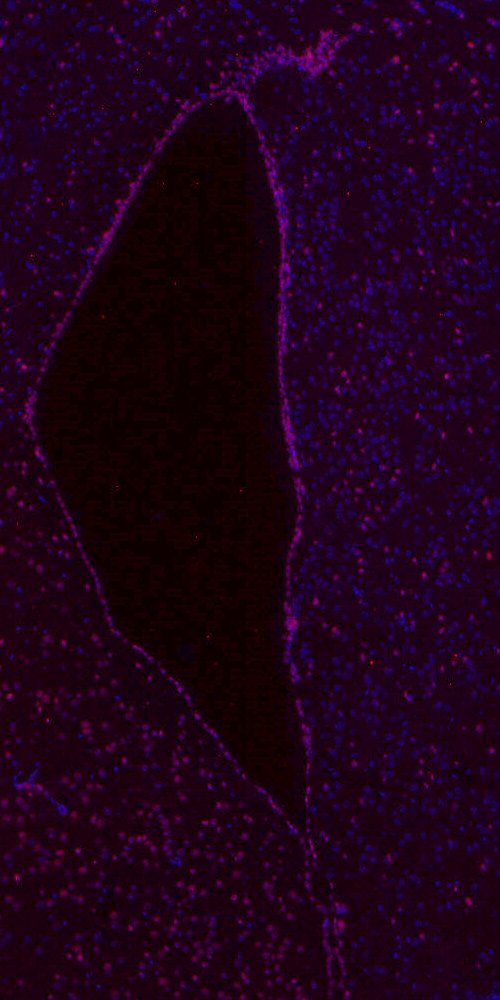

Supplement: Supplementary file 14 — Source data Fig. 8 [file 44321_2025_195_MOESM14_ESM.zip › Figure 8/8H/Figure 8H vehicle control.jpg]

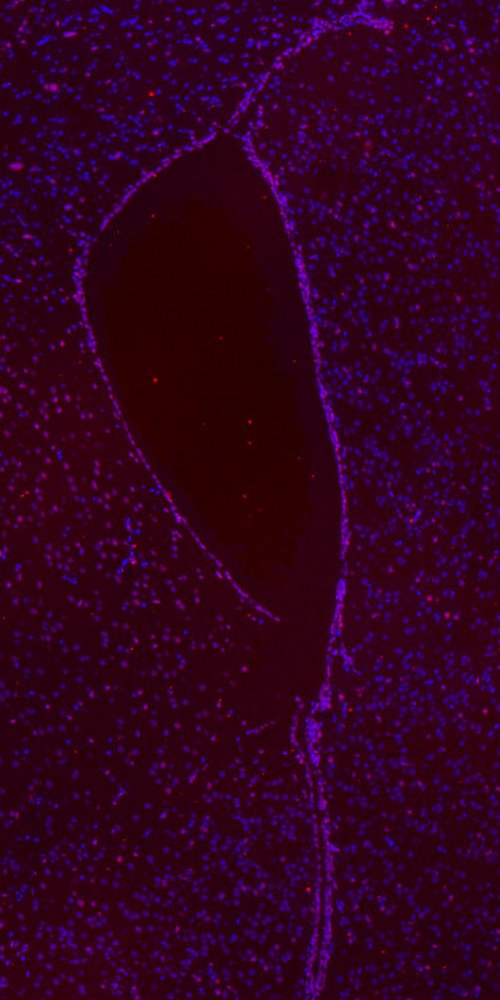

Supplement: Supplementary file 14 — Source data Fig. 8 [file 44321_2025_195_MOESM14_ESM.zip › Figure 8/8H/Figure 8H mubritinib.jpg]

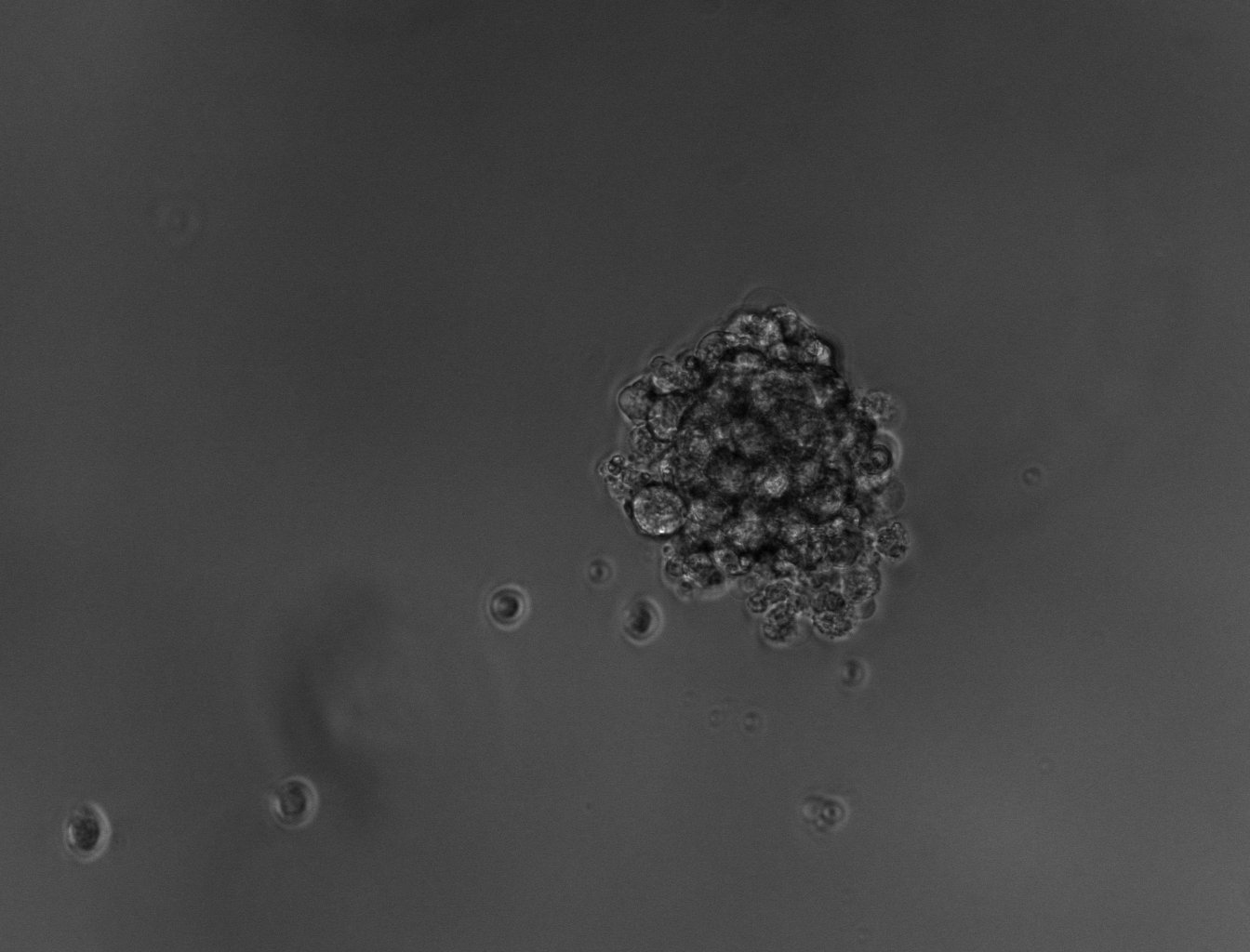

Supplement: Supplementary file 14 — Source data Fig. 8 [file 44321_2025_195_MOESM14_ESM.zip › Figure 8/8B/Figure 8B TMZ.jpg]

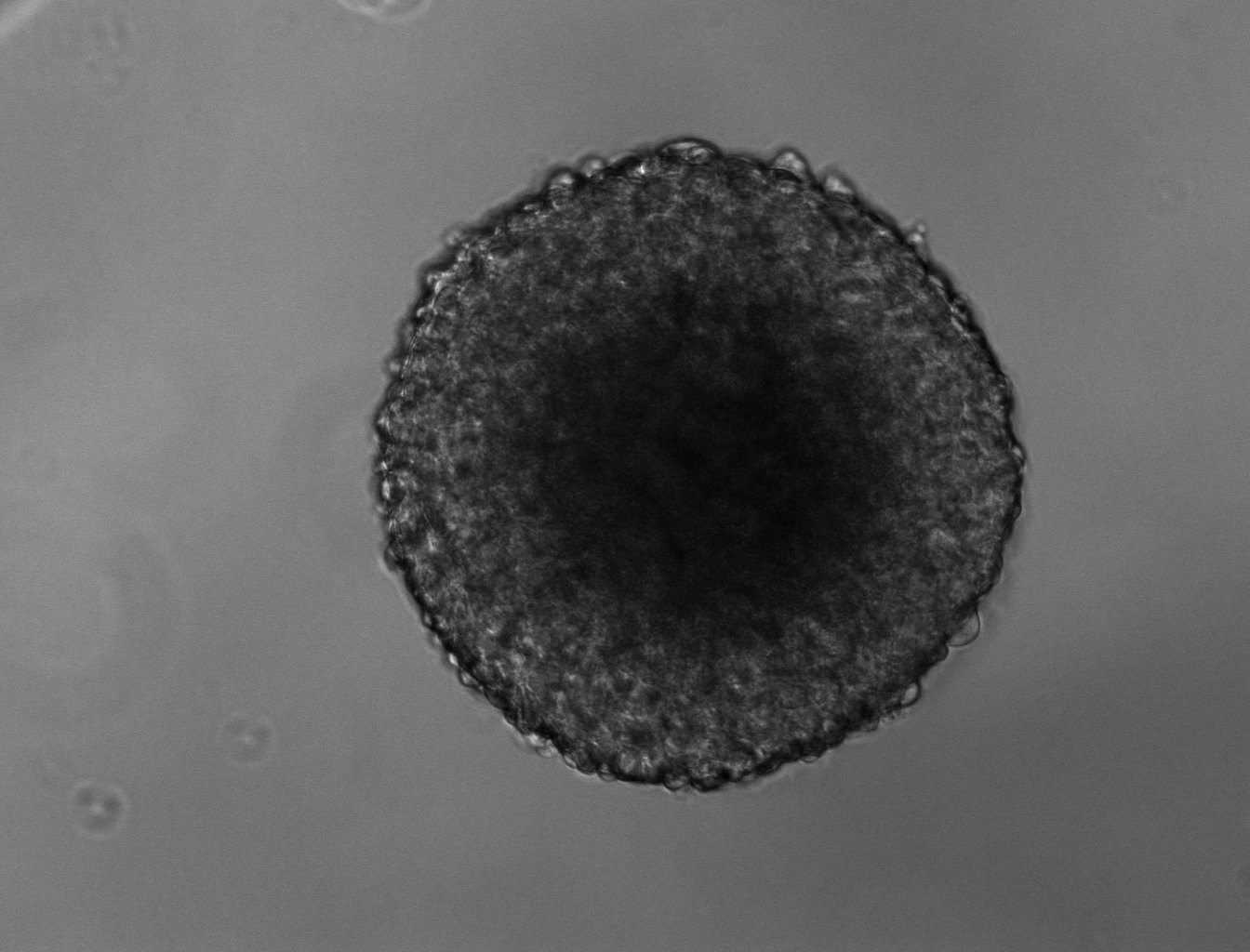

Supplement: Supplementary file 14 — Source data Fig. 8 [file 44321_2025_195_MOESM14_ESM.zip › Figure 8/8B/Figure 8B mubritinib 500.jpg]

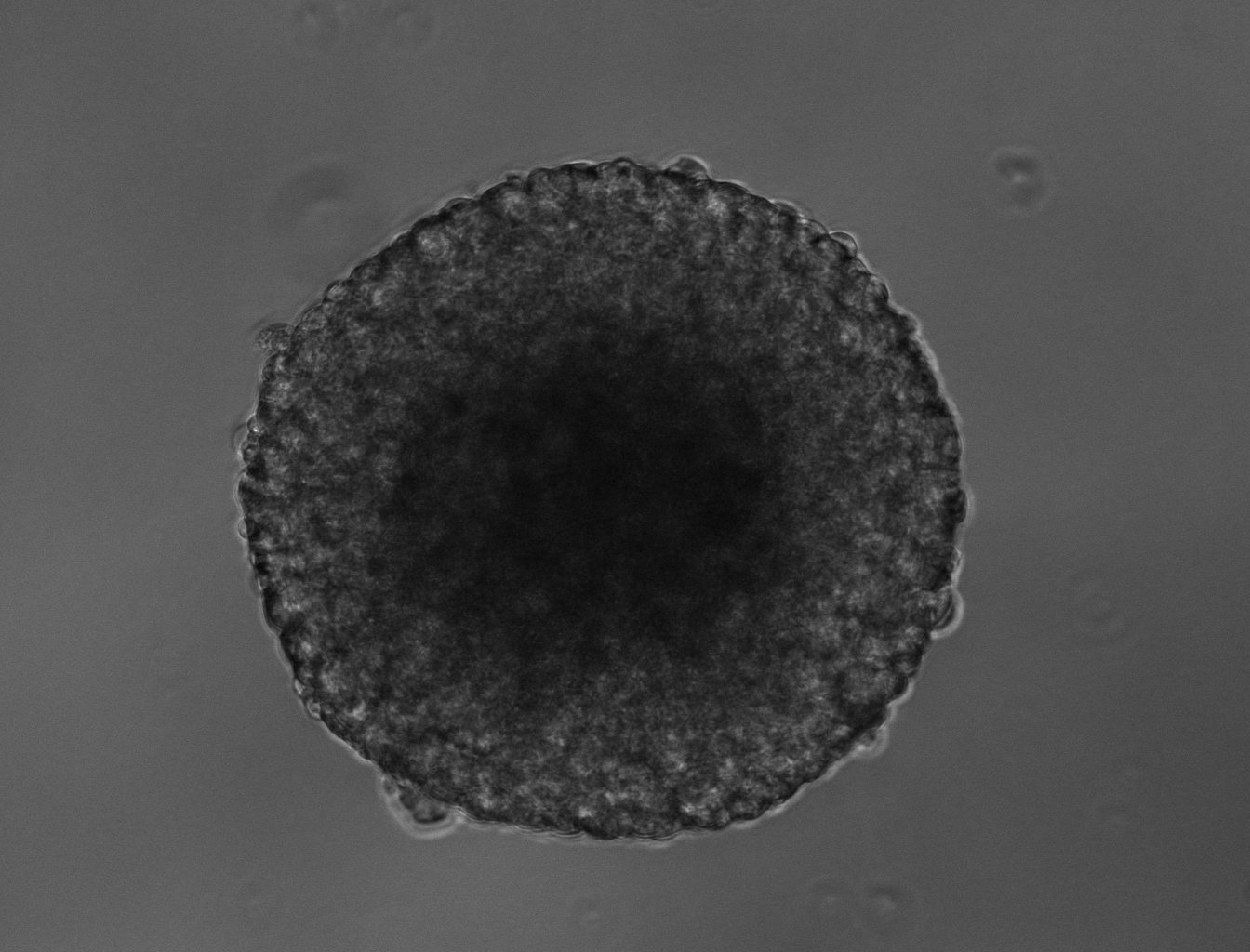

Supplement: Supplementary file 14 — Source data Fig. 8 [file 44321_2025_195_MOESM14_ESM.zip › Figure 8/8B/Figure 8B mubritinib 20.jpg]

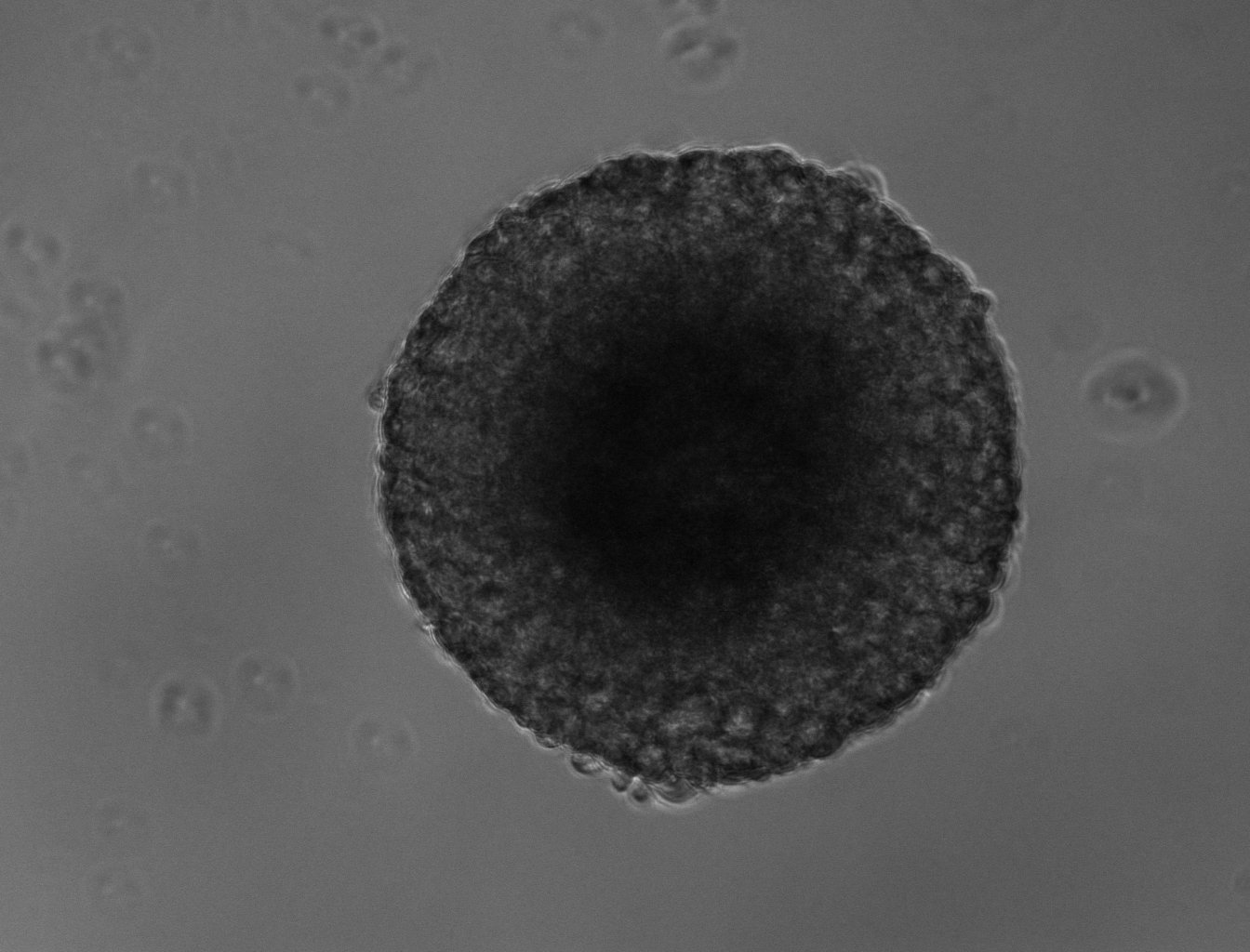

Supplement: Supplementary file 14 — Source data Fig. 8 [file 44321_2025_195_MOESM14_ESM.zip › Figure 8/8B/Figure 8B control.jpg]

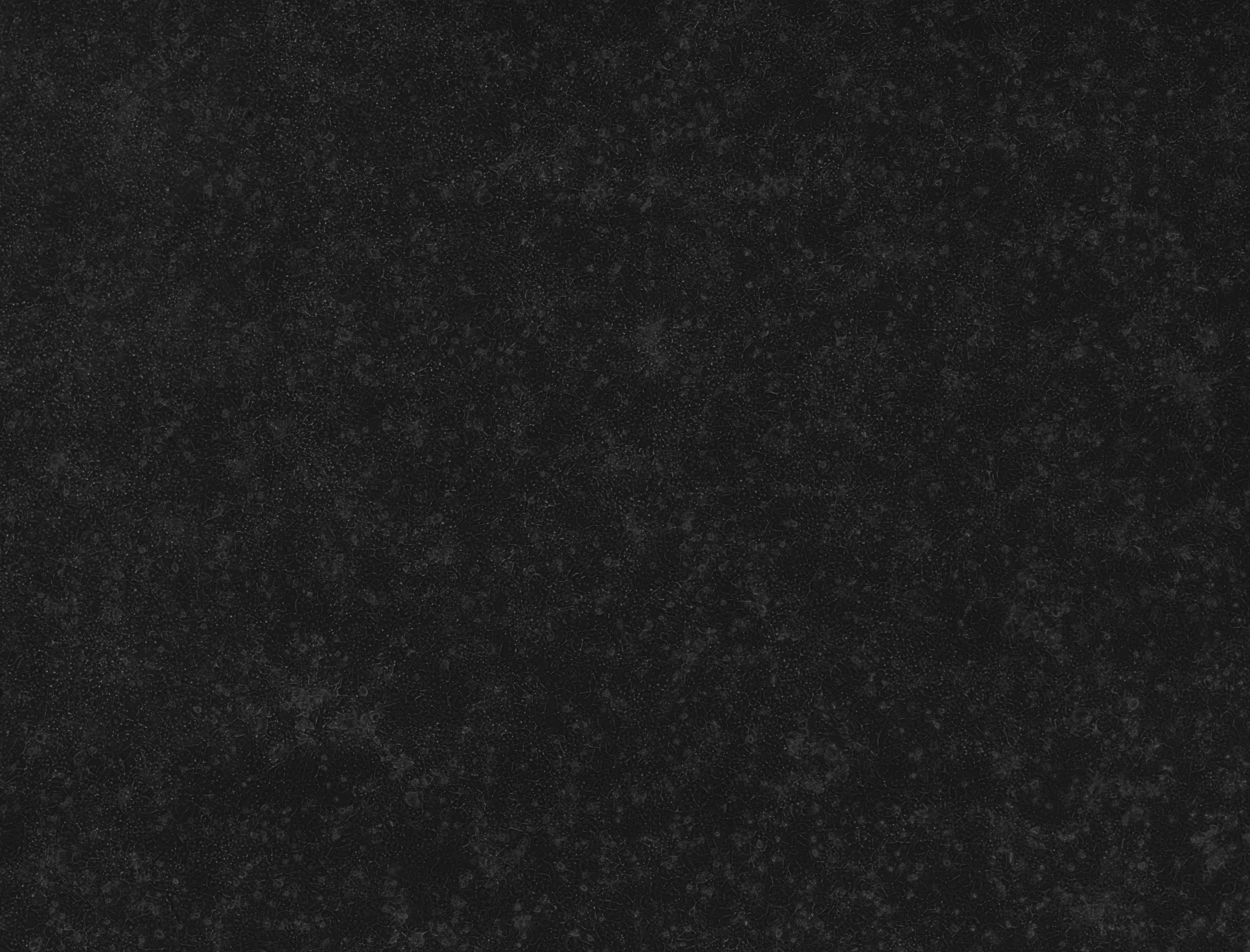

Supplement: Supplementary file 14 — Source data Fig. 8 [file 44321_2025_195_MOESM14_ESM.zip › Figure 8/8E/Figure 8E mubritinib 500 nM.tif]

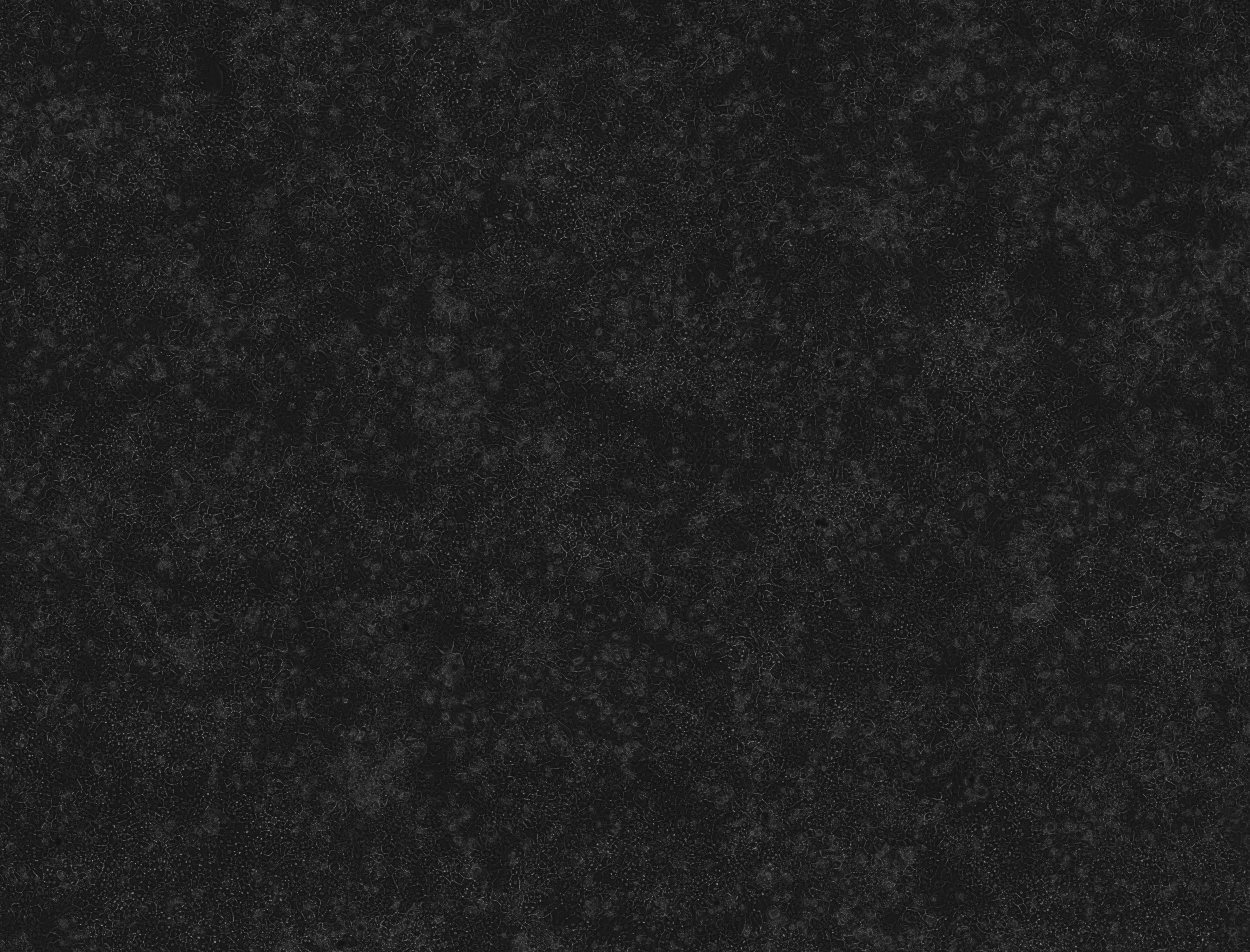

Supplement: Supplementary file 14 — Source data Fig. 8 [file 44321_2025_195_MOESM14_ESM.zip › Figure 8/8E/Figure 8E control.tif]

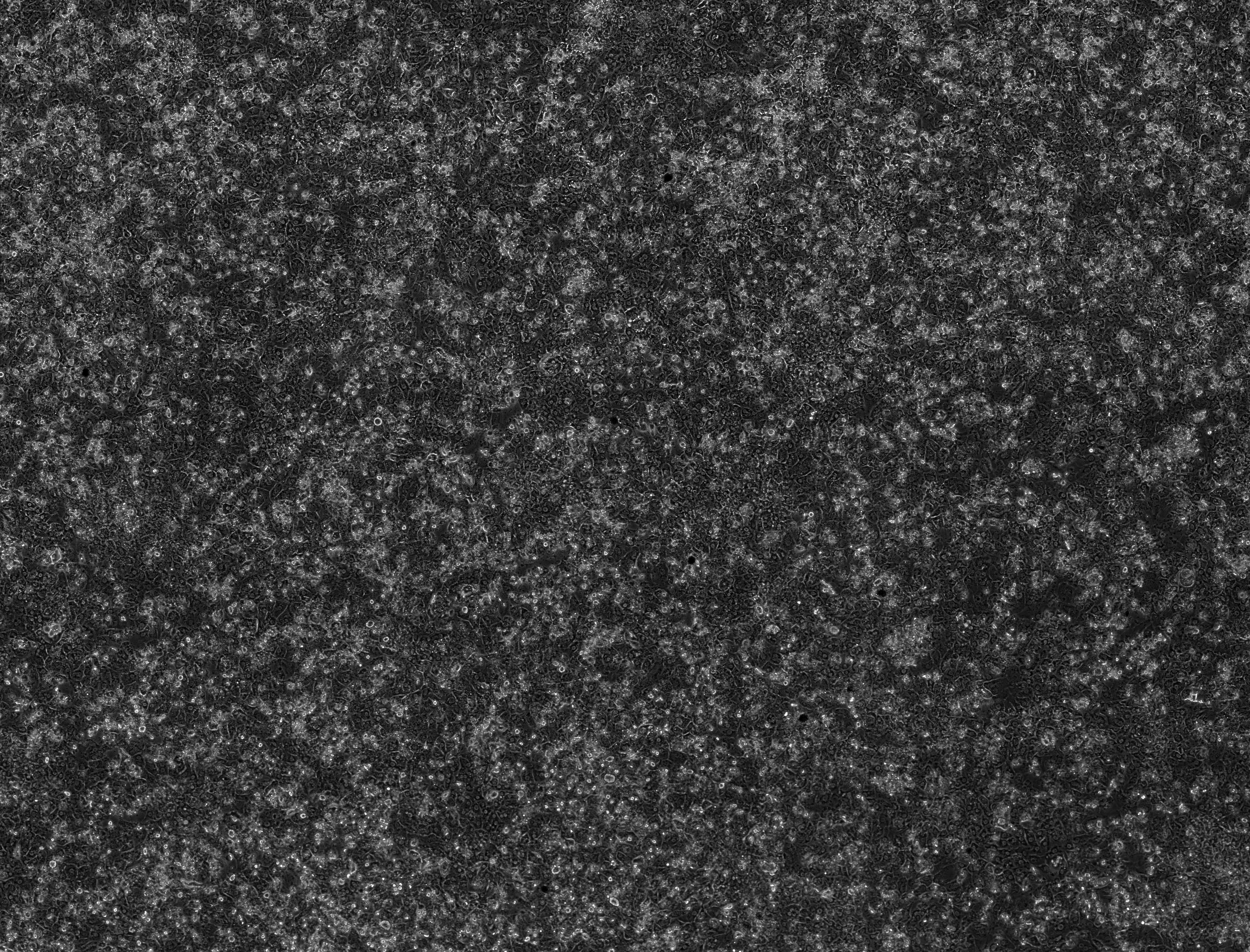

Supplement: Supplementary file 14 — Source data Fig. 8 [file 44321_2025_195_MOESM14_ESM.zip › Figure 8/8E/Figure 8E bosentan.tif]

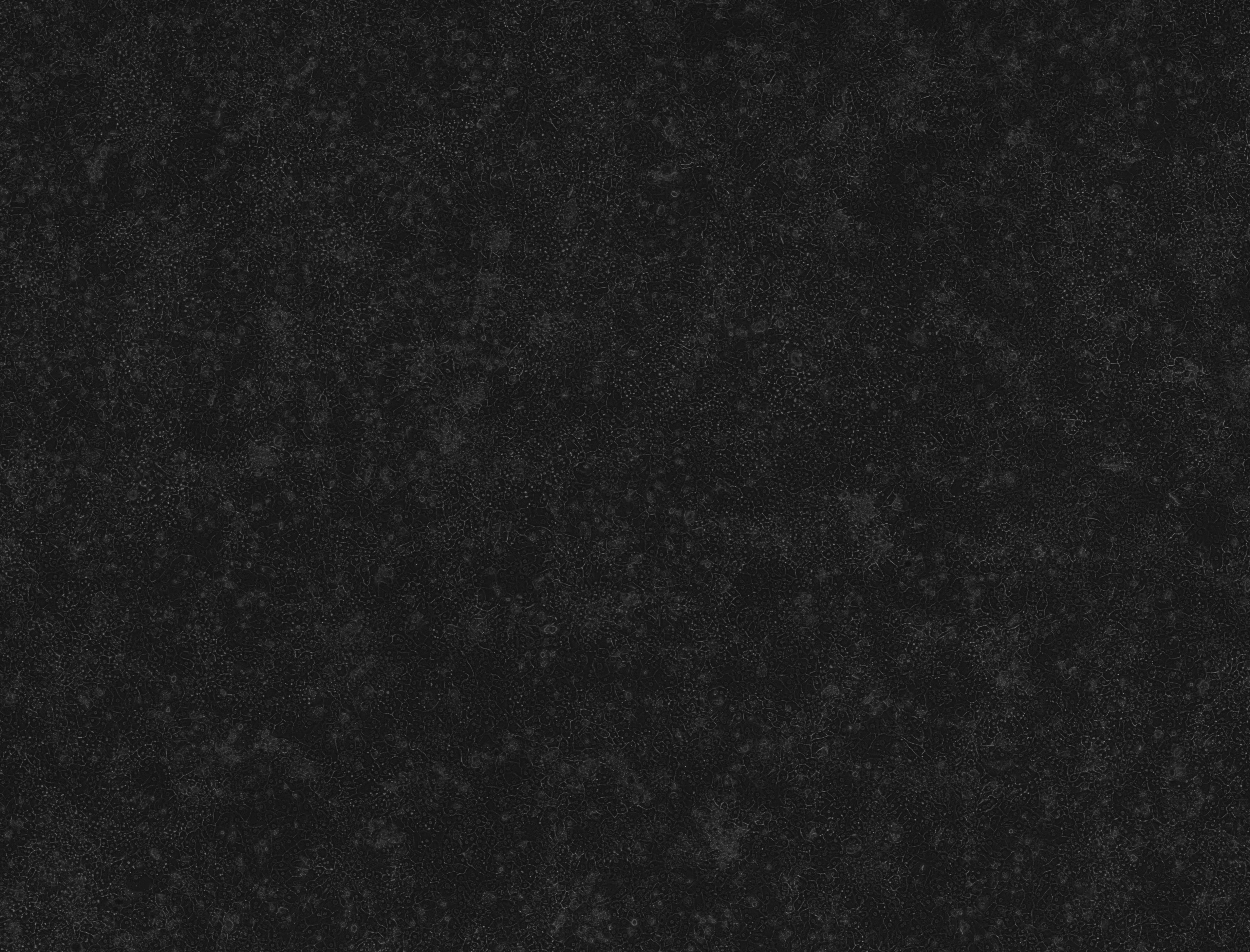

Supplement: Supplementary file 14 — Source data Fig. 8 [file 44321_2025_195_MOESM14_ESM.zip › Figure 8/8E/Figure 8E mubritinib 20 nM.tif]
