# Supplementary material for: Exploiting metabolic vulnerability in glioblastoma using a brain-penetrant drug with a safe profile
Source: EMBO Mol Med. 2025 Feb 3;17(3):469–503. doi: 10.1038/s44321-025-00195-6 (PMC11903783; doi:10.1038/s44321-025-00195-6)
Supplement: Supplementary file 15 — Expanded View Figures [file 44321_2025_195_MOESM15_ESM.pdf]

Expanded View Figures

Figure EV1. Mubritinib decreases the proliferation of patient- and murine-derived BTSCs without inducing cell death.

(**A**) Basal mitochondrial respiration, maximal mitochondrial respiration and spare respiratory capacity (SRC) were measured in BTSC73 following mubritinib treatment using the high-resolution respirometer Oroboros. Data are presented as the means ± SEM, $n = 3$ independent biological experiments. One-way ANOVA followed by Dunnett's test vs control. $^{**}p_{\text{basal (20)}} = 0.0012$; $^{***}p_{\text{basal (100)}} = 2.9\text{e}{-5}$; $^{***}p_{\text{basal (500)}} = 1.1\text{e}{-5}$, $^{*}p_{\text{maximal (20)}} = 0.0228$, $^{***}p_{\text{maximal (100)}} = 0.0009$, $^{***}p_{\text{maximal (500)}} = 0.0006$, $^{*}p_{\text{SRC (100)}} = 0.0488$, $^{*}p_{\text{SRC (500)}} = 0.0377$. (**B**) The murine glioblastoma cells, mGB2, were exposed to increasing concentrations of mubritinib (0 to 500 nM), followed by live cell counting. Data are presented as the means ± SEM, $n = 3$ independent biological experiments. One-way ANOVA followed by Dunnett's test vs control. $^{***}p_{\text{mubritinib 100}} = 0.0009$, $^{***}p_{\text{mubritinib 500}} = 4.4\text{e}{-5}$. (**C**) The percentages of dead cells (PI positive) were measured by PI staining followed by flow cytometry in BTSCs treated with increasing concentrations of mubritinib for 7 days. Data are presented as the means ± SEM, $n = 3$ independent biological experiments. One-way ANOVA followed by Dunnett's test vs control. *p*-values are higher than 0.05 for all conditions. (**D**) The percentages of dead cells (PI positive) and early apoptotic cells (Annexin V positive and PI negative) were measured by Annexin V/PI double staining followed by flow cytometry in BTSCs treated with increasing concentrations of mubritinib for 7 days. (**E–G**) Pearson correlation analysis was performed between basal OCR and the percentage of live cells in BTSC following 7 days treatment with mubritinib at 20 nM (**E**) 100 nM (**F**) and 500 nM (**G**). (**H**) Pearson correlation analysis was performed between maximal OCR and sensitivity score to mubritinib following 7 days of treatment. (**I–K**) mRNA levels of *NDI1* gene were assessed by RT-qPCR in BTCS53 (**I**), BTSC73 (**J**) and BTSC147 (**K**) transduced with control or NDI1 vector. Data are presented as the means ± SEM, $n = 3$ independent biological experiments. (**L–N**) Basal OCR was measured by Resipher system in BTSC53 (**L**), BTSC73 (**M**) and BTSC147 (**N**) expressing the control (CTL) vector or NDI1 following treatment with vehicle control or 500 nM mubritinib. Data are presented as the means ± SEM, $n = 3$ independent biological experiments. Two-way ANOVA followed by Tukey's test. BTSC53 (**L**): $^{***}p_{\text{CTL vs mubritinib}} = 1.8\text{e}{-12}$, $p_{\text{NDI1 vs NDI1 + mubritinib}} = 0.1702$. BTSC73 (**M**): $^{***}p_{\text{CTL vs mubritinib}} = 4.9\text{e}{-13}$, $p_{\text{NDI1 vs NDI1 + mubritinib}} = 0.6586$. BTSC147 (**N**): $^{***}p_{\text{CTL vs mubritinib}} = 1\text{e}{-10}$, $p_{\text{NDI1 vs NDI1 + mubritinib}} = 0.0828$. (**O**) Proliferation curve was generated using the CFSE assay in BTSC73 expressing the CTL vector or NDI1 following treatment with vehicle control or 500 nM mubritinib. Data are presented as the means ± SEM, $n = 3$ independent biological experiments. Two-way ANOVA followed by Tukey's test. $^{*}p_{\text{3d (CTL vs mubritinib)}} = 0.0267$, $^{*}p_{\text{4d (CTL vs mubritinib)}} = 0.0265$, $^{*}p_{\text{5d (CTL vs mubritinib)}} = 0.0312$, $^{**}p_{\text{6d (CTL vs mubritinib)}} = 0.0011$, $p_{\text{NDI1 vs NDI1 + mubritinib}} > 0.05$ at all the time points. (**P**, **Q**) mRNA levels of *NDUFS7* gene were assessed by RT-qPCR in BTCS73 (**P**), BTSC53 (**Q**) electroporated with either siCTL or si*NDUFS7*. Data are presented as the means ± SEM, $n = 3$ independent biological experiments. Unpaired two-tailed t test. $^{***}p_{\text{BTSC73}} = 5\text{e}{-8}$, $^{***}p_{\text{BTSC53}} = 4.8\text{e}{-6}$. (**R**, **S**) BTSC73 (**R**) and BTSC53 (**S**) were electroporated with either siCTL or si*NDUFS7* and treated with 500 nM of mubritinib for 7 days, followed by live cell counting. Data are presented as the means ± SEM, $n = 3$ independent biological experiments. One-way ANOVA followed by Tukey's test. BTSC73 (**R**): $^{***}p_{\text{siCTL vs siCTL + mubritinib}} = 1.6\text{e}{-6}$, $^{***}p_{\text{siCTL vs si}NDUFS7} = 5.3\text{e}{-6}$, $p_{\text{si}NDUFS7\text{ vs si}NDUFS7\text{ + mubritinib}} = 0.0690$. BTSC53 (**S**): $^{***}p_{\text{siCTL vs siCTL + mubritinib}} = 3\text{e}{-5}$, $^{***}p_{\text{siCTL vs si}NDUFS7} = 6.8\text{e}{-6}$, $p_{\text{si}NDUFS7\text{ vs si}NDUFS7\text{ + mubritinib}} = 0.5667$. (**T**) BTSCs and differentiated progeny counterparts (Diff) were subjected to immunoblotting using the antibodies indicated on the blots. Vinculin is used as loading control. (**U–X**) The number of live stem and differentiated cells from BTSC12 (**U**), BTSC53 (**V**), BTSC73 (**W**) and BTSC147 (**X**) was measured following 500 nM mubritinib treatment. Data are presented as the means ± SEM, $n = 3$ independent biological experiments. One-way ANOVA followed by Tukey's test. BTSC12 (**U**): $^{***}p_{\text{stem control vs mubritinib}} = 0.0002$, $^{*}p_{\text{diff control vs mubritinib}} = 0.0426$, $^{**}p_{\text{stem mubritinib vs diff mubritinib}} = 0.0078$. BTSC53 (**V**): $^{***}p_{\text{stem control vs mubritinib}} = 9.4\text{e}{-5}$, $^{**}p_{\text{stem mubritinib vs diff mubritinib}} = 0.0010$. BTSC73 (**W**): $^{***}p_{\text{stem control vs mubritinib}} = 0.0001$, $^{*}p_{\text{diff control vs mubritinib}} = 0.0322$, $^{**}p_{\text{stem mubritinib vs diff mubritinib}} = 0.0049$. BTSC147 (**X**): $^{***}p_{\text{stem control vs mubritinib}} = 5.1\text{e}{-5}$, $^{***}p_{\text{stem mubritinib vs diff mubritinib}} = 0.0007$.

© The Author(s)

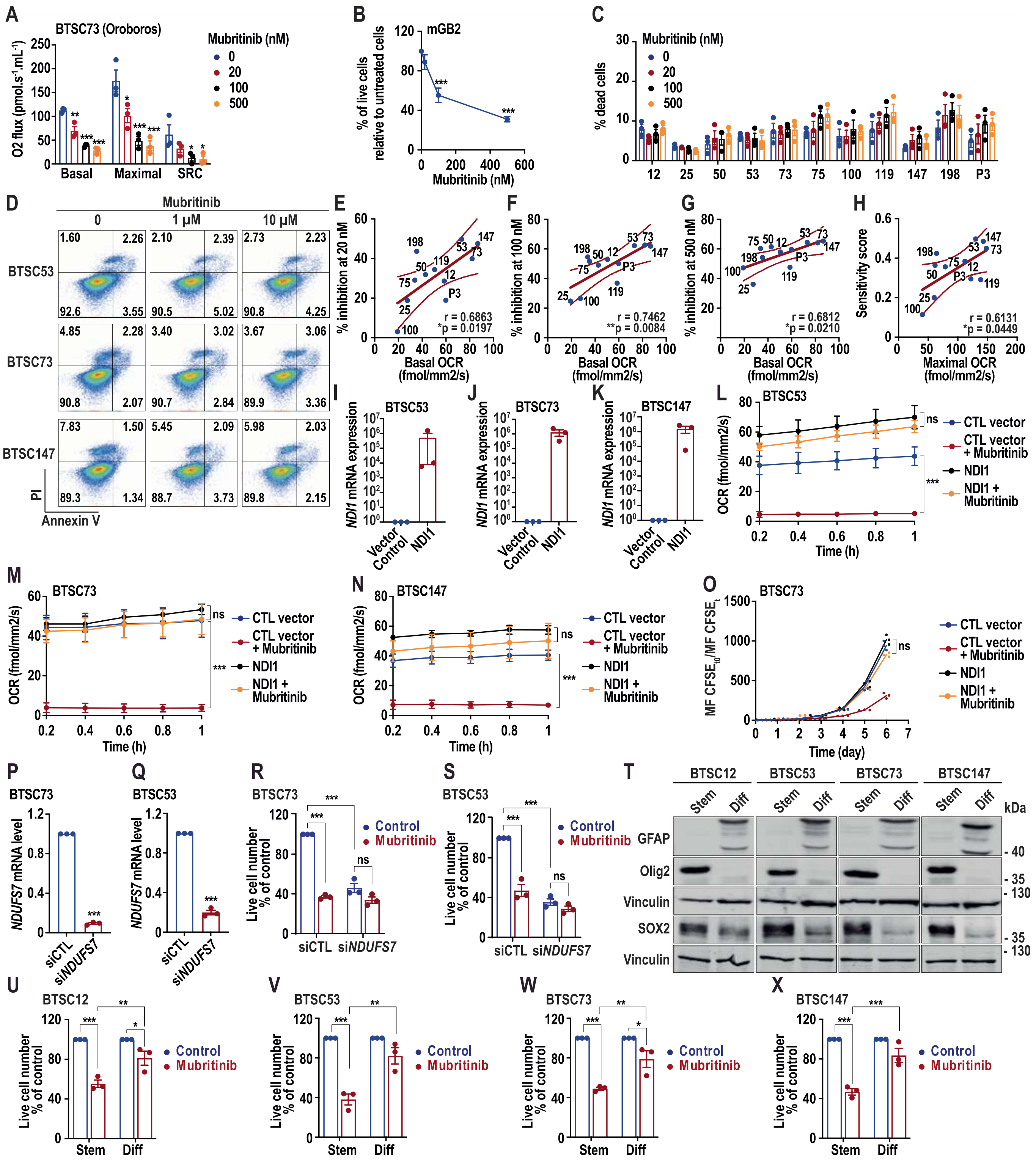
A
BTSC73 (Oroboros)
O2 flux (pmol.s-1.mL-1)
Basal
Maximal
SRC
Mubritinib (nM)
0
20
100
500
B
mGB2
% of live cells relative to untreated cells
Mubritinib (nM)
C
Mubritinib (nM)
% dead cells
12 25 50 53 73 75 100 119 147 198 P3
D
Mubritinib
0
1 µM
10 µM
BTSC53
BTSC73
BTSC147
PI
Annexin V
E
% inhibition at 20 nM
Basal OCR (fmol/mm2/s)
r = 0.6863
*p = 0.0197
F
% inhibition at 100 nM
Basal OCR (fmol/mm2/s)
r = 0.7462
**p = 0.0084
G
% inhibition at 500 nM
Basal OCR (fmol/mm2/s)
r = 0.6812
*p = 0.0210
H
Sensitivity score
Maximal OCR (fmol/mm2/s)
r = 0.6131
*p = 0.0449
I
BTSC53
NDI1 mRNA expression
Vector Control
NDI1
J
BTSC73
K
BTSC147
L
BTSC53
OCR (fmol/mm2/s)
Time (h)
CTL vector
CTL vector + Mubritinib
NDI1
NDI1 + Mubritinib
M
BTSC73
N
BTSC147
O
BTSC73
MF CFSEt0/MF CFSEt
Time (day)
P
BTSC73
NDUFS7 mRNA level
siCTL
siNDUFS7
Q
BTSC53
R
BTSC73
Live cell number % of control
Control
Mubritinib
S
BTSC53
T
BTSC12
BTSC53
BTSC73
BTSC147
Stem
Diff
kDa
GFAP
Olig2
Vinculin
SOX2
Vinculin
U
BTSC12
Stem
Diff
V
BTSC53
W
BTSC73
X
BTSC147

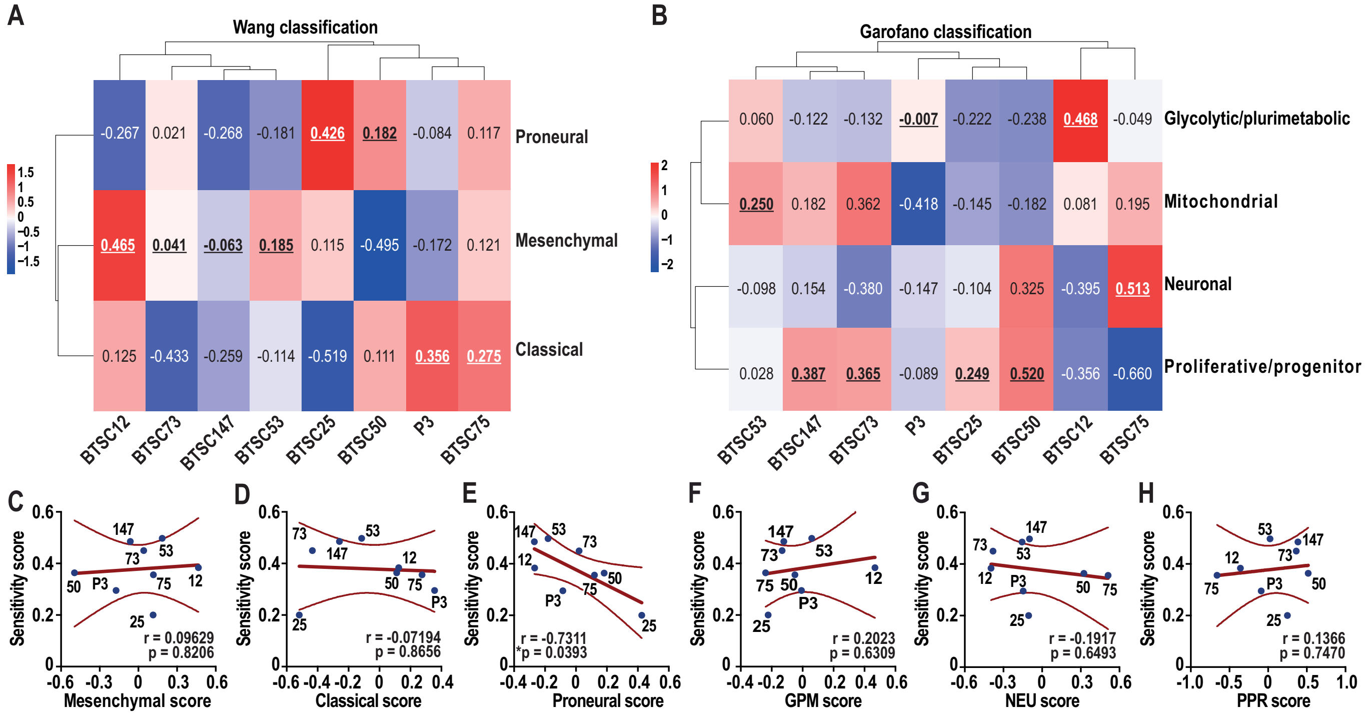

Figure EV2. Transcriptional classification scores of BTSCs and correlation analysis with sensitivity to mubritinib.

(**A**, **B**) Heatmaps illustrating the hierarchical clustering of genes related to Wang (**A**) and Garofano (**B**) glioblastoma transcriptional subtypes. Dominant transcriptional subtype score is underlined and highlighted in bold. (**C–H**) Pearson correlation analysis was performed between the BTSC sensitivity score to mubritinib following 7 days of treatment and mesenchymal (**C**), classical (**D**), proneural (**E**), glycolytic/plurimetabolic (GPM) (**F**), neuronal (NEU) (**G**) and proliferative/progenitor (PPR) (**H**) transcriptional subtype signature scores.

 © The Author(s)

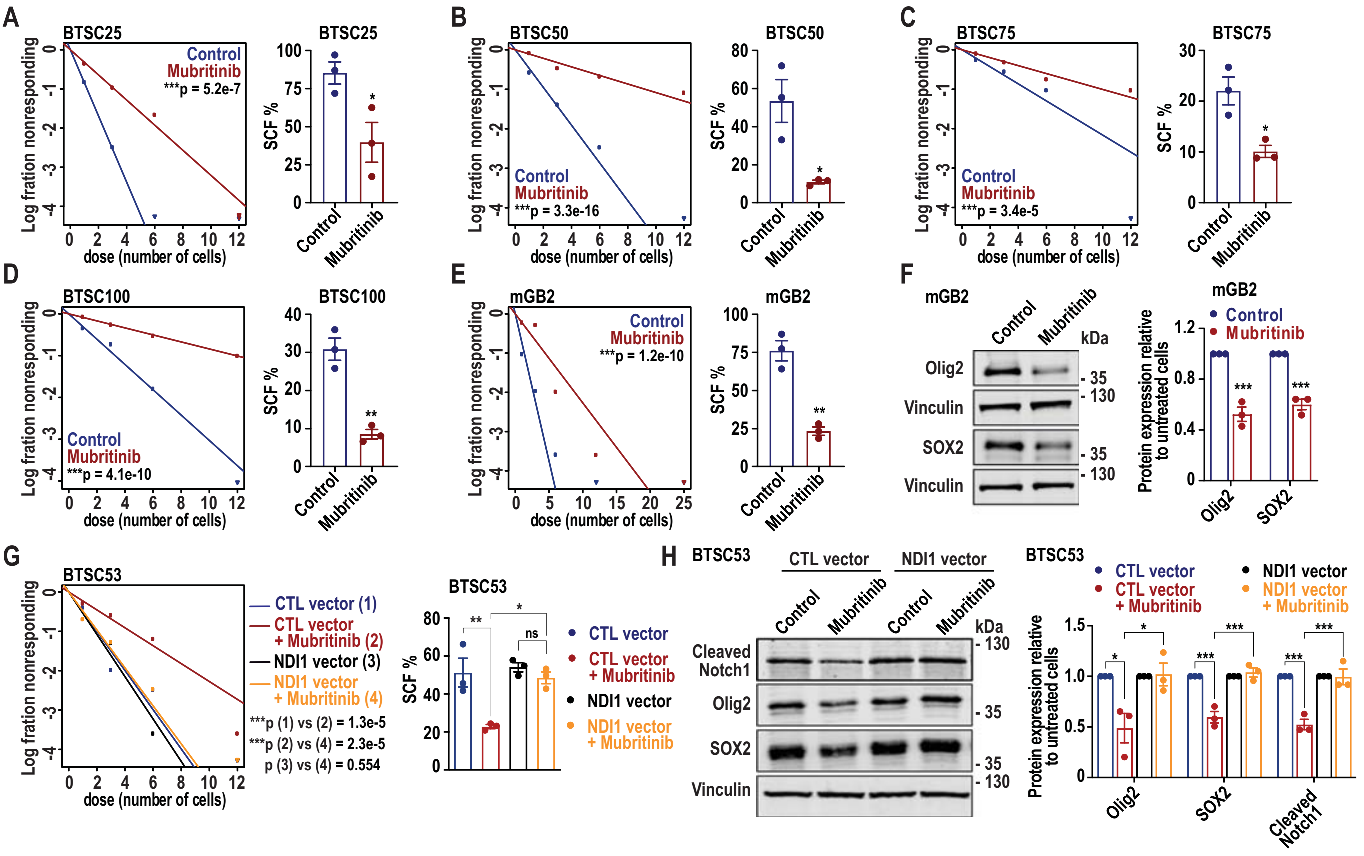

Figure EV3. Validation of the effect of mubritinib on the stemness of murine-derived and additional patient-derived BTSCs.

(A–D) BTSC25 (**A**), BTSC50 (**B**), BTSC75 (**C**) and BTSC100 (**D**) were subjected to ELDA to estimate the SCF, 21 days following treatment with 500 nM mubritinib or vehicle control. Data are presented as the means ± SEM, $n = 3$ independent biological experiments. Chi-square test for ELDA plots and unpaired two-tailed t test for SCF were used. $^{*}p_{BTSC25} = 0.038$, $^{*}p_{BTSC50} = 0.0196$, $^{*}p_{BTSC75} = 0.016$, $^{**}p_{BTSC100} = 0.0022$. **(E)** The murine-derived BTSCs (mGB2) were subjected to ELDA to estimate the SCF, 7 days following treatment with 500 nM mubritinib or vehicle control. Data are presented as the means ± SEM, $n = 3$ independent biological experiments. Chi-square test for ELDA plots and unpaired two-tailed t test for SCF were used. $^{**}p_{mGB2} = 0.0018$. **(F)** mGB2 cells were treated for 4 days with 500 nM mubritinib or vehicle control and subjected to immunoblotting using the antibodies indicated on the blots. Vinculin was used as loading control. Densitometric quantifications of Olig2 and SOX2 protein levels normalized to their corresponding loading controls are presented. Data are presented as the means ± SEM, $n = 3$ independent biological experiments. One-way ANOVA followed by Dunnett's test vs vehicle control. $^{***}p_{Olig2} = 0.0003$, $^{***}p_{SOX2} = 0.0007$. **(G)** BTSC53 transduced with the control (CTL) or NDI1 vector were treated with vehicle control or 500 nM mubritinib and subjected to ELDA to estimate the SCF, 21 days following treatment. Data are presented as the means ± SEM, $n = 3$ independent biological experiments. Chi-square test for ELDA plots and one-way ANOVA followed by Tukey's test for SCF were used. $^{**}p_{CTL\ vs\ mubritinib} = 0.0069$, $^{*}p_{mubritinib\ vs\ NDI1\ +\ mubritinib} = 0.0121$, $p_{NDI1\ vs\ NDI1\ +\ mubritinib} = 0.8008$. **(H)** BTSC53 transduced with the CTL or NDI1 vector were treated with vehicle control or 500 nM mubritinib for 4 days and subjected to immunoblotting using the antibodies indicated on the blots. Vinculin was used as loading control. Densitometric quantifications of cleaved Notch1, Olig2 and SOX2 protein levels normalized to their corresponding loading controls are presented. Data are presented as the means ± SEM, $n = 3$ independent biological experiments. One-way ANOVA followed by Tukey's test. $^{*}p_{Olig2\ (CTL\ vs\ mubritinib)} = 0.0174$, $^{*}p_{Olig2\ (Mubritinib\ vs\ NDI1\ +\ mubritinib)} = 0.0142$, $p_{Olig2\ (NDI1\ vs\ NDI1\ +\ mubritinib)} = 0.9986$, $^{***}p_{SOX2\ (CTL\ vs\ mubritinib)} = 0.0002$, $^{***}p_{SOX2\ (mubritinib\ vs\ NDI1\ +\ mubritinib)} = 0.0001$, $p_{SOX2\ (NDI1\ vs\ NDI1\ +\ mubritinib)} = 0.8669$, $^{***}p_{Cleaved\ Notch1\ (CTL\ vs\ mubritinib)} = 0.0004$, $^{***}p_{Cleaved\ Notch1\ (mubritinib\ vs\ NDI1\ +\ mubritinib)} = 0.0004$, $p_{Cleaved\ Notch1\ (NDI1\ vs\ NDI1\ +\ mubritinib)} > 0.9999$.

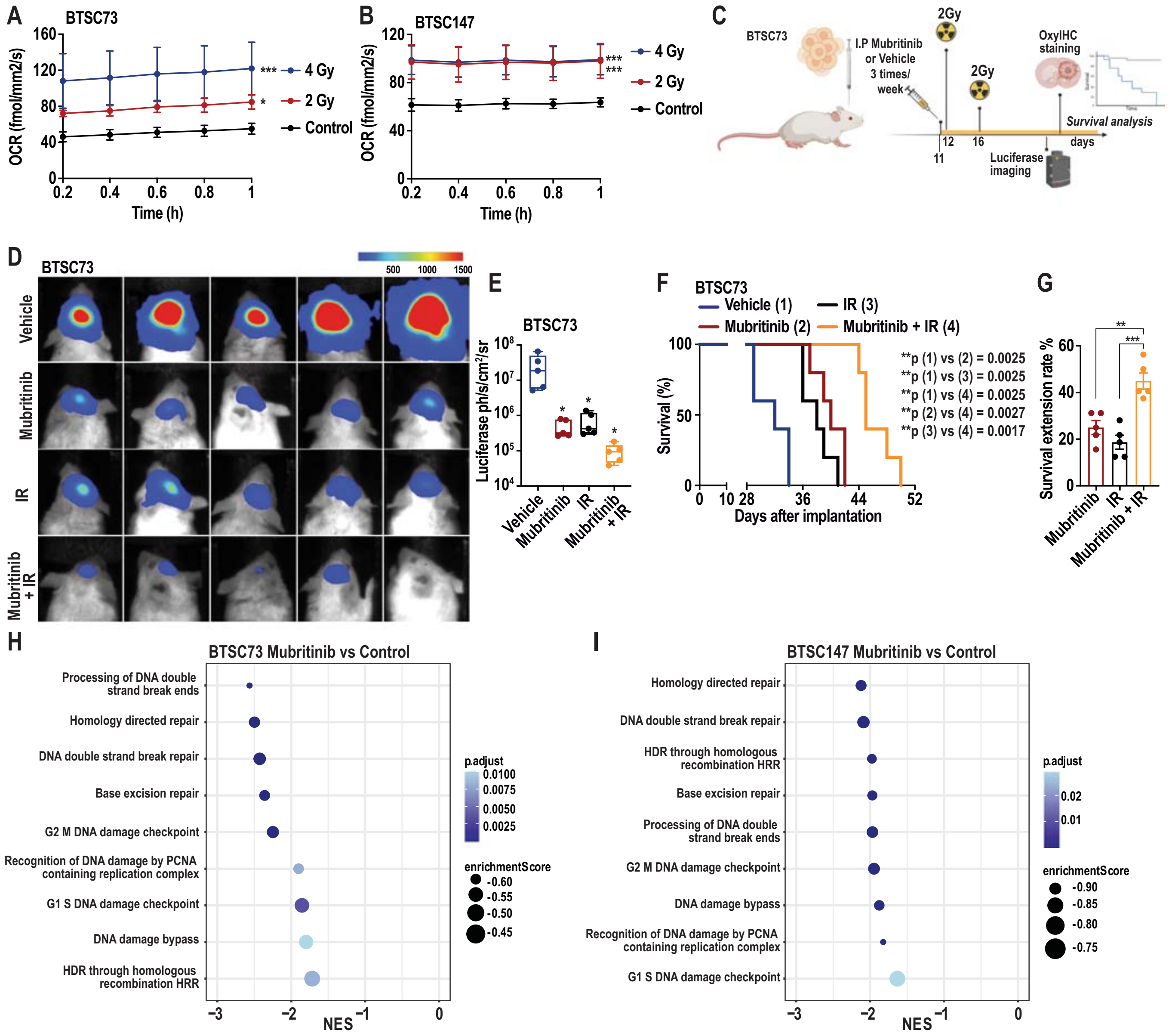

Figure EV4. Mubritinib sensitizes GB tumours to ionizing radiation.

(**A**, **B**) 2 Gy or 4 Gy irradiated BTSC73 (**A**) BTSC147 (**B**) were subjected to real-time Resipher analysis to measure the basal OCR 16 h following irradiation. Data are presented as the means ± SEM, $n = 3$ independent biological experiments. Two-way ANOVA followed by Dunnett's test vs vehicle control. BTSC73 (**A**): $^{*}p_{\text{control vs 2 Gy}} = 0.0357$, $^{***}p_{\text{control vs 4 Gy}} = 6e{-}6$. BTSC147 (**B**): $^{***}p_{\text{control vs 2 Gy}} = 6.8e{-}6$, $^{***}p_{\text{control vs 4 Gy}} = 3.3e{-}6$. (**C**) Schematic diagram of the experimental procedure in which luciferase-expressing BTSC73 cells were intracranially injected into RAGγ2C$^{-/-}$ mice. 11 days after implantation, the mice were randomized into 2 groups: vehicle control or mubritinib (6 mg/kg). Mice were treated 3 times per week (Monday, Wednesday and Friday). For IR groups, 24 h after the first mubritinib injection, mice received IR (2 Gy). On day 16, mice were subjected to another cycle of IR at 2 Gy. (**D**, **E**) Bioluminescence images (**D**) and quantification of luciferase activity (**E**) are presented. Data are presented as box plots showing 25th and 75th percentiles (box), median (centre line), minima and maxima (whiskers), $n = 5$ mice. One-way ANOVA followed by Tukey's test. $^{*}p_{\text{vehicle vs mubritinib}} = 0.0244$, $^{*}p_{\text{vehicle vs IR}} = 0.0258$, $^{*}p_{\text{vehicle vs mubritinib + IR}} = 0.0222$. (**F**) KM survival plot was graphed to evaluate mice lifespan in each group, mice were collected at end stage (log-rank test, $n = 5$ mice). (**G**) Survival extensions of mice bearing BTSC73-derived tumours treated with mubritinib, IR, or mubritinib + IR relative to those treated with the vehicle control were calculated. Data are presented as the means ± SEM, $n = 5$ mice. One-way ANOVA followed by Tukey's test. $^{**}p_{\text{mubritinib vs IR + mubritinib}} = 0.0020$, $^{***}p_{\text{IR vs IR + mubritinib}} = 0.0002$. (**H**, **I**) Gene set enrichment analysis of deregulated genes in BTSC73 (**H**) and BTSC147 (**I**) treated with 500 nM of mubritinib for 24 h demonstrates enrichment of gene sets corresponding to DNA repair pathways. Permutation test was used to calculate *p*-values, which were then corrected using the Benjamini-Hochberg method to obtain adjusted *p*-values (p.adjust).

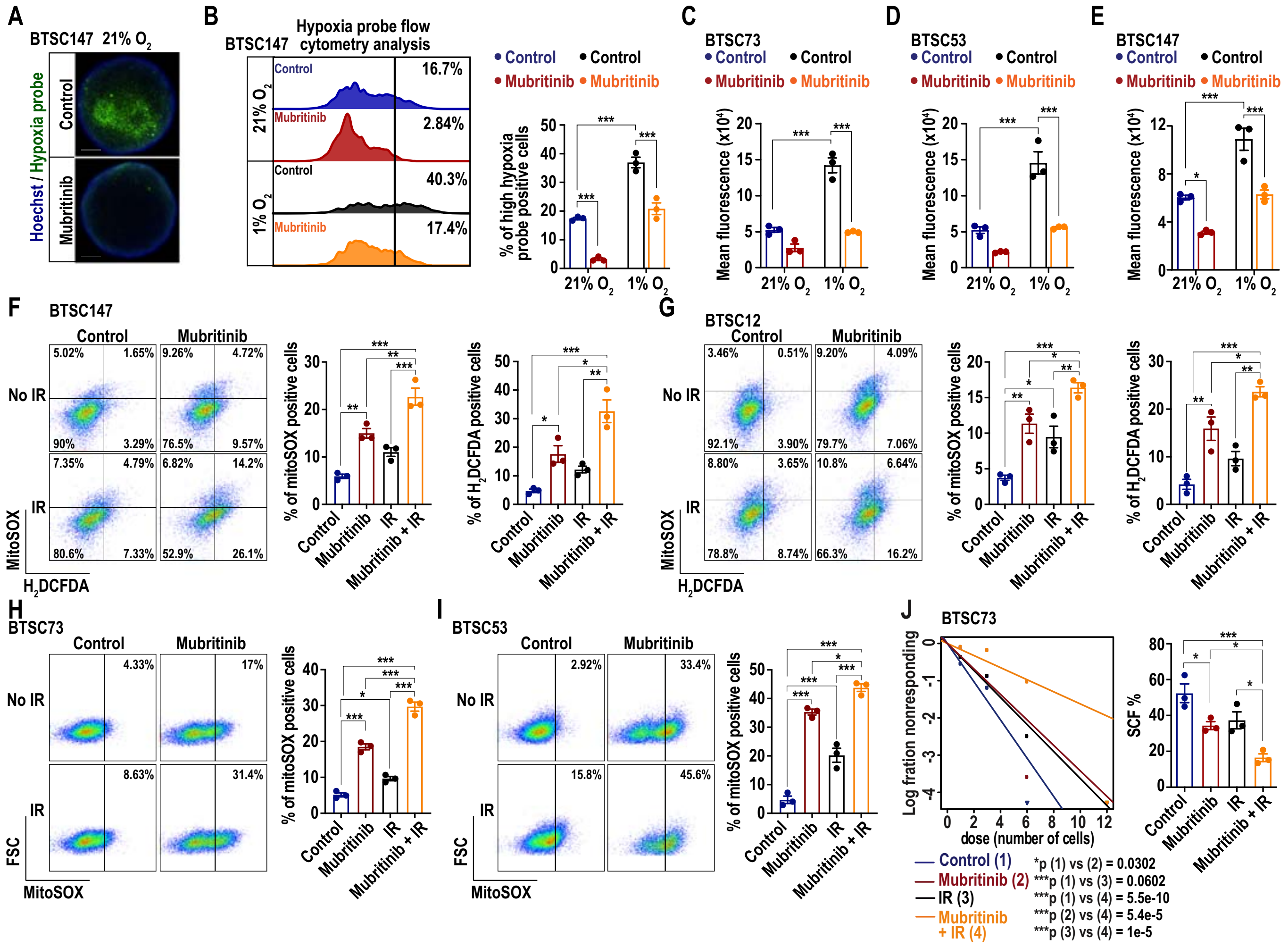

Figure EV5. Mubritinib alleviates tumour hypoxia and enhances oxidative stress to sensitize GB tumours to IR.

(**A**) BTSC147 tumourspheres were treated with 500 nM mubritinib and subjected to labelling with hypoxia probe (green) followed by live fluorescent imaging. Nuclei were stained by Hoechst. Scale bar = 100 µm. (**B**) BTSC147 tumourspheres were treated with 500 nM mubritinib under normoxic (21% O_2) or hypoxic (1% O_2) conditions and subjected to labelling with hypoxia probe followed by flow cytometric analysis. The percentages of high hypoxia probe-positive cells are shown. Data are presented as the means ± SEM, $n = 3$ independent biological experiments. One-way ANOVA followed by Tukey's test. $^{***}p_{control\ 21\%\ O_2\ vs\ mubritinib\ 21\%\ O_2} = 0.0005$, $^{***}p_{control\ 21\%\ O_2\ vs\ control\ 1\%\ O_2} = 7.4e{-}7$, $^{***}p_{control\ 1\%\ O_2\ vs\ mubritinib\ 1\%\ O_2} = 0.0002$. (**C–E**) BTSC73 (**C**), BTSC53 (**D**) and BTSC147 (**E**) tumourspheres were treated with 500 nM mubritinib under normoxic or hypoxic conditions and subjected to labelling with hypoxia probe followed by flow cytometric analysis. The mean fluorescence of hypoxia probe is shown. Data are presented as the means ± SEM, $n = 3$ independent biological experiments. One-way ANOVA followed by Tukey's test. BTSC73 (**C**): $^{***}p_{control\ 21\%\ O_2\ vs\ control\ 1\%\ O_2} = 2.8e{-}5$, $^{***}p_{control\ 1\%\ O_2\ vs\ mubritinib\ 1\%\ O_2} = 2.2e{-}5$. BTSC53 (**D**): $^{***}p_{control\ 21\%\ O_2\ vs\ control\ 1\%\ O_2} = 0.0002$, $^{***}p_{control\ 1\%\ O_2\ vs\ mubritinib\ 1\%\ O_2} = 0.0002$. BTSC147 (**E**): $^{*}p_{control\ 21\%\ O_2\ vs\ mubritinib\ 21\%\ O_2} = 0.0148$, $^{***}p_{control\ 21\%\ O_2\ vs\ control\ 1\%\ O_2} = 0.0006$, $^{***}p_{control\ 1\%\ O_2\ vs\ mubritinib\ 1\%\ O_2} = 0.0009$. (**F, G**) BTSC147 (**F**) and BTSC12 (**G**) tumourspheres were treated with 500 nM mubritinib, IR 2 Gy, or combination of both for 3 days and subjected to labelling with H_2DCFDA and MitoSOX followed by flow cytometric analysis. The percentages of H_2DCFDA or MitoSOX positive cells are presented. Data are presented as the means ± SEM, $n = 3$ independent biological experiments. One-way ANOVA followed by Tukey's test. BTSC147 (**F**): $^{**}p_{mitoSOX\ (control\ vs\ mubritinib)} = 0.0023$, $^{***}p_{mitoSOX\ (control\ vs\ mubritinib\ +\ IR)} = 3.1e{-}5$, $^{**}p_{mitoSOX\ (mubritinib\ vs\ mubritinib\ +\ IR)} = 0.0065$, $^{***}p_{mitoSOX\ (IR\ vs\ mubritinib\ +\ IR)} = 0.0004$. $^{*}p_{H_2DCFDA\ (control\ vs\ mubritinib)} = 0.0287$, $^{***}p_{H_2DCFDA\ (control\ vs\ mubritinib+IR)} = 0.0002$, $^{*}p_{H_2DCFDA\ (mubritinib\ vs\ mubritinib+IR)} = 0.0129$, $^{**}p_{H_2DCFDA\ (IR\ vs\ mubritinib+IR)} = 0.0020$. BTSC12 (**G**): $^{**}p_{mitoSOX\ (control\ vs\ mubritinib)} = 0.005$, $^{*}p_{mitoSOX\ (control\ vs\ IR)} = 0.0237$, $^{***}p_{mitoSOX\ (control\ vs\ mubritinib\ +\ IR)} = 0.0002$, $^{*}p_{mitoSOX\ (mubritinib\ vs\ mubritinib\ +\ IR)} = 0.0453$, $^{**}p_{mitoSOX\ (IR\ vs\ mubritinib\ +\ IR)} = 0.0089$. $^{**}p_{H_2DCFDA\ (control\ vs\ mubritinib)} = 0.0040$, $^{***}p_{H_2DCFDA\ (control\ vs\ mubritinib+IR)} = 0.0001$, $^{*}p_{H_2DCFDA\ (mubritinib\ vs\ mubritinib+IR)} = 0.0386$, $^{**}p_{H_2DCFDA\ (IR\ vs\ mubritinib+IR)} = 0.0013$. (**H, I**) BTSC73 (**H**) and BTSC53 (**I**) tumourspheres were treated with 500 nM mubritinib, IR 2 Gy, or combination of both for 3 days under hypoxic conditions (1% O_2) and subjected to labelling with MitoSOX followed by flow cytometric analysis. The percentages of MitoSOX positive cells are presented. Data are presented as the means ± SEM, $n = 3$ independent biological experiments. One-way ANOVA followed by Tukey's test. BTSC73 (**H**): $^{***}p_{control\ vs\ mubritinib} = 1.7e{-}5$, $^{*}p_{control\ vs\ IR} = 0.0226$, $^{***}p_{control\ vs\ mubritinib\ +\ IR} = 1.3e{-}7$, $^{***}p_{mubritinib\ vs\ mubritinib\ +\ IR} = 6.4e{-}5$, $^{***}p_{IR\ vs\ mubritinib\ +\ IR} = 7.7e{-}7$. BTSC53 (**I**): $^{***}p_{control\ vs\ mubritinib} = 4.5e{-}6$, $^{***}p_{control\ vs\ IR} = 0.0006$, $^{***}p_{control\ vs\ mubritinib\ +\ IR} = 6.6e{-}7$, $^{*}p_{mubritinib\ vs\ mubritinib\ +\ IR} = 0.0241$, $^{***}p_{IR\ vs\ mubritinib\ +\ IR} = 3.2e{-}5$. (**J**) BTSC73 treated with 500 nM mubritinib, irradiated with 2 Gy, and subjected to ELDA, under hypoxic condition (1% O_2), to estimate the SCF. Data are presented as the means ± SEM, $n = 3$ independent biological experiments. Chi-square test for ELDA plots and one-way ANOVA followed by Tukey's test for SCF were used. $^{*}p_{control\ vs\ mubritinib} = 0.0419$, $^{***}p_{control\ vs\ mubritinib\ +\ IR} = 0.0007$, $^{*}p_{mubritinib\ vs\ mubritinib\ +\ IR} = 0.0435$, $^{*}p_{IR\ vs\ mubritinib\ +\ IR} = 0.0206$.

© The Author(s)
